# *In vitro* technology and ADMET research in traditional Chinese medicine

**DOI:** 10.3389/fphar.2025.1605330

**Published:** 2025-07-09

**Authors:** Jinhao Xue, Shiwen Gao, Zhibo You, Shuo Xu, Jiang Zhou, Hai Jiang, Liu Yang

**Affiliations:** Key Laboratory of Basic and Application Research of Beiyao, Heilongjiang University of Chinese Medicine, Ministry of Education, Harbin, China

**Keywords:** traditional Chinese medicine, ADMET, *in vitro* model, Organ-on-chip, organoids

## Abstract

Traditional Chinese medicine (TCM) faces many challenges in the study of absorption, distribution, metabolism, excretion and toxicity (ADME/T) because of its complexity and diversity. *In vitro* models, which can effectively simulate the *in vivo* environment and provide a platform for the analysis of the efficacy and toxicity mechanism of TCMs, play important roles in the study of TCM compounds. This paper evaluates the advantages and limitations of *in vitro* models (including cell models, noncell traditional models, OoC and organoid technology) from the perspective of ADMET. We emphasize the innovative application of these models in analysing the absorption kinetics, brain targeting, complex metabolic pathways, excretion characteristics and potential toxicity of TCM components. This review emphasizes the advantages of *in vitro* methods in meeting the challenges of TCM multicomponent analysis, including the ability to study component interactions and screen potential drug candidates. In addition, we carried out a special in-depth analysis of OoC and organoid technology and systematically explored their unique advantages in the field of traditional Chinese medicine. Moreover, combined with bibliometric analysis, the literature on the Chinese medicine ADMET published *in vitro* in recent years was statistically summarized, and the hot spots and trends of TCM in this research were analysed.

## 1 Introduction

ADMET research is crucial to drug development, whereby systematic evaluation of drug behavior *in vivo* can reveal the bioavailability, tissue distribution, and toxicity characteristics of metabolites, help to identify potential side effects and drug interactions, optimize drug chemical structures, and improve therapeutic outcomes while reducing the risk of adverse reactions.

In research focusing on the absorption, distribution, metabolism, excretion, and toxicity (ADMET) of traditional Chinese medicine (TCM), the intricate interactions among its components, derived from various plants, animals, or minerals, contribute to its complexity. This complexity results in significant differences in the metabolic and toxicological characteristics of TCMs compared with those of synthetic drugs, primarily due to the production of numerous metabolites and a more elaborate biotransformation pathway. Understanding drug metabolism is crucial for toxicity assessment, as metabolic processes not only influence drug efficacy but also present potential risks of increased toxicity (Yu et al., 2019). Moreover, prolonged use of TCM may prevent the detection of chronic toxicity and carcinogenic effects through short-term safety assessments, highlighting the importance of comprehensive evaluations of long-term safety (Zhao et al., 2024). Some metabolites may have stronger toxicity or adverse reactions than their prototypes do, which increases the safety hazard of drug use. Its complex components and metabolites make it much more difficult for TCM to identify metabolites and screen toxic substances. The study of the absorption, distribution, and excretion of TCMs presents numerous challenges. The diverse and complex nature of TCM components hinders the prediction of their intestinal absorption. Variations in transport mechanisms lead to different components entering the bloodstream, while interactions with intestinal microorganisms can alter their bioavailability. Furthermore, factors such as plasma protein binding and tissue affinity complicate the distribution of TCM components in the body, making it challenging to assess their concentration and effects in various tissues. The excretion of TCM involves a complex process influenced by multiple transporters, particularly efflux transporters such as P-glycoprotein (P-gp), which can impede the accumulation or elimination of drugs in the body.

In this context, *in vitro* models have become crucial in TCM research. *In vitro* models (such as Caco-2 cells and liver microsomes) can exclude interference factors from *in vivo* environments, independently studying the effects of specific metabolic enzymes and transport proteins on Chinese herbal components. Compared with *in vivo* animal experiments, standardized culture conditions improve experimental reproducibility and reduce batch-to-batch variations. *In vitro* models offer the advantages of low cost and high efficiency, enabling simultaneous detection of metabolic stability for multiple components. Moreover, these models comply with animal welfare guidelines, reducing animal usage. With the development of organoid and OoC technologies, specific models targeting individual and disease states can be established, reflecting metabolic differences across different genetic backgrounds and guiding precision medicine. For example, the use of iPSC-induced hepatocytes or intestinal organoids can reflect individual variations in herbal medicine metabolism across different genetic backgrounds, guiding precision medication (Wang J et al., 2023; Wang X et al., 2023; Marrero et al., 2021). Liver organoid chips can be used to investigate the distribution and toxicity characteristics of herbal components in pathological microenvironments (Liu et al., 2024; Lee et al., 2021).

## 2 *In vitro* intestinal absorption model

The small intestine is the main site where nutrients, water, electrolytes and exogenous organisms are absorbed (Zhu et al., 2024). When evaluating drug absorption, we often predict the intestinal absorption of drugs. The methods commonly used in *in vitro* intestinal absorption experiments include everted intestinal sac models, cell culture models, the chamber system, and parallel artificial membrane permeation experiments (PAPAMAs).

### 2.1 Everted intestinal sac model

**FIGURE 1:**
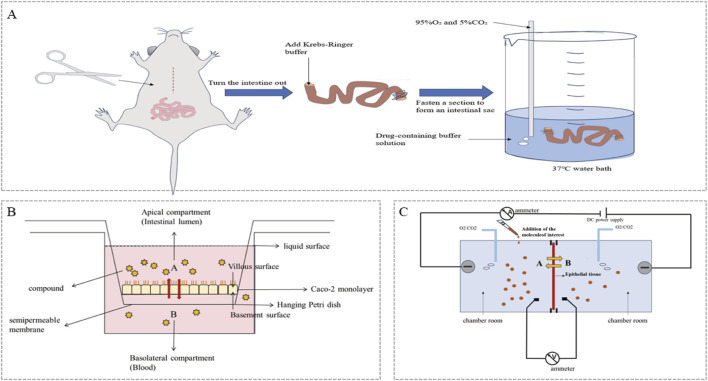
Schematic diagram of the *in vitro* intestinal absorption model: **(A)** Rat everted intestinal sac model; **(B)** Caco-2 monolayer cell model (A-Apical compartment; B-Basolateral compartment, A-B: Direction of drug absorption; B-A: Indicates that the drug has efflux transport; **(C)** Ussing chamber system (A-administration side; B-sample side, A-B: Direction of drug absorption; B-A: Indicates that the drug has efflux transport).

A model of the everted intestinal sac (Figure 1A) was first proposed by Wilson and Wiseman (1954). Subsequently, Barthe L et al. developed and verified an improved everted intestinal sac system, which was used to study the bypass transport of cells in the rat small intestine. Tissue culture medium (TC 199) was used instead of simple saline solution to improve the viability and structural integrity of the tissues (Barthe et al., 1998). Ugolev Am et al. also improved the eversion technique. By introducing the design of bilateral oxygen ventilation, the *in vivo* environment can be simulated more accurately, and the activity of small intestine tissue *in vitro* can be maintained (Ugolev et al., 1980). The improved everted intestinal sac model has been widely used to study drug absorption mechanisms and kinetics *in vitro* (Alam et al., 2012).

### 2.2 Cell culture model

The first cell model used to estimate cell absorption was the Caco-2 cell line, which was originally established in 1977 (Fogh et al., 1977). Subsequently, cell models have been used for drug absorption evaluation. In addition to Caco-2 cells, commonly used cell lines include the HT29-MTX cell model, MDCK cell model and MDCK-MDR1 cell model.

#### 2.2.1 Caco-2 cell model

In the past thirty or forty years, Caco-2 cells have been widely studied, and attention has been given to these cells, which have been applied as models for predicting the intestinal absorption of drugs. Caco-2 (Figure 1B) has become an important tool for permeability classification in the practice of bioequivalence exemption based on BCS classification. This model is widely adopted in the scientific research field and is used mainly to simulate the absorption and transport process of specific compounds in the human small intestine. Furthermore, the transport mechanism of these chemical components has been explored in detail (He et al., 2018). Both domestic and international studies have reported the use of the Caco-2 cell model to investigate the absorption of TCM in the human small intestine, with a particular prevalence of alkaloid and flavonoid substances. For example, Zheng et al. (2015) utilized the Caco-2 cell model to study the transport mechanism of flavonoid components in Buyang Huanwu soup, discovering that they are primarily transported via passive diffusion; Liao et al. (2011) employed the Caco-2 cell model to explore the absorption mechanism of andrographolide, finding that it may be mediated by active transport carriers. The Caco-2 cell model can be used to study the intestinal absorption and efflux transporter effects of TCM components, providing a basis for designing oral TCM formulations.

#### 2.2.2 MDCK cell model

The MDCK (Madin‒Darby canine kidney) cell line is derived from the renal epithelial cells of Martin‒Darby dogs, and it is a cell line with extremely close intercellular connections. This cell line shows low levels of transporter expression and low metabolic activity (Braun et al., 2000). There is a good correlation between the MDCK cell line and the Caco-2 cell line in the study of the permeability of active absorption drugs (Irvine et al., 1999). Many physiological characteristics of the MDCK cell line are highly similar to those of the blood‒brain barrier (BBB), so it is often used as a model for screening drugs through the BBB *in vitro* (Veronesi, 1996). In 1988, the scientists of Pastan et al. (1988) successfully transfected the human mdr1 gene into MDCK cells, thus establishing the MDCK-MDR1 cell line, which can express high levels of P-gp. MDCK-MDR1 cells have a relatively short culture cycle, can obtain many cell samples in a short time, and can maintain a high degree of uniformity in the process of continuous passage (Polli et al., 2001). Therefore, the MDCK-MDR1 cell model is an ideal model for determining the protein and gene expression of P-gp. Zheng et al. (Liao et al., 2016) took advantage of the high expression of P-gP in MDCK-MDR1 cells and verified that IMP enhanced the intestinal absorption of puerarin and vincristine by inhibiting the activity of P-gP and the expression of its mRNA and protein.

#### 2.2.3 HT29-MTX cell model

The HT29-MTX cell line is a derivative cell line obtained by methotrexate treatment of parent HT29 cells (Lesuffleur et al., 1991). HT29-MTX cells can differentiate into mucus-secreting goblet cells, simulating the mucous layer of the small intestine, which is beneficial for studying the effect of intestinal mucus on drug absorption. Compared with the Caco-2 model, HT29-MTX significantly improved the absorption capacity of lipophilic compounds due to the characteristics of goblet cells (Awortwe et al., 2014). When HT29-MTX cells and Caco-2 cells are cocultured, the permeability of compounds absorbed by passive transport is usually much greater than that of the Caco-2 monolayer model, which more accurately reflects the absorption characteristics of drugs in the intestine (Hilgendorf et al., 2000; Behrens et al., 2001). However, as the proportion of HT29-MTX cells in the Caco-2 and HT29-MTX coculture models increases, transepithelial electrical resistance (TEER) decreases significantly (Chen et al., 2010; Pan et al., 2015).

### 2.3 The chamber system

**FIGURE 2:**
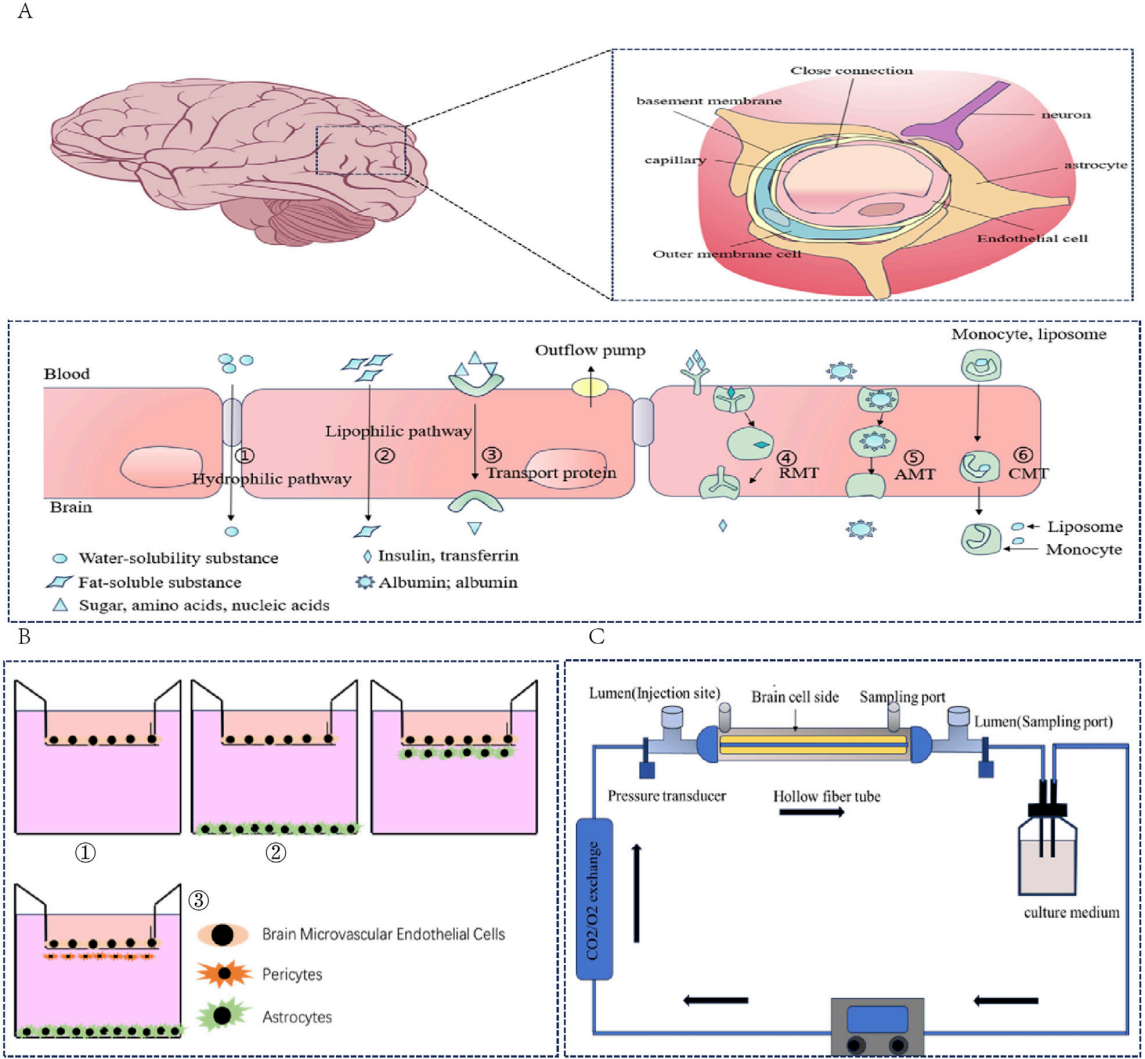
**(A)** Composition and transport mechanism of the blood‒brain barrier: blood‒brain barrier structure; transport mechanism of different types of compounds in the blood‒brain barrier. **(B)** Schematic diagram of BBB cell culture. ① Single culture model; ② Double coculture model; ③ Triple coculture model. **(C)** Schematic diagram of the dynamic model of the fibre cavity.

The Chamber (Figure 1C) is a high-precision *in vitro* model that is mainly used to study the transmembrane transport and electrophysiological characteristics of drugs in epithelial cells. The system can be used to evaluate drug absorption efficiency by measuring the performance of a semipermeable membrane and current, as shown in Figure 2. It is suitable for animal tissues or cultured epithelial cells, such as Caco-2 cells, and has important value in simulating drug absorption and metabolism. Scherbl et al. (2014) used this model to simulate the intestinal mucosal environment, explored the transport of caffeic hydroxycinnamic acid and its esters, and revealed the influence of compound structure on the absorption mechanism. Compared with the Caco-2 cell model, the use of isolated intestinal tissue segments can better represent the complex *in vivo* morphology (multicell aggregation and the existence of a mucus layer). This approach can better represent various possible processes involved in the *in vivo* situation (Westerhout et al., 2015). Wang et al. (2022) collected eight kinds of traditional Chinese medicines, including *Salvia miltiorrhiza* and *Astragalus propinquus*. Three intestinal absorption models, the Caco-2 cell model, the overturned rat intestinal sac model and the Ussing chamber model, were established. Combined with the three models and the absorption of drugs by oral gavage, the potential effective components screened by the Uxin chamber model are completely consistent with those determined by the rat gavage method. The results of the Ussing chamber model are the closest to the actual absorption of Chinese herbal extracts.

### 2.4 *In vitro* models of intestinal absorption

Other *in vitro* models of intestinal absorption include the parallel artificial membrane osmotic model (PAMPA), drug dissolution/absorption bionic system (DDASS), and intestinal organ and intestinal epithelial organ models derived from intestinal hepatocyte differentiation. The PAMPA model consists of a donor chamber and a recipient chamber separated by an artificial membrane. In this artificial membrane, organic lecithin solution was coated on a polyvinylidene fluoride (PVDF) membrane to form a stable lipid bilayer, thus better simulating the structure of the biofilm (Song et al., 2023). The PAMPA is fast, efficient and economical and is especially suitable for early drug screening and preparation optimization, which can save time and resources. The DDASS system can continuously and dynamically evaluate the dissolution and transmembrane permeation characteristics of drugs (Gu et al., 2015).

### 2.5 Application status of the *in vitro* absorption model in TCM research

Compared with the single-component research model typical of Western medicine, studies on TCM usually adopt a stepwise reduction approach: “compound formula → herb pair → single herb → herb components → single compound.” This characteristic means that ADMET research in TCM must address a large number of individual compounds. For example, the Chinese Natural Product Database (CNPD) contains more than 57,000 natural products; the Waters 2023 database of TCM natural products covers over 17,000 natural compounds; and the Traditional Chinese Medicine Database (TCMD) comprises 23,033 TCM compounds. With the rapid advancement of modern analytical technologies, data on TCM components are continually expanding. However, not all of these compounds have the same research value—most are present only in trace amounts in medicinal materials, their isolation can be complex, and they are often difficult to obtain in quantities sufficient for *in vivo* evaluation (Li et al., 2015). In this context, *in vitro* ADMET models can play a crucial role in efficiently screening and identifying candidate molecules with drug potential.

Under this background, *in vitro* experiments can quickly evaluate the absorption potential of a large number of samples by virtue of their efficient Qualcomm screening ability, low drug demand and simplified experimental steps, thus accelerating the research and development process of TCMs. Taking the Gegen-Qinlian Decoction as an example, Wang Q. et al. (2017) and Wang R. et al. (2017) used a Caco-2 cell monolayer model to evaluate the intestinal permeability of 42 bioactive compounds and revealed the interactions between components in the formulation. Moreover, compared with the complex reactions of TCMs *in vivo*, *in vitro* models can be used to evaluate the absorption mechanism of TCM components under controlled conditions through cell culture, permeability experiments and other technologies. Moreover, the *in vitro* model can simulate interactions in the physiological environment and provide a platform for studying the synergistic or inhibitory effects between components. This is especially important for the study of the compound prescription of traditional Chinese medicine because different components in the compound prescription may have an impact on absorption. For example, Huang et al. (Huang et al., 2016) successfully analysed the absorption characteristics of the active ingredients in the TCMs of *Salvia miltiorrhiza* and *Panax notoginseng* via Ussing Chamber technology and LC‒MS/MS and revealed that the synergistic administration of *Salvia miltiorrhiza* and *Panax notoginseng* could significantly improve the absorption efficiency of the active ingredients in the jejunum segment.

The most commonly used intestinal absorption models can evaluate drugs from three aspects: absorption capacity, absorption mode and transport. However, each model is different in terms of specific application and accuracy, and a single model has many limitations, The advantages and disadvantages of the absorption model are supplemented in Supplementary Table S1. Therefore, for the study of TCMs and their components, different *in vitro* and *in vivo* models should be selectively used and combined, and various experimental methods and techniques should be combined to evaluate the intestinal absorption characteristics and pharmacodynamic characteristics of drugs more comprehensively.

## 3 *In vitro* distribution model

At present, the drug‒plasma protein binding model and blood‒brain barrier model have been well studied *in vitro*. Including the drug‒plasma protein binding model and blood‒brain barrier model. However, compared with the *in vivo* model, the *in vitro* model is irreplaceable in the study of organ-level distribution quantification (the *in vivo* model can accurately capture the distribution of drugs in inert tissues such as the brain, fat and bone through real-time monitoring *in vivo*, such as PET‒CT imaging and organ biopsy). Therefore, the *in vitro* model is still more appropriate for related experiments on tissue distribution.

### 3.1 Drug‒plasma protein binding model

There are two main kinds of *in vitro* methods for the study of drug‒plasma protein binding: one is to directly determine the concentration of free drugs, such as equilibrium dialysis and ultrafiltration; the other involves various physical and chemical techniques, such as spectroscopy and mass spectrometry, which can provide confirmation of binding sites and structural variation after macromolecules bind ligands (Qiang, 2011). A comparison between the models is shown in Supplementary Table S1.

### 3.2 Blood–brain barrier (BBB) model

TCM has unique advantages and potential for improving BBB function and promoting drug delivery to the brain. An *in vitro* blood‒brain barrier model can be used to screen for effective substances in traditional Chinese medicine that can promote BBB permeability. This technique can be used not only to evaluate the influence of TCM and its components on BBB permeability but also to reveal the distribution and transport mechanism of traditional Chinese medicine in the BBB.

As important selective biological barriers (Figure 2A). The blood‒brain barrier plays a key role in protecting the central nervous system and maintaining brain homeostasis. Therefore, whether drugs can successfully cross the blood‒brain barrier becomes a necessary prerequisite for the treatment of central nervous system diseases when they are administered systematically (Yin and Wang, 2024). Therefore, establishing an appropriate *in vitro* blood‒brain barrier model is highly important for scientifically evaluating the potential and efficacy of drugs for treating brain-related diseases.

#### 3.2.1 Construction of the BBB model: selection of endothelial cell types

Although the formation of the blood‒brain barrier involves the participation of many cells, specialized endothelial cells are undoubtedly its core component. They are responsible for forming the main structural basis of the blood‒brain barrier and express a variety of matrix-specific transporters to regulate the transport of substances between blood and brain tissue (Zhao et al., 2015). Therefore, endothelial cells are an indispensable cell type when constructing a blood‒brain barrier (BBB) model.

##### 3.2.1.1 Primary endothelial cells

Primary endothelial cells are obtained directly from the tissues or organs of organisms through protease digestion or mechanical separation. They have not been subcultured or subcultured only a limited number of times. These cells are directly derived from organisms. Therefore, their biological characteristics, gene expression patterns and interactions between cells are highly consistent with the internal environment (Sabbagh and Nathans, 2020). However, the separation of microvascular endothelial cells involves complicated steps, is time-consuming and laborious, and usually, the yield is limited. Culture conditions may change the transcriptional activity of primary endothelial cells, thus altering some functions of cells (Sabbagh and Nathans, 2020). In addition, the separation of primary cells risks contamination by other primary cell types, which affects the repeatability of the experimental results. With increasing passage number, primary cells may lose their phenotypic characteristics. Therefore, although primary cells have excellent functions, their shortcomings limit their applicability in establishing blood‒brain barrier models (Andjelkovic et al., 2020).

##### 3.2.1.2 Immortalized endothelial cells

The use of primary cells from humans can avoid the genetic variation of drugs between species. However, owing to ethical reasons, the availability of these primary cells is greatly limited. Immortalized human brain capillary endothelial cells can effectively solve this problem. Immortalized endothelial cells obtain infinite proliferation ability through gene modification, which prevents the aging and apoptosis of primary cells. This enables them to be maintained in the laboratory for a long time and is suitable for long-term research projects. Moreover, immortalized cell lines usually have stable genomic and phenotypic characteristics, which reduces the data fluctuations caused by changes in cell characteristics in experiments. In addition, the cell does not need continuous separation and culture, which reduces the complexity of operation and the technical requirements for experimenters. Because of its many advantages, it can not only be cultivated on a large scale to meet the needs of Qualcomm screening and large-scale experiments but also reduce the overall cost of experiments.

##### 3.2.1.3 Stem cells

Stem cells can obtain any specific cell phenotype and can be used in regenerative medicine, *in vitro* disease modelling (Israel et al., 2012; Dolmetsch and Geschwind, 2011; Vatine et al., 2017), *in vitro* blood‒brain barrier modelling (Li Y et al., 2019) and other applications after induced differentiation. These findings provide new research ideas for the study of *in vitro* blood‒brain barrier models, which are more closely related to the human microenvironment. Induced pluripotent stem cells (iPCs), embryonic stem cells and neural progenitor cells have been used to establish a human BBB model *in vitro* (Kaisar et al., 2017). BMEC-like cells derived from human pluripotent stem cells (hPSCs) summarize many functions and molecular features of BMECs *in vivo* and significantly improve our understanding of the development and function of the blood‒brain barrier (Lippmann et al., 2020). However, because of the limitations of differentiation methods, many models lack some phenotypes and functions of BMECs *in vivo*. For example, key adhesion molecules are involved in immune cell migration, some transporter activities and responses to inflammatory stimuli (Park T. E. et al., 2019; Nishihara et al., 2020).

#### 3.2.2 Single model and coculture model based on a Transwell device

The Transwell experimental system consists of upper and lower double-layer chambers separated by microporous semipermeable membranes made of polyester or polycarbonate (pore size range 0.2–4.0 μm), which provide a physical isolation and material exchange interface for the construction of an *in vitro* model of the BBB. On the basis of differences in cell composition, the BBB model can be divided into two systems: single culture and coculture.

The single culture model (Figure 2B) uses only brain microvascular endothelial cells (BMECs) as the main barrier, which has the advantages of simple experimental operation, controllable cost and suitability for Qualcomm drug screening. However, owing to the lack of paracrine regulation of key components of the neurovascular unit (NVU), such as astrocytes and peripheral cells, maintaining the polarization characteristics and functional protein expression of the BBB for a long period of time is difficult (Qi et al., 2023; Cecchelli et al., 2007).

In double-layer coculture (Figure 2B), BMECs are usually cocultured with astrocytes or pericytes, and helper cells can be seeded on the outer side (the bottom of the culture plate) or the top side (the other side of the membrane) of the Transwell system. In addition, the expression of tight junction proteins in endothelial cells is regulated by paracrine signals. Triple cocultures (Figure 2B) integrate BMECs, pericytes and astrocytes and can introduce neurons or microglia when necessary to simulate the plasticity regulation of the BBB dynamically under physiological or neuroinflammatory conditions (Nakagawa et al., 2009; Tontsch and Bauer, 1991). Compared with single cultures, the coculture model has a greater TEER value and lower permeability and can express more specific transporters, which better simulate the characteristics of the BBB. It can be widely used in the research fields of new drug screening, drug permeability evaluation, intercellular interactions and brain disease models.

#### 3.2.3 Dynamic *in vitro* model

The dynamic *in vitro* (DIV) model of the blood‒brain barrier is a three-dimensional model (Figure 2C). Coculture can be realized in this model. In addition, intracavity flow can be generated through the support of artificial capillary-like structures within the framework of this system (Bussolari et al., 1982; Cucullo et al., 2008). BMECs were implanted in the cavity outside the hollow fibre in the sealed cavity, whereas the neurovascular unit (NUV) was implanted in the cavity of the hollow cavity. Pulsating pumps with variable speeds provide a flow rate in the cavity, which can be adjusted to produce medically equivalent pressure in the cavity or shear stress in the capillary in the body (Bussolari et al., 1982). This model has been used to study the pathophysiology of various central nervous system diseases. On the other hand, there are several limitations associated with this model. For example, 9–12 days are needed to reach stable transendothelial electrical resistance (TEER), which is significantly longer than the culture period of coculture models. In addition, it impedes direct observation of endothelial cell morphology from the luminal side, and its construction consumes a large number of cells. These limitations make the model difficult to apply for high-throughput compound screening (Bussolari et al., 1982), although it can still be used for lead compound optimization.

### 3.3 Application status of the *in vitro* distribution model in TCM research

The interaction between the multicomponent characteristics of TCM and the plasma protein binding rate between active components has been widely studied. Wen et al. (2007) studied the binding of the whole components of Danggui Buxue decoction with bovine serum albumin (BSA) via microdialysis and reported that the components in the decoction significantly affected their binding with BSA. Similarly, Qian et al. (2008) studied the interaction between the whole active substance of *Lonicera japonica* Thunb. and bovine serum albumin and reported that the degree of binding of three components, such as chlorogenic acid, to BSA was lower than that of individual components, whereas caffeic acid and rutin were the opposite.

In the study of the plasma protein binding rate, balanced dialysis has always been the gold standard for studying drug‒plasma protein binding. Bao et al. (2019) systematically evaluated the difference in the protein binding rates of nine active components in *Duhaldea cappa* extract in rat/human plasma via this method, revealing significant differences in interspecific binding characteristics. CHen et al. (2017a) determined the plasma protein binding rates of the cardiac glycosides in *Periploca sepium Bunge*, and the results revealed that the plasma protein binding rates of the transformed components increased. However, although this method is mature, its status is challenging because of its time-consuming nature and dilution effect. In addition, ultrafiltration, microdialysis, HPFA and other methods also have their own shortcomings and cannot completely replace the balanced dialysis method. At present, the research focus has shifted from simple drug binding rates, such as binding sites, conformational changes and interactions with other molecules, to more in-depth mechanistic exploration, which requires the combination of many advanced technologies, such as circular dichroism (CD), surface plasmon resonance (SPR), molecular docking and molecular dynamics simulation. For example, Yeggoni et al. (2017) integrated surface plasmon resonance (SPR) and fluorescence quenching techniques combined with molecular docking simulations to elucidate that andrographolide (ANDR) binds to subdomain IIA of human serum albumin (HSA) through hydrophobic interactions and enhances the stability of the complex via α-helix rearrangement.

Many traditional Chinese medicines can increase the permeability of the blood‒brain barrier, such as Borneol, *Acorus gramineus*, *Moschus*, *Ligusticum chuanxiong hort.*, *Benzoinum* and *Storesin* (Fang et al., 2023). However, the mechanism by which ascending drugs are directed in some traditional Chinese medicines is still unclear, and the *in vitro* BBB model provides an effective tool to clarify this mechanism. By constructing an *in vitro* BBB model composed of BMECs and astrocytes, Fan et al. (2015) investigated the influence of borneol on the transport of known P-gp substrates across the BBB and reported that borneol inhibited P-gp function in BMECs through the drug mechanism of NF-*κ*B signal transduction and affected BBB permeability. Wang et al. (2015) cocultured astrocytes (ACs) and human umbilical vein endothelial cells (ECV304) to simulate a BBB model *in vitro*. Muscone can affect BBB permeability by inhibiting the expression of P-gp and MMP-9. In addition, the BBB model is widely used to screen bioactive components with good brain delivery potential. For example, Liu et al. (2017) used a blood‒brain barrier model and reported that Magnolol significantly increased the TEER of endothelial cells, which indicates that magnolol has a significant protective effect on the BBB. As proof, in a coculture model of hCMEC/D3 cells and immortalized human astrocytes (HEBs), Liang (2020) reported that *ginkgo biloba L.* extract bilobalide can reversibly increase BBB permeability; although it does not affect the expression of tight junction proteins, it affects their ultrastructure and promotes ERM/MLC phosphorylation, which provides a new idea for developing brain-targeted drugs. The advantages and disadvantages of the *in vitro* BBB model are compared in Supplementary Table S1.

Although *in vitro* models play an important role in simulating drug plasma protein binding and blood-brain barrier permeability, they have inherent limitations and cannot be used to study the targeted distribution of drugs within organs. The main reason is that *in vitro* models typically focus on individual cells, tissues, or organs, lacking systemic blood circulation and inter-organ connections, and therefore cannot replicate the *in vivo* process of drug distribution via the cardiovascular system. Furthermore, *in vitro* models are unable to accurately mimic the complex physiological barriers and the coordinated regulation of various transport proteins and enzymes that exist *in vivo*, all of which are crucial for tissue distribution. More importantly, the collaborative actions among multiple organs, the dynamic metabolic and distribution processes, and the unique microenvironments of different tissues significantly affect drug distribution in the body, yet these factors are difficult to fully reproduce *in vitro*. Therefore, while *in vitro* models are suitable for evaluating drug absorption and metabolism, they cannot accurately reflect the distribution and targeting of drugs among tissues and organs *in vivo*, making comprehensive animal studies or clinical research indispensable for such investigations.

## 4 *In vitro* liver metabolism model

The *in vitro* liver metabolism model is a key tool for studying the mechanism and toxicity of drugs. At present, the model system covers traditional models such as liver microsomes and primary hepatocytes and advanced technologies such as liver bioreactor systems and microfluidic organ chips. The latter significantly improves the durability and authenticity of metabolic function by integrating hemodynamics and multicell interactions. In addition, in the field of TCM metabolism research, the intestinal flora, as the second genome of the human body, is widely involved in the biotransformation process of active components of traditional Chinese medicine and has become a new hot spot in the basic research of the pharmacodynamic substances of TCM.

### 4.1 Recombinant enzyme technology

**FIGURE 3:**
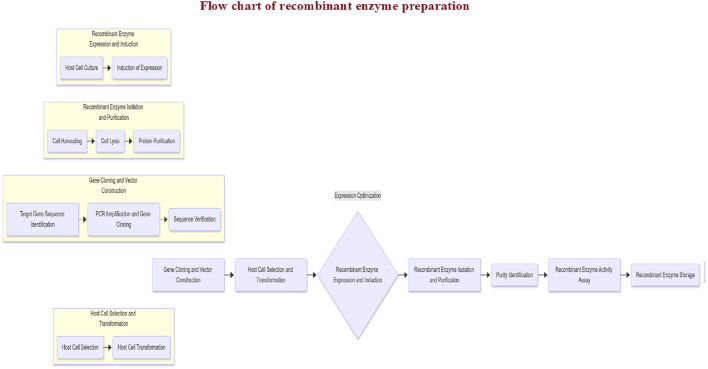
Preparation process for the recombinant enzyme.

Genetic engineering enables the integration of P450s or other enzyme-encoding genes into mammalian, bacterial, or insect cells to express and purify specific isoenzymes (Friedberg et al., 1999; Parikh et al., 1997), as shown in Figure 3. Commercially available recombinant enzymes (e.g., CYP450s and UGTs) are widely used to study drug metabolism pathways, drug‒drug interactions, and the impact of genetic polymorphisms on biotransformation.

Recombinant CYP450 systems enable LC‒MS identification of drug metabolites, analysis of metabolic enzyme subtypes (e.g., kinetic profiling and enzyme specificity), and integration with liver microsome studies to pinpoint dominant metabolic pathways, accelerating drug interaction research and investigating substrate–enzyme binding for drug development. For example, Tang et al. (2011) successfully identified six metabolites of aconitine by using human liver microsomes and recombinant CYPs combined with liquid chromatography‒tandem mass spectrometry and clarified the leading role of CYP3A4/5 in aconitine metabolism. However, recombinant enzymes are expensive, and their activity is much greater than that of human microsomes (Li et al., 2010), which leads to differences between the experimental conditions of recombinant enzymes *in vitro* and *in vivo*, so the experimental results cannot be directly extrapolated to the human body.

### 4.2 Liver cell model

#### 4.2.1 Selection of hepatocytes

Hepatocytes are the main cells responsible for drug metabolism in the liver, so they are used to construct an *in vitro* model to simulate liver function. Commonly used hepatocytes, including primary human hepatocytes (PHHs), immortalized cell lines (such as HepaRG and HepG2), adult stem cells (such as liver progenitor cells and mesenchymal stem cells), human embryonic stem cells (hESCs) and human induced pluripotent stem cells (HIPSCs), have shown unique advantages and limitations as research tools. Although these cell types are selected on the basis of a series of ideal characteristics, their application is accompanied by shortcomings that cannot be ignored (Bale et al., 2016). Supplementary Table S1 supplements the application, advantages and disadvantages of cells, and provides a reference for cell selection.

#### 4.2.2 Stem cell culture

For *in vitro* studies of the liver, cell types that can reflect the characteristics of the liver *in vivo* should be selected, and appropriate culture methods should be selected according to the study duration. Two-dimensional (2D) culture is simple and suitable for short-term research, but long-term culture leads to functional decline (Schyschka et al., 2013). In contrast, three-dimensional (3D) culture, including sandwich culture, spheroid culture and bioprinting technology, can better simulate the liver microenvironment *in vivo* and maintain the long-term stability of cell function (Knobeloch et al., 2012; Schyschka et al., 2013; Messner et al., 2013; Bell et al., 2016; Ohkura et al., 2014; Gevaert et al., 2014; Du et al., 2008; Skardal et al., 2016). These methods can maintain the metabolism and transport function of heterogeneous organisms by affecting cell shape, cell‒cell contact and cell‒matrix interactions and are more suitable for complex research, such as long-term toxicity testing.

### 4.3 Cell models commonly used in the study of TCM *in vitro* liver metabolism

#### 4.3.1 Liver microsome model

Liver microparticles are rich in a mixed functional oxidase system that depends on cytochrome P450, which is very important for drug metabolism and biotransformation. Studying the metabolism of traditional Chinese medicine *in vitro* by using liver microsomes has multiple advantages. In the study of TCM, it is difficult to directly study the metabolism kinetics of TCM because of its complex components. The study of liver microsome metabolism *in vitro* can avoid interference *in vivo*, accurately detect the metabolic rate and characteristics of traditional Chinese medicine components, and predict drug properties and metabolic characteristics *in vivo* (Song et al., 2017).

Moreover, several obvious shortcomings are associated with liver microsomes. Because microsomes are only a part of the subcellular structure, they lack the synergistic effect of other key enzymes (such as NAT and ST) and coenzymes in the whole cell, so some metabolic reactions that require the participation of phase II enzymes cannot be carried out in this system. In addition, drugs and their metabolites easily contact drug-metabolizing enzymes in an *in vitro* liver microsome incubation system, which may not fully reflect the complex situation *in vivo*. More importantly, liver microsomes are rich in CYP450 and UGT enzymes. Compared with whole cells (such as hepatocytes and precision liver sections), they lack the competitive participation of other enzymes, which may lead to an abnormally high biotransformation rate. Therefore, when extrapolating the experimental results of a liver microsome incubation system to the human body, we must be extra cautious to avoid misleading drug research and development decisions (Li et al., 2011).

#### 4.3.2 Liver cytosol

**FIGURE 4:**
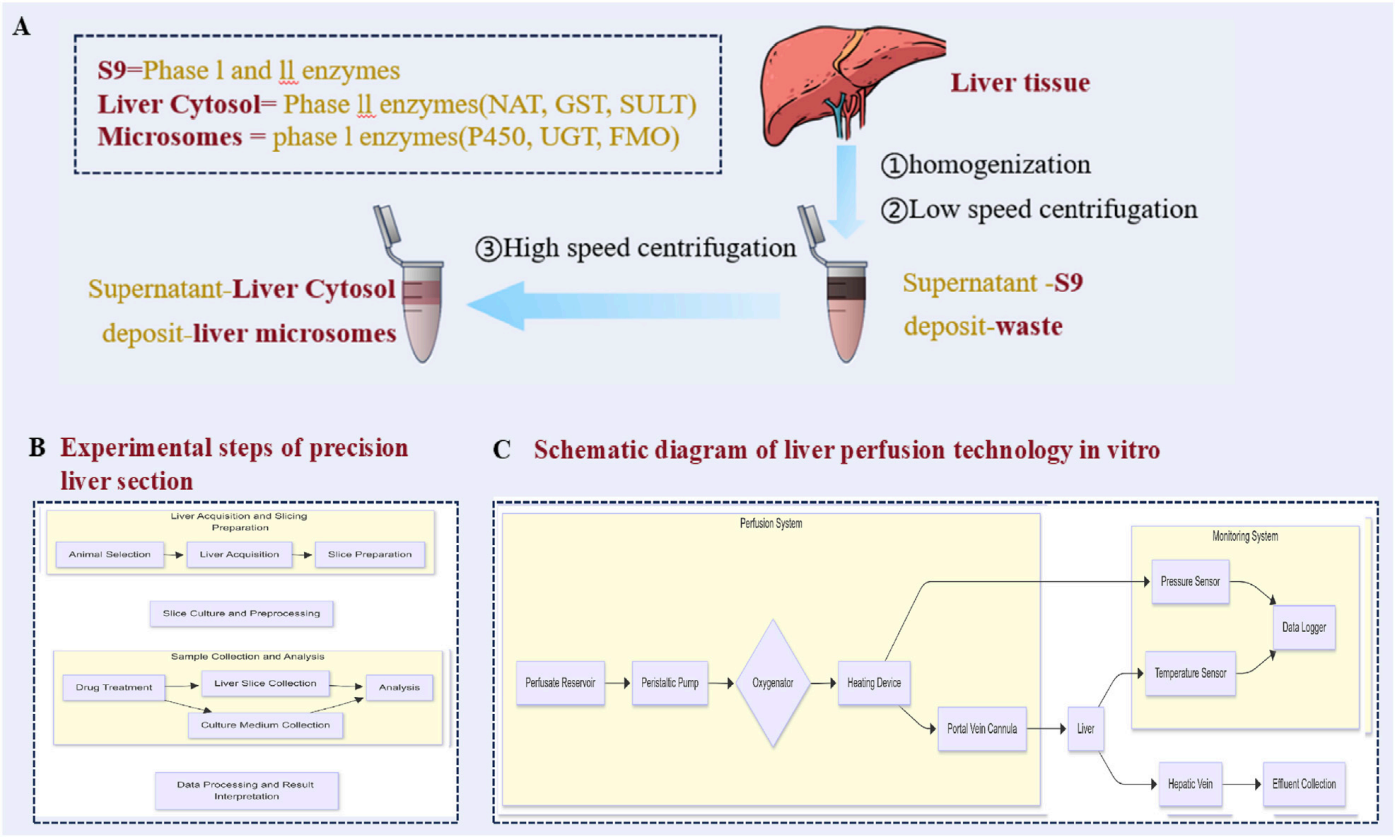
Metabolic model of acellular liver *in vitro*: **(A)** Preparation of S9, liver microsomes and hepatocyte homogeneity; **(B)** Flow chart of the experimental steps of precision liver sectioning; **(C)** Isolated liver perfection technology roadmap.

After ultracentrifugation, the supernatant of the liver cytosol was used to remove all organelles (Figure 4A), and the granules contained mainly phase II metabolic enzymes and fewer phase I metabolic enzymes. Compared with liver S9, the liver cytosol contains fewer phase I metabolic enzymes but more phase II metabolic enzymes, such as UDP-glucuronosyltransferase (UGT) and thiotransferase (SULT), which are usually used to study the activity of a single soluble enzyme and specific metabolic pathways (Zhang et al., 2008). Therefore, we can study a subenzyme independently by adding different coenzymes. Alternatively, these coenzymes can be added at the same time to study the activity of these enzymes as a whole. However, because the cytosol contains only soluble phase II metabolic enzymes, the system cannot be used to study the phase II metabolic enzyme UGT in the endoplasmic reticulum (Li et al., 2011).

#### 4.3.3 Liver S9

The suspension solution (S9) obtained by removing homogenate precipitate from liver tissue is a system with complete metabolic function and contains the components required for metabolism (Figure 4A). Its characteristic is that it contains complete phase I and phase II metabolic enzyme activities, which makes S9 provide more comprehensive information in the study of drug metabolism profiles than microsomes and cytosols do. Drugs that undergo phase I metabolism and then phase II metabolism to form specific metabolites are especially common. The S9 system can be more effective in qualitative research, and some metabolites that undergo phase I metabolism and then phase II metabolism can be found to form specific metabolites, which are often not easily found in the *in vitro* research system of microsomes or cytosols (Liu and Zhang, 2018). However, the S9 model also has some shortcomings. Because its metabolic enzyme activity is lower than that of liver microsomes and the cytosol, some metabolites are formed in small quantities and are difficult to detect. In addition, the S9 system contains many lipids and other proteins, which may reduce the free concentration of drugs in the system, thus affecting the accuracy of the experimental results. Although the concentration of membrane proteins (such as CYPs, UGTs and transporters) in S9 microsomes is lower than that in liver microsomes, the concentration of cytoplasmic or cytoskeleton-related proteins in S9 microsomes is greater than that in liver microsomes (Wang et al., 2020). This reflects the complexity and particularity of the S9 system to some extent.

#### 4.3.4 Precision liver sectioning

Precision liver sectioning (PCLS) is a model for *in vitro* metabolism research (Figure 4B) and has unique advantages because it retains the lobular structure and various cell systems. This versatile *ex vivo* model retains the multicellular histoarchitecture of the hepatic environment, including liver-infiltrating immune cells. Liver slices are also reproducible and inexpensive and maintain the viability of hepatocytes and Kupffer, endothelial, and hepatic stellate cells (Palma et al., 2019). It can be used to evaluate the influence of cell diversity and intercellular interactions on drug metabolism and can be used for drug transport experiments (Lake and Price et al., 2013). Moreover, because there is no need for protease digestion, PCLS is suitable for histological studies. However, this model also has several shortcomings, such as the drug having difficulty penetrating into the slice, which easily leads to necrosis of the central cells (Elferink et al., 2008). In addition, the preparation of PCLS requires fresh liver tissue, and peripheral cells are vulnerable. Although PCLS maintains the activity of most liver drug enzymes and organelles, its activity can last for only 5–7 days, which limits its application in long-term metabolic research. The slicing technology needs to be further improved to optimize the slicing thickness and culture conditions and prolong the viability maintenance time. Moreover, the preparation equipment of PCLS is expensive, which limits its Qualcomm screening application. Moreover, obtaining liver slices is technically challenging, and they are easily damaged, requiring seamless cooperation among multiple departments. Delays in the processing of surgical samples can lead to decreased viability, and it is difficult to obtain samples repeatedly for unplanned studies (Palma et al., 2019).

#### 4.3.5 Perfusion of isolated liver

*In vitro* liver perfusion is a model for *in vitro* studies using intact livers (Figure 4C), and the tissue structure and physiological function of the liver are maintained by artificial perfusion solution. This method effectively simulates the physiological environment of the liver *in vivo* and is suitable for studying drug metabolism pathways, first-pass effects and drug interactions (Zhang et al., 2012). It can qualitatively and quantitatively analyse the concentration changes of drugs and their metabolites (Fu et al., 2012). Maier-Salamon et al. (2008) used isolated liver perfusion studies in gene knockout rats to investigate the metabolism and biliary excretion of resveratrol. They demonstrated that resveratrol can be metabolized in the liver to form six glucuronide or sulfate conjugates. The glucuronide conjugates are excreted into the bile via Mrp2-mediated transport, whereas the sulfate conjugates are not excreted through the Mrp2 pathway. Gradolatto et al. (2004) investigated the hepatic metabolism of apigenin via *in vitro* hepatocyte suspensions and the isolated perfused rat liver technique. They reported that the IPRL model more closely reflects the *in vivo* metabolic processes of apigenin in the liver. However, isolated liver perfusion requires complicated intubation operations, and experimental conditions (such as perfusion fluid composition and flow rate) significantly affect liver function, with limited operation time and poor reproducibility, and it is difficult to perform multisample operations. Therefore, the model is mainly used in small-scale research, although it can truly reflect the metabolic changes in drugs at the whole-organ level.

#### 4.3.6 Liver bioreactor systems

A bioreactor is a container that provides the best requirements for the biochemical reactions of products (such as drugs, vaccines or antibodies) needed for industrial-scale synthesis and is used mainly to cultivate yeast, bacteria or animal cells (Mustafa et al., 2018). The model consists of a perfusion system, a detection system, an environmental control system, a nutrition supply system and other auxiliary systems. It is widely used in the study of drug metabolism and toxicity. The liver bioreactor can operate in recirculation and feeding mode, allowing repeated dosing tests to reflect the *in vivo* situation more accurately (Tostões et al., 2012). This approach is very useful for evaluating the effects of long-term drug exposure. Moreover, it can be used to culture many hepatocytes derived from stem cells, providing a sustainable cell source for drug testing. A bioreactor can accurately control culture parameters, provide a stable microenvironment, and help maintain the function and activity of hepatocytes (Farzaneh et al., 2020; Tostões et al., 2012). It is suitable for long-term culture of hepatocytes and can be used for long-term exposure to drugs (Tostões et al., 2012).

### 4.4 *In vitro* intestinal flora model

The interaction between the bioactive components of TCMs and the intestinal microflora has been the focus of many studies. When TCMs enter the digestive tract, the bioavailability of some components is limited due to the low intestinal absorption rate, and it is necessary to rely on the intestinal flora for metabolism or biotransformation to generate molecules with new biological activities to promote drug absorption and circulation. Therefore, the intestinal flora model plays a very important role in TCM metabolism research.

#### 4.4.1 Batch fermentation model

Batch fermentation (BFM) is the simplest, flexible and easy-to-operate method for studying the intestinal flora *in vitro;* this method involves the construction of a closed anaerobic environment for short-term simulation. Its principle is based on a static culture system; that is, flora inoculum (such as a fecal suspension or specific bacteria) and nutrient substrate are added at one time. The temperature was subsequently controlled by a constant-temperature heating plate, the pH was maintained by acid‒base titration, the anaerobic environment was ensured by nitrogen bubbling, basic chemical culture medium was provided to supply nutrition to simulate the intestinal ecological conditions, and the experiment was terminated when the substrate was exhausted and toxic metabolites (such as ammonia and organic acids) accumulated, destroying the initial flora balance (Nissen et al., 2020). Although the model has limited similarity with the real environment *in vivo* because it cannot simulate the dynamic material flow in the intestine and the host interaction, its advantages are low cost and flexible operation.

#### 4.4.2 Dynamic fermentation model

Dynamic fermentation models (DFMs) simulate the niches of different segments of the gastrointestinal tract (such as the proximal colon, transverse colon and distal colon) through multistage series reactors. A peristaltic pump is used to continuously supply a nutrient matrix to maintain the long-term activity of the flora, and the microaerobic/anaerobic environment of each reactor is accurately controlled by nitrogen/oxygen injection and a dissolved oxygen sensor (to avoid CO interference with pH) to restore the intestinal dynamics more accurately in time and space. The core of its principle is to control the first-stage reactor with sufficient acidic nutrition (simulating the rapid growth of proximal colon flora) and the subsequent reactor with neutral low nutrition (simulating the low-speed metabolism of the distal colon) by zoning and to maintain the activity of the fecal flora by combining it with basic medium at a constant temperature (Nissen et al., 2020; Gibson et al., 1988). Its advantages lie in remarkably improving the similarity *in vivo*, customizing and simulating the complex intestinal ecology, and being cost-controlled and operation-friendly, which provides a highly bionic platform for further studies of the interactions between flora and substrates.

#### 4.4.3 TNO *in vitro* model of the colon

The TNO *in vitro* model of the colon (Tim-2) is a patented system developed by the Dutch Applied Scientific Research Organization that simulates the proximal colon environment through a four-chamber glass reactor. Its core principle lies in multiparameter physiological simulation accurately controlled by a computer: periodic extrusion of a flexible membrane is used to achieve intestinal peristalsis mixing, warm water is circulated to maintain body temperature, and continuous nitrogen bubbling keeps the redox potential stable at −300 mV (close to the human colon level). At the same time, the semipermeable membrane dialysis system dynamically removes water and metabolic waste (to prevent toxic accumulation) and automatically injects NaOH to neutralize acidic metabolites to maintain pH 5.8 (Nissen et al., 2020). After inoculation with human fecal flora, the compound culture medium simulating the ileal effluent was continuously supplied with nutrients. After acclimation for 16 h, the experiment was carried out for 72 h, and the release of substrate, bioavailability and flora interactions were analysed in different regions (Meyer et al., 2004; Minekus, 2015). Although the model is time-consuming and costly, its advantage is that it is highly similar to the physiological environment *in vivo*: it can not only accurately quantify the material dynamics in different compartments but also has compatibility and high reproducibility in small laboratories, providing a reliable near-physiological platform for nutrition intervention and drug research.

### 4.5 Application of an *in vitro* metabolic model in TCM research

The *in vitro* metabolic model plays an increasingly important role in TCM research, especially in clarifying the metabolic transformation law and potential toxicity of complex components of TCMs. Recombinant enzyme technology, a recombinant cell line that can express specific CYP enzymes, is used to study the metabolism of TCM components mediated by specific enzymes. Mao and Wang (2023) used this model to prove that CYP1A2 is the main P450 enzyme subtype involved in the metabolic activation of chelerythrine chloride. Liver microsomes, as a classical model, are often used to study the phase I metabolism of TCM components. For example, He et al. (2022) used liver microsomes to investigate the effect of curcumin on cytochrome P450 2C8 activity in rat and human liver microsomes. Liu J et al. (2022) studied the metabolic stability and metabolic enzyme phenotype of Chebulinic acid in different species of liver microsomes. However, owing to the lack of phase II metabolic enzymes, it is difficult for the liver microsome model to fully reflect the metabolic process *in vivo*. The liver cytosol model is rich in phase II metabolic enzymes, which can simulate liver metabolism more completely when combined with liver microsomes. The S9 component model contains both I-phase and II-phase metabolic enzymes, which more comprehensively reflect the metabolic process of traditional Chinese medicine components *in vivo*. For example, Peeters et al. (2020) identified the metabolites of medicagenic acid in S9, human liver microparticles (HLMs) and the liver cytosol (HLCYT). When the metabolites in S9 were compared with those after HLM/HLCYT coincubation, no significant difference was found in the types of metabolites. To better simulate the physiological environment of the liver, an *in vitro* metabolism model based on hepatocytes, including primary hepatocytes, immortalized hepatocytes and hepatocytes derived from iPSCs, is commonly used in the identification of metabolic phenotypes, metabolic pathway analysis and drug interaction research on TCM components. For example, Wang et al. (2024a), Wang et al. (2024b) studied the hepatotoxic mechanism of rhein by using a primary hepatocyte model. In addition, liver perfusion technology and liver bioreactors can maintain the physiological structure and function of the liver for a longer time, which is more suitable for long-term metabolism and toxicity research. Liver chip technology integrates many cell types and microfluidic technology, which can better simulate the complex microenvironment of the liver and provide a new direction for TCM metabolism research.

Compared with that of Western medicine, which mainly depends on the metabolism of liver drug enzymes, the metabolism of TCM is more characterized by a liver‒intestine dual path. The intestinal flora can significantly alter the chemical properties of TCM components through metabolic processes. For example, the intestinal flora, such as *Escherichia coli*, can readily metabolize baicalin into baicalein (Kim et al., 2008). Baicalein is efficiently absorbed from the intestine into the body, where it can then be converted back into baicalin. Ginsenoside Rg1 can be metabolized by human and rat intestinal bacteria, and its metabolites persist as the main active components in blood and constitute effective bioactive components in the human body (Chen, 2011). The specific anaerobic bacteria in the human intestine can transform the compound glycyrrhizin or one of its intermediate metabolites into glycyrrhizic acid and then enter the blood through the liver and intestinal circulation (Xi et al., 2008; Zhao et al., 2007). These compounds significantly improved in bioavailability and efficacy after intestinal flora transformation *in vitro*. In addition, the *in vitro* study of the intestinal flora has a mechanism that is homologous to that of traditional Chinese medicine processing methods, fermentation processing (probiotics/two-way fermentation), which provides an experimental basis for revealing the mechanism of increased drug effects after processing. The transformation process also has a two-way regulatory function: toxicity can be reduced after the transformation of the intestinal flora, such as the metabolism of aconitine to hypoaconitine, which has low toxicity (Zhang et al., 2022). It may also produce toxic substances, such as nitrides, to produce cyanide (Zhang et al., 2022). Further study revealed that an *in vitro* flora model can be used to analyse the compatibility law of traditional Chinese medicine in detail. Research by Liang et al. (Xi et al., 2008) revealed that the intestinal flora of rats could effectively degrade tetrahydropalmatine, and its content decreased with increasing incubation time. Coumarin (a synergistic component of *Corydalis yanhusuo*) significantly delayed metabolism, which revealed the scientific compatibility of *Corydalis yanhusuo* from the perspective of intestinal flora metabolism. Supplementary Table S1 compares the characteristics of metabolic models.

## 5 *In vitro* excretion model

The main elimination routes of drugs are the kidney and the hepatobiliary system, and the excretion ability of bile can be studied via bile membrane vesicles. In recent years, various *in vitro* culture models, such as tissue culture, primary culture of renal cells and slice culture, have been used to study renal excretion, toxicity and the mechanism of drugs. Among them, all kinds of immortalized cells have become reliable cell models for studying drug excretion and nephrotoxicity.

Among the commonly used *in vitro* models, the isolated kidney perfusion (IPK) model, liver cell sandwich culture model and various kidney cell models are important. Compared with other *in vitro* models, immortalized cells are more widely used in TCM research. Among them, the commonly used renal cell model covers a variety of cell lines, such as the MDCK cell line, which represents renal distal convoluted tubules; the OK cell line, which simulates renal proximal convoluted tubules; the LLC-PK1 cell line; and the HK-2 cell line.

### 5.1 Kidney cell model

In the study of drug metabolism, the kidney cell model, as an important tool (Supplementary Table S2), plays a very important role in the study of drug metabolism, excretion and toxicity mechanisms in the kidney. Freshly isolated proximal renal tubular cells (PRPTCs) are often used to study the mechanism of active drug secretion and reabsorption. Because they retain the expression and physiological functions of transporters (such as organic anion/cation transporters) *in vivo*, their easy dedifferentiation *in vitro* limits their long-term application (Adler et al., 2016; Lechner et al., 2021). Immortal cell lines (such as MDCKII and HK-2) are widely used for drug transmembrane transport and metabolic screening because of their stability and Qualcomm capacity, but insufficient expression of transporters/metabolic enzymes may reduce the correlation *in vivo* (Lechner et al., 2021). In addition, exogenous cell lines (such as HK-2 and RPTEC/TERT1) can partially simulate the function of human renal tubules in some cases. However, the expression of transporters and tight junction proteins in these cell lines is usually different from that *in vivo* (Jenkinson et al., 2012; Kim et al., 2002; Wieser et al., 2008). Animal-derived cells (such as LLC-PK1 and OK) are suitable for mechanistic exploration, but they are not recommended for excretion or toxicity prediction owing to species differences (Chi et al., 2021; Lechner et al., 2021). In view of the diversity of renal cell types, choosing a suitable cell type for successful studies is very important.

### 5.2 Isolated perfused kidney

An isolated perfused kidney (IPK) separates the kidney from the living body. The perfusion of “blood” *in vitro* is a method to study the kidney specifically without being affected by the whole-body blood circulation system and the regulation of neurohumors. Initially, the model was mostly used in the study of kidney physiology and biochemistry, and it is now widely used in the study of drug excretion, metabolism, clearance, drug interactions and drug nephrotoxicity in the kidney. The obvious advantage of IPK technology is that it can effectively eliminate the interference of many complex factors in the body while maintaining the complete function of the kidney, thus providing a purer and more controllable research platform. Researchers can accurately control the protein concentration, pH value and other parameters of perfusion fluid and then systematically explore the specific effects of different factors on renal function. However, this technology also has several limitations. For example, the flow rate of perfusion fluid is relatively high, which may cause the kidney to bear a certain pressure; at the same time, the function of distal renal tubules may be weakened *in vitro*. In addition, mechanical injury or hypoxic injury may inevitably be introduced during *in vitro* operation, which may adversely affect the normal function of the kidney and the accuracy of the experimental results (Taft, 2004; Nizet, 1975; Georgiev et al., 2011).

### 5.3 Liver cell sandwich culture model

Hepatocyte sandwich culture involves the inoculation of freshly separated or frozen primary hepatocytes between two substrates (collagen in the lower layer and artificial basement membrane in the upper layer) for culture (Gijbels et al., 2019). Primary hepatocytes cultured in this model for 3∼5 days can form bile duct-like structures between hepatocytes, and the expression of transporters and metabolic enzymes can maintain a high level, which can better simulate the *in vivo* environment and is the main model for studying the function of liver transporters *in vitro* (Liang, Song, Ge, ZHang, Dai, et al., 2018). Liu et al. (2019) demonstrated that tanshinone IIA can reverse the inhibitory effect of rifampicin on the transport capacity of the bile acid efflux pump (BSEP) through a sandwich culture model of rat hepatocytes. Therefore, tanshinone IIA has the potential to treat cholestasis caused by rifampicin.

### 5.4 Application of the *in vitro* excretion model in TCM

The *in vitro* excretion model is playing an increasingly important role in traditional Chinese medicine research, especially in evaluating the excretion pathway, excretion kinetics and potential nephrotoxicity of multiple components of traditional Chinese medicine. Renal cell models, such as primary renal tubular epithelial cells and immortalized renal tubular epithelial cell lines, are often used to study the transport, metabolism and excretion characteristics of traditional Chinese medicine components in the kidney. For example, Li B et al. (2019) and Li Y et al. (2019) studied the effects of tongfenging serum containing urate transporters on the function of urate transporters in a human renal tubular epithelial cell line (HK-2) induced by uric acid. However, fully simulating the complex physiological environment of the kidney with a single renal cell model is difficult. In an *in vitro* renal perfusion model, because the complete structure and some physiological functions of the kidney can be retained, the renal excretion process and potential nephrotoxicity of traditional Chinese medicine components can be evaluated more comprehensively. As Zou et al. (2021) used isolated rat kidney perfusion technology to study the mechanism of cichoric acid in reducing uric acid, the operation of this model was complicated, and the cost was high. Although the liver cell sandwich culture model is mainly used to study liver metabolism, it also plays an important role in evaluating the characteristics of bile excretion of traditional Chinese medicine components, which can be used to study the process of liver uptake, metabolism and bile excretion of traditional Chinese medicine components and explore drug interactions. Liang et al. (2018) used this model to compare the bile excretion characteristics of berberine, palmatine and jateorhizine.

## 6 *In vitro* toxicity evaluation

**FIGURE 5:**
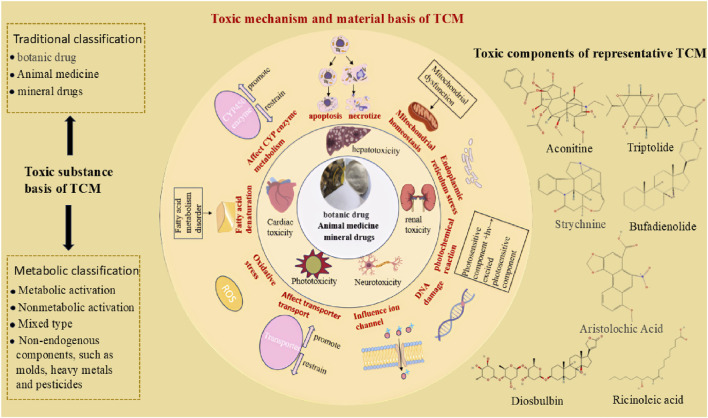
The general mechanism and material basis of the toxic effects of TCMs (Toxic substance basis of TCM; mechanism of universal toxicity of TCM; common toxic substances of traditional Chinese medicine).

TCM is rich in medicinal resources and has a wide range of applications in the medical field. However, owing to the complexity of its components and the interactions between the components, the toxic mechanism of most of its components is not completely clear, and related research is in the stage of continuous exploration and accumulation of experience at home and abroad. In view of the urgent need for TCM modernization development. Accurate qualitative and quantitative analyses of TCMs containing toxic components have been carried out to reveal the specific mechanism of their toxicity. It is increasingly critical to construct a set of experimental data and safety evaluation models that can objectively evaluate the toxicity level of TCMs. In this process, the *in vitro* model is widely used in studies of the toxicity of traditional Chinese medicine because of its advantages of low cost and Qualcomm screening. It can not only simulate the internal environment of the human body but also provide a platform for accurate evaluation of the toxicity of TCMs, reveal the mechanism of action of the toxic components of TCMs, and provide a scientific basis for the safe use of TCMs. The common toxic substances and general toxicity mechanisms of TCM are shown in Figure 5.

### 6.1 *In vitro* studies of the toxicity of TCMs in cytotoxicity experiments

In studies of TCM toxicity, because of the diversity, complexity and fuzziness of TCM components, the efficacy and toxicity of TCM in the body show an overall comprehensive effect (Peng et al., 2008). Unlike a single chemical component, when TCM extract is directly added to an *in vitro* reaction system, it may cause changes in the cell osmotic pressure and pH value due to the presence of many impurities (such as tannins and inorganic salts). This may seriously interfere with the experimental results and lead to false positive or false negative results. Therefore, when identifying toxic substances in traditional Chinese medicine, there is often great controversy, which makes them difficult for the scientific community to recognize widely (Chen et al., 2017b).

Professor Masakazu Tashiro, a Japanese scholar, proposed the concept of “drug serum” in the 1980s. After oral administration to animals, the serum was taken as a drug source and added to the *in vitro* reaction system (Yuzhuo, 2006). This method effectively overcomes the interference between the physical properties of TCM crude extracts and impurities, and the experimental conditions are closer to the internal environment in which drugs produce effects *in vivo*. On the basis of this development, the “serum pharmacology of traditional Chinese medicine” has become a commonly used experimental method of TCM pharmacology, which provides an effective way to study TCM, especially the pharmacological effects of TCM compounds (Chen et al., 2017b). Researchers tend to use drug-containing serum for drug administration when conducting cell toxicity experiments *in vitro*, and the use of medicated serum can more accurately reflect the actual effects of drugs in the body, thus improving the accuracy and reliability of the experiment.

### 6.2 Common cell types for *in vitro* cytotoxicity evaluation

Cytotoxicity evaluation involves selecting cell types, establishing evaluation indices and selecting appropriate detection methods. In accordance with the characteristics of the test object, liver tissue cells are selected for evaluating liver toxicity, with a focus on subcellular indicators, such as mitochondrial toxicity, according to the type of toxicity. Different indices correspond to different detection methods; for example, changes in the mitochondrial membrane potential can be detected by flow cytometry (Sun et al., 2011). The cells commonly used in *in vitro* cytotoxicity evaluation are mainly cells obtained directly from human tissues or from model organisms closely related to humans.

Cells derived from normal tissues are commonly used in *in vitro* cytotoxicity evaluations. Their functional and structural characteristics make *in vitro* cytotoxicity tests more targeted and specific, facilitating the elucidation of the mechanisms by which toxic substances cause cellular damage (Liu G et al., 2014). Lian et al. (2021) investigated the hepatotoxicity mechanism of coptisine in a normal human liver cell line (L02) and reported that coptisine can induce apoptosis in L02 cells via both the mitochondrial and endoplasmic reticulum stress (ERS) pathways. Bao et al. (2020) reported that *Polygonum multiflorum* ethanol extract (PME) increased ROS levels and LDH release rates in L02 cells while decreasing SOD activity and the mitochondrial membrane potential (MMP). Subsequent Western blot results further demonstrated that PME may induce hepatocyte apoptosis through the ROS-mediated mitochondrial pathway. Cells derived from tumor tissues represent another commonly used cell type in *in vitro* cytotoxicity evaluation. These cells can proliferate indefinitely under *in vitro* culture conditions, which has made them widely used in such studies (Liu G, et al., 2014). Liu C et al. (2014) and Liu T et al. (2014) reported that jervine induces apoptosis in HepG2 cells by damaging the cell membrane and mitochondria and activating apoptosis-related genes. For example, Liu et al. (2016) studied the mechanism of the nephrotoxicity of aristolochic acid in an HK-2 cell model and screened 11 potential nephrotoxicity biomarkers by analysing the time course of the metabolic changes in aristolochic acid in HK-2 cells. The commonly used cell types are shown in Supplementary Table S3.

### 6.3 Special toxicity applications

#### 6.3.1 Phototoxicity

Phototoxicity is a harmful reaction of some compounds after exposure to light. Some components of TCM, such as flavonoids (quercetin, rutin), coumarins (furocoumarin), alkaloids (berberine), curcumin derivatives and other components (such as chamazulene), have been shown to be phototoxic (Li et al., 2018). In the past, the evaluation of phototoxicity has relied mainly on basic *in vitro* screening tests, including photohemolysis tests, lipid photoperoxidation tests and protein photodamage tests. Although these tests are easy to perform and can provide some basis for preliminary safety evaluation, their prediction accuracy is limited and may not be enough to accurately evaluate the risk of phototoxicity (Khattak et al., 2012). With increasing research, more effective methods for evaluating phototoxicity have been developed. At present, the 3T3 NRU-PT test is one of the most widely used phototoxicity detection methods and is famous for its high sensitivity, ability to predict negative results effectively, and ability to provide sufficient evidence that substances are not phototoxic. In this case, there is usually no need for further testing, and it is considered that there will be no direct phototoxicity in humans. As an *in vitro* method for animal skin phototoxicity testing, 3T3 NRU-PT has the advantages of simple operation, low cost, good reproducibility and a short test period and has good correlation with *in vivo* experimental results (Seto et al., 2015). In addition to 3T3 NRU-PT, the *in vitro* detection system also includes the developed and verified UVA simulator reactive oxygen species (ROS) test as an alternative method for phototoxicity assessment. However, this method may overestimate the phototoxicity of negative substances (Lee et al., 2017). In addition, photodissociation tests based on capillary electrophoresis (CE) and *in vivo* biological phototoxicity tests (IBPs) combined with photochemical, photobiological and pharmacokinetic data have also been proposed as new screening strategies to predict the risk of phototoxicity *in vivo* (Seto et al., 2011).

#### 6.3.2 Neurotoxicity

Neurotoxicity is a common adverse effect in drug development, and early screening is crucial for drug development. The existing *in vivo* experimental methods have limitations such as high subjectivity, long cycles, and high costs. In comparison, in neurotoxicity screening, *in vitro* human cell models have the advantages of high throughput, low cost, and cross-species applicability and are an effective alternative to *in vivo* experiments (Tian et al., 2020). In neurotoxicity research, *in vitro* models can be used to explore mechanisms, simulate neural developmental stages, and are suitable for assessing the safety of traditional Chinese medicines. Neuronal cell lines such as neuroblastoma cells (SH-SY5Y) and adrenal pheochromocytoma cells (PC12) are widely used in research on neurological disorders such as Parkinson’s disease and cerebral ischemia (Xicoy et al., 2017; Lahiani et al., 2018; Zhang et al., 2019). In addition, neural stem cells (NSCs) have become an ideal choice for developmental neurotoxicity tests because they can differentiate into various nerve cell types (Hong et al., 2019). Three-dimensional cell culture technology enhances *in vitro* model simulation capabilities and improves toxicity prediction sensitivity. The combination of multiple models enables comprehensive analysis of drug neurotoxicity. However, there are currently no *in vitro* guidelines for assessing the risk of neurotoxicity (Hartung et al., 2003). However, an *in vitro* model of neurotoxicity has been used in basic research for many years in the future, and the development of a new *in vitro* model for the comprehensive evaluation of nervous system function may become an important research direction in the future.

### 6.4 Present situation and future prospects of TCM *in vitro* toxicity evaluation

There are fundamental differences between TCM and Western medicine in toxicity research. Western medicine relies on the well-defined physicochemical properties and targeted mechanisms of single compounds, allowing toxicological studies to focus on precise dose‒effect relationships and specific targets. In contrast, TCM is a complex multicomponent system whose toxicity mechanisms are dynamically influenced by factors such as the diversity of biological sources, products of processing, interactions among combined ingredients, and *in vivo* biotransformation. Adverse reactions are often the result of synergistic effects between toxic and nontoxic components. Traditional empirical knowledge makes it difficult to quantify toxicity thresholds, leading to several challenges in TCM toxicity research: mechanistic ambiguity (multitarget effects complicate toxicological mechanism elucidation), unclear material basis (lack of systematic characterization of component transformations and novel toxic substances arising from processing and formulation), rough dosing (variability in bioactivity makes it difficult to define safety windows), and reliance on experience (individualized treatments hinder standardized evaluation). The advent of “precision medicine” has further challenged TCM toxicity research. Improving the safety of TCM and preventing safety incidents such as those related to TCM injections have become important tasks for research in TCM toxicology.

Under this background, *in vitro* models have been widely used in toxicity studies of TCMs because of their low cost, simple operation and Qualcomm screening ability. By using drug serum technology and optimizing the experimental conditions, the nonspecific interference of the TCM crude extract *in vitro* was effectively overcome, and the accuracy of the experimental results was significantly improved. Moreover, *in vitro* cytotoxicity evaluation has established a variety of cell models from humans or model organisms, covering many key organs, such as the liver, kidney, heart and nerve, which provides strong support for accurate evaluation of TCM toxicity. In addition, the introduction of organ chip and organ-like technology further compensates for the shortcomings of traditional cell models and animal experiments and provides innovative solutions for studying the toxicity of traditional Chinese medicine compounds. However, *in vitro* studies of the toxicity of traditional Chinese medicine still face challenges in terms of technology and standardization, and more sensitive and specific models and methods need to be further developed. In the future, with the development of 3D bioprinting, microfluidic technology and Qualcomm screening platforms, research on the toxicity of traditional Chinese medicine is expected to achieve more efficient and accurate evaluations and provide a more reliable scientific basis for the safe use of TCM and the development of new drugs.

## 7 Organoids

**FIGURE 6:**
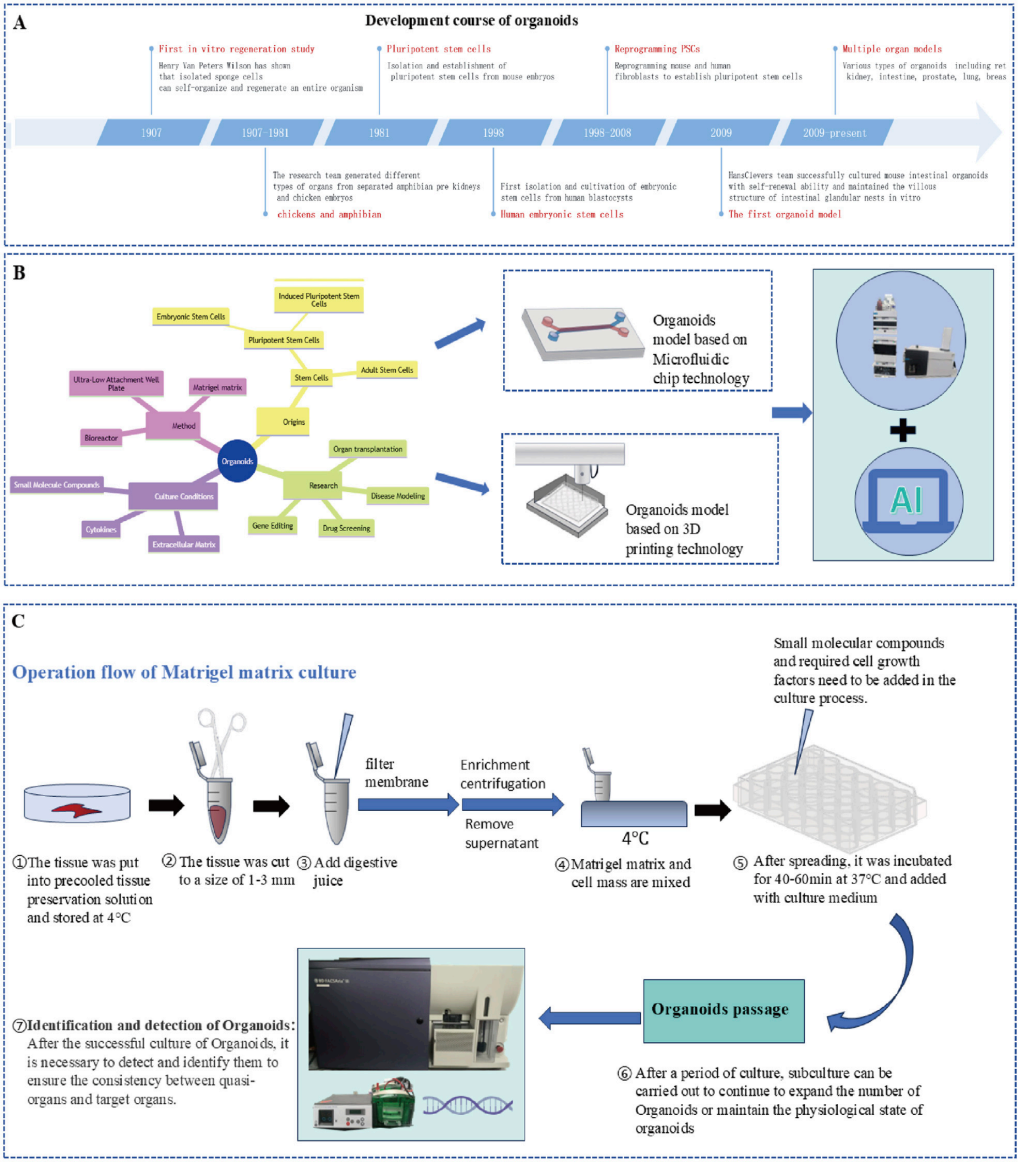
**(A)** Development timeline of organoid technology (1907-present); **(B)** Organoid construction technology system: stem cell source (PSCs-ESCs/IPCS, ASCS); culture method (ultralow adsorption culture plate and bioreactor, matrix gel method); culture conditions (small molecular compounds-promoters/inhibitors, cytokines, matrix glue); combination of new technologies (microfluidic chip, biological 3D printing technology); and technology convergence (LC‒MS/MS, Ai). **(C)** Process of organ culture with a Matrigel matrix.

Organoid technology was developed a hundred years ago, from H.V. Wilson’s discovery that sponge cells can self-organize to form new organisms in 1907 to Hans Clevers’s initiative to reconstruct the crypt–villus structure of intestinal organoids in 2009 (Schutgens and Clevers, 2020). These findings indicate that this technology has entered a new stage of functional modelling, as Figure 6A. At present, organoids have developed into a whole-chain research system covering disease mechanism analysis, Qualcomm drug screening and regenerative medicine treatment. The construction of normal organoids mainly depends on two types of stem cells: pluripotent stem cells (PSCs) (including embryonic stem cells (ESCs), induced pluripotent stem cells (IPCSs) and adult stem cells (ASCs)) (Schutgens and Clevers, 2020). By optimizing the composition of the culture medium and precisely regulating signalling pathways, researchers drive stem cells to differentiate into target tissues. The core components of the classical WENR (or WNER) culture scheme include Wnt-3a, EGF, Noggin and R-spondins (Schutgens and Clevers, 2020). Through ingenious combination with other cytokines or small molecule inhibitors, WENR-derived culture conditions are suitable for the construction of various organoids. At present, the WENR protocol has been widely used in the culture of many organoids, such as those from the stomach, small intestine, colon, pancreas and liver. Figure 6C shows the process of organoid culture using Matrigel matrix. In recent years, microfluidic chips and biological 3D printing technology have injected new kinetic energy into organ-like research. A microfluidic system can dynamically simulate the physiological dynamic environment by accurately controlling the fluid shear force and oxygen gradient, and at the same time, it can be used to construct an organoid interaction model through chip technology (Wang et al., 2024a). Biological 3D printing technology can accurately reconstruct the vascular network and tissue structure in the spatial dimension and overcome the size and functional limitations of traditional organoids (Garreta et al., 2021). With the rise of artificial intelligence technology, the research process has accelerated, and the objectivity and efficiency of research have been significantly improved by efficiently analysing the images, omics and drug screening data of organoids through artificial intelligence (Wu et al., 2024).

### 7.1 Intestinal organoids (IOs)

*In vitro* models previously used to study intestinal nutrient absorption, drug transport and intestinal cell metabolism, such as Caco-2 cells or rodent explant models, have limited value because they are derived from cancer and nonhuman sources, respectively. In particular, species differences lead to poor correlation data, which leads to insufficient reliability of drug cross-species extrapolation (Zietek et al., 2020). This lack of physiological correlation has become the key reason for the high failure rate of ADME prediction in new drug development. However, IOs can retain the expression characteristics of specific functional genes and transporters (such as GATA4, NHE3, and ENaC) in their original intestinal segment and accurately reflect the physiological relevance of primary tissues (Middendorp et al., 2014). More importantly, this technology can be used to construct a disease-specific drug absorption model. Sarvestani et al. (2021) first constructed an organoid model of ulcerative colitis (UC) on the basis of patient-derived iPSCs. Nishimura et al. (2019) constructed a pathological model of ulcerative colitis (UC) *in vitro* on the basis of human colon organoids and successfully revealed the drug response of intestinal epithelial cells under inflammatory stress. Elbadawy et al. (2021) successfully analysed amorphous curcumin by IOs and achieved targeted clearance of CSCs and sensitization to chemotherapy by increasing the bioavailability of anticancer drugs. These breakthroughs indicate that the organ-like model has advanced from a simple absorption prediction tool to a precise experimental system for analysing pathology–pharmacokinetics.

### 7.2 Brain organoids (BOs)

In 2013, Lancaster et al. (2013) published a study on the cultivation of complex classical human brain organs, which simulated some structural and functional characteristics of the brain, marking the official birth of BO technology. In 2017, Xiang et al. (2017) fused BOs of different brain regions to obtain more complex BO types. Birey et al. (2017) subsequently fused thalamic organoids with cortical organoids to simulate the bidirectional projection of neurons between the thalamic cortex. The development of BOs has achieved a leap-forward breakthrough from infrastructure simulation to complex function reconstruction.

The application of BOs in drug research has the unique advantages of three-dimensional spatial analysis and physiological microenvironment simulation. Park et al. (2021) used an iPSC-induced BO model of patients with Alzheimer’s disease (AD) to construct a Qualcomm dose-based drug screening system. To identify an anti-AD candidate drug library approved by the FDA that can penetrate the blood‒brain barrier. This further proves its potential in drug screening. BOs can be combined with mass spectrometry imaging technology. By freezing cultured BOs and scanning slices with uniform structures via mass spectrometry, we can determine the spatial distributions of different molecules (such as lipids, sugars, amino acids, drugs and their metabolites) in organs (Khalil et al., 2024; Cappuccio et al., 2023). This technology can further clarify the dynamic distribution mechanism of drugs.

### 7.3 Metabolic and excretory organoid model

In the study of drug metabolism and excretion, organoids have become an important tool for analysing the mechanism of drug biotransformation because of their bionic metabolic enzyme activity and tissue-specific microenvironment. For example, compared with traditional hepatocyte models, liver organoids (LOs) can perform more complex liver functions, such as drug metabolism, detoxification, protein synthesis and secretion. Park E et al. (2019) constructed mouse stem cell-derived LOs and systematically analysed the mechanism of drug metabolism mediated by the CYP enzyme. First, the expression of members of the CYP subfamily (such as CYP3A) is significantly increased by a CYP inducer, and then, the drug decomposition ability of organs is improved, as shown by docetaxel metabolism experiments. Furthermore, a liver‒pancreas organoid‒tumor coculture system was established, and it was found that the differentiated liver organs induced by CYPs could metabolize docetaxel efficiently (the survival rate of tumor cells in the coculture group was 101.90 0.94%, which was significantly greater than that in the noninduced group (66.05 2.14%). The biological process of liver metabolism detoxification is completely reproduced, which provides an inducible and quantitative functional platform for *in vitro* evaluation of drug metabolism-dependent toxicity and individualized drug use research.

Kidney organoids (KOs) contain many cell types, and the effects of drugs on kidneys often depend on many cells; thus, kidney organs can better match the effects of drugs in the body (Chen et al., 2021). Various structural units, such as glomerular podocytes, proximal tubules, distal tubules and collecting tubules, similar to those of the human kidney, can be formed via organoid technology.

Biliary epithelial cells (bile duct cells) protect the liver, regulate bile components and transport substances, and bile duct organoids can simulate these functions well and play an important role in the study of bile excretion. Mizoi et al. (2024) constructed a monolayer model of bile duct organoids from primary human hepatocytes and simulated the polar structure of the bile duct epithelium (a side of the apical membrane/B side of the basal side). The bile duct marker CK19 and bile excretion transporters (P-gp, MRP2, and BCRP) are highly expressed in organoid bodies, and their polarity is located on the A side. The transport experiment revealed that the permeation rate of drugs from the B→A side is significantly greater than that from the reverse side, and this directionality can be eliminated by transporter inhibitors, which confirms the mechanism of drug-directed excretion mediated by apical membrane transporters and provides a bionic research platform for the quantitative analysis of drug bile excretion.

### 7.4 Application of organoids in toxicity prediction

Organoid technology provides innovative solutions in drug toxicity research, which compensates for the shortcomings of traditional *in vitro* cell models and animal models. Organoids can better reflect the function of human tissues and have been widely used to evaluate the hepatorenal toxicity caused by drugs. Morizane et al. (2015) used renal organoids derived from human embryonic stem cells (hESCs) to evaluate the potential nephrotoxicity of drugs in detail, and the results revealed damage to the proximal and distal renal tubules under drug exposure. Mun et al. (2019) successfully constructed liver organoids derived from hiPSCs and evaluated the hepatotoxicity of four drugs that can cause liver injury according to the detection of the cell survival rate, ROS level, glutathione content and mitochondrial respiration. Shinozawa et al. (2021) used human liver organoids to screen the hepatotoxicity of 238 drugs in the form of Qualcomm quantities. In addition, organoid models have been used to study drug-induced cardiotoxicity (Chen et al., 2023), enterotoxicity (Crespo et al., 2018), reproductive toxicity (Li et al., 2021), retinal toxicity (Eade et al., 2021) and neurotoxicity (Scholz et al., 2022). In the field of drug toxicity screening, owing to technical limitations and high cost, it is difficult to construct 3D organoid models for large-scale drug toxicity screening in batches. Therefore, it is necessary to further develop 3D organoid construction technology (for example, with the help of microfluidics and 3D bioprinting) and use Qualcomm screening technology to establish a faster, more efficient and more accurate drug screening platform.

### 7.5 Application status of organoids in TCM research

In August 2022, the world’s first new drug, sutimlimab, whose preclinical data were completely from organ-like technology, was approved by the Food and Drug Administration (FDA) to enter clinical trials (Rumsey et al., 2022). At the end of December of the same year, the FDA Modernization Act 2.0, approved by the U.S. House of Representatives, announced that it was no longer mandatory to conduct animal tests before clinical trials of drugs, which indicated that the technology had moved from basic research to industrial application (Wadman, 2023). In the field of TCM research, organoids also present unique value. Among them, IOs provide a dynamic research platform for revealing the absorption mechanism of traditional Chinese medicine components. Glycyrrhetinic acid (GA) is among the main components of *Glycyrrhiza uralensis Fisch*. dinate and promote the effects of other drugs in traditional prescriptions. Chen et al. (2019), using small intestinal organoid models developed from intestinal crypt stem cells, demonstrated that GA increases the levels of human antigen R (HuR) and its downstream protein Ki67, thereby promoting intestinal organoid development. Combined with the *in vivo* experimental data, these findings revealed that GA facilitates intestinal epithelial homeostasis through HuR regulation. These experimental results indicate that *Glycyrrhiza uralensis Fisch*. can increase the absorption of drug components in the small intestine, providing modern scientific evidence for the traditional role of *Glycyrrhiza uralensis Fisch*. in “harmonizing and coordinating medicinal effects” in classical herbal formulations.

In the study of drug distribution, compared with the Transwell model of coculture, the BBB organoid model expresses more P-gp and ZO-1, which can simulate the characteristics of the BBB more accurately *in vivo* (Smyth et al., 2018). Du et al. (2022) constructed organoids with key characteristics of the blood‒brain barrier (BBB) by integrating human brain microvascular endothelial cells (HBMECs), human brain astrocytes (HAs) and human cerebrovascular pericytes (HBVPs). With this model system, researchers have systematically evaluated the effect of Guanxi injection (GXNI) on BBB dysfunction induced by oxygen‒glucose deprivation/reoxygenation (OGD/R). OGD/R injury can significantly reduce the expression level of P-glycoprotein (P-gp) in an organ-like model, and the downregulation of this transporter may promote some active components that cannot penetrate the BBB to enter the central nervous system to play a therapeutic role by regulating the drug efflux mechanism. Further research revealed that GXNI not only effectively alleviated the decrease in organoid viability caused by OGD/R but also significantly inhibited the abnormal increase in barrier permeability in the model group, which provides new *in vitro* research evidence that explains the mechanism by which GXNI protects the brain by regulating the dynamic balance of the BBB.

Organoids improve TCM safety evaluation through multidimensional toxicity prediction. Gu et al. (2023) revealed for the first time that esculentoside A, the main active component of Radix Phytolaccae, induced mitochondrial damage, promoted the release of mtDNA into the cytoplasm, activated the STING signalling pathway, and then triggered an inflammatory cascade and interstitial transformation of renal endothelial cells, eventually leading to renal tubular polarity loss and functional damage. Qian et al. (2023) constructed liver organoids (LOs) via a thermoresponsive alginate-RGD/Pluronic hydrogel, innovatively integrating a fluorescent sensing system based on the hybridization chain reaction (HCR) amplification strategy. By dynamically monitoring the secretion of glutathione S-transferase alpha (GST-α), an early biomarker of hepatotoxicity, they achieved precise *in vitro* evaluation of the hepatotoxicity of natural compounds such as emodin and triptolide. This work established a novel methodological framework for real-time tracking of dynamic biomarkers, advancing toxicity research in TCM.

In TCM research, organoids are used primarily for pharmacological efficacy and toxicity studies, with fewer cases focused on ADME aspects. However, in human ADMET processes, the construction of organoid models for critical organs has already been well established. Furthermore, with advancements in chip technology, breakthroughs in microfluidic organ-on-a-chip systems are progressively addressing the gaps in ADMET research for traditional Chinese medicine. The application of organoid technology in the evaluation of TCM toxicity is summarized in Supplementary Table S4.

## 8 Organ-on-chip (OoC)

### 8.1 OoC technology based on microcontrol flow

**FIGURE 7:**
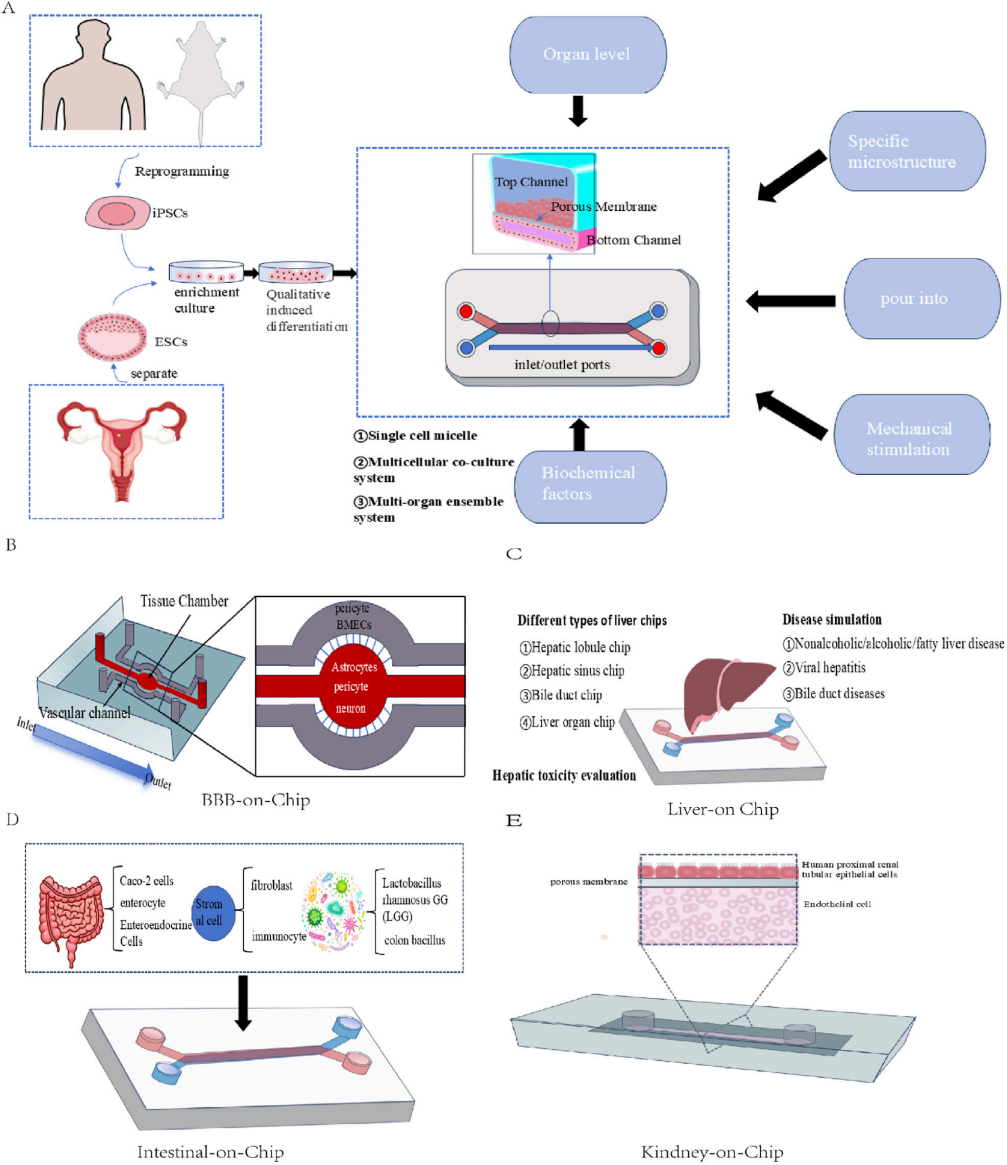
Structural schematic diagram of different types of OoC: **(A)** Basic structural schematic diagram of OoC; **(B)** Structural diagram of the BBB-on-a-chip; **(C)** Structural diagram of LoC; **(D)** Structural diagram of IoC; **(E)** Structural diagram of KoC.

Microfluidics is a science and technology characterized by manipulation of fluids in micron-scale space. It can highly or almost completely integrate many basic operation units in the fields of chemistry and biology, such as sample preparation, reactions, separation, detection, cell culture, sorting and lysis, onto a microchip with an area of only a few square centimetres or even smaller. OoC is an advanced bioengineering platform that integrates microfluidic technology and cell biology (Figure 7A). In living cells, the structure and function of human organs are reconstructed through precise microengineering devices. This innovative model can accurately control fluid flow, simulate the interaction between tissue interfaces and integrate mechanical signals, thus creating a dynamic and multidimensional bionic microenvironment. Compared with traditional static cell culture, OoC more accurately simulates the physiological and mechanical characteristics of the human body and provides a powerful and highly simulated research tool for drug research and development, disease modelling and individualized medical care.

#### 8.1.1 Intestine-on-chip (IOC)

IOC can highly reproduce the physiological and pathological state of the intestine by simulating the dynamic fluid flow, mechanical peristalsis and tissue‒tissue interface of the intestine (Figure 7D). Compared with the traditional Caco-2 cell model, the IOC is more accurate in predicting the permeability of the drug intestinal barrier, and the data are closer to the reality of the human body. In addition, it supports the coculture of intestinal cells and microbiota and studies the interaction between the host and microbiota. For example, Shah et al. (2016) designed a “human‒microorganism interaction system” on the basis of a microfluidic chip. In addition, the IOC constructed from patient cells provides an accurate platform for drug screening and toxicity testing for personalized medicine. Kim et al. (2016) introduced pathogenic *Escherichia coli* and human mononuclear macrophages into IOCs to construct a corresponding pathological model of enteritis. Shin and Kim (2018) introduced dextran sodium sulfate (DSS) into the intestinal cavity and established an injury and recovery model of DSS enteritis *in vitro* for the first time.

#### 8.1.2 BBB-on-chip

The BBB-on-Chip (Figure 7B) usually uses human brain microvascular endothelial cells (hCMECs), astrocytes and neurons. These cells are tightly connected in the chip, simulating the structure of the blood‒brain barrier (Alves et al., 2023). The main function of this kind of chip model is to evaluate the penetration ability of the BBB (size, fat solubility, and efflux transporter activity). Park T. E. et al. (2019) constructed a BBB-on-Chip model by combining hCMECs derived from induced pluripotent stem cells with primary cultured human astrocytes and pericytes. In this model, endothelial cells highly express tight junction proteins and functional efflux pumps, and the selective transcytosis of peptides and antibodies can be observed. Microfluidic chip technology can also monitor the interaction between cells, molecules and nanoparticles in real time and accurately predict the distribution of drugs in cells, blood vessels and surrounding tissues. In addition, this technology highlights its unique advantages when studying the ability of drugs to cross the BBB in the disease state. Shi et al. (2023) innovatively constructed a glioma microfluidic chip model to explore the BBB penetration and efficacy of antiglioma TCM components in the human body and glioma microenvironment. The model combines microfluidic technology to coculture human brain microvascular endothelial cells, astrocytes, pericytes and U251 glioma cells, which highly simulates human BBB function and the glioma microenvironment. HPLC and UV evaluation revealed that TCM components such as flavonoids and resveratrol could cross the BBB and kill U251 cells.

#### 8.1.3 Liver-on-chip (LoC)

LOC (Figure 7C) technology can endow the liver model with more functions, and different LOCs can be constructed as needed (Liu et al., 2024). Liu Y et al. (2022) developed a three-vessel chip based on double-layer microspheres. The chip successfully reproduced the structure of the hepatic lobule *in vitro* and formed a vascularized liver microstructure by constructing a culture zone of hepatic lobule cells and encapsulating hematopoietic stem cells with double-layer microspheres. This design not only simulates the concentration gradient microenvironment *in vivo* but also predicts hepatotoxicity, drug metabolism and tumor invasion mechanisms *in vitro*. With the continuous progress of technology, LoC has become an important means to simulate and create complex three-dimensional TME structures. Özkan et al. (2023) created a three-dimensional vascularized hepatocellular carcinoma chip composed of various cell types, which can simulate liver hardness under normal or cirrhosis conditions and provides a powerful tool for studying the tumor microenvironment and how drug delivery methods can adjust the chemotherapy resistance of hepatocellular carcinoma. The three-dimensional tumor ball invasion detection liver chip constructed by Lee et al. (2021) focuses on monitoring the fusion and invasion potential of HepG2 cells and other types of cells, which provides a new perspective for revealing the tumor invasion mechanism.

#### 8.1.4 Kidney-on-chip (KoC)

KoC (Figure 7E) simulates the functions of glomeruli and renal tubules through microfluidic technology and uses human primary renal cells or induced pluripotent stem cells (iPSCs) to construct physiologically related models (Sateesh et al., 2022). KoC can not only simulate acute kidney injury (AKI), chronic kidney disease (CKD) and end-stage renal disease (ESRD) (Weber et al., 2018; Wilmer et al., 2016) but also provide a platform for evaluating the excretion efficiency and nephrotoxicity of drugs in complex pathological environments and screening potential therapeutic drugs. KoC can simulate various units that make up the kidney. For example, a glomerular chip is constructed from podocytes derived from iPSCs, which can highly simulate the filtration function of the glomerulus (Musah et al., 2017; Musah et al., 2018; Petrosyan et al., 2019). Renal tubular chips focus on the study of drug transport mechanisms and nephrotoxicity (Jang et al., 2013; Sciancalepore et al., 2014; Jansen et al., 2016). In addition, the emergence of multiorgan chip technology has enabled kidney chips to be combined with other organ chips (such as LoCs) (Sateesh et al., 2022).

#### 8.1.5 Application of Organ-on-chip in toxicity prediction

OoCs can be divided into single-organ chips and multiorgan chips. At present, researchers have successfully constructed single-organ chips and multiple-organ chips for the lung (the first one, in 2010), liver, kidney, intestine, heart, brain, blood, bone, skin, nerve, islet and tumor (Ma et al., 2021). OoC has good bionic performance *in vitro* and requires fewer samples and a short detection time. It is convenient, sensitive and rapid, especially in the toxicological evaluation of drugs in the liver, kidney and heart (ZHang et al., 2023). OoC can provide more accurate drug toxicity assessment, especially in the study of drug-induced liver injury and nephrotoxicity. For example, LoC, developed by Jang et al. (2019), can detect various phenotypes of hepatotoxicity, such as steatosis, cholestasis and fibrosis, and evaluate the toxic effects of drugs. In the study conducted by Ewart et al. (2021), 870 LOCs were used to detect 27 drugs with known hepatotoxicity and nonhepatotoxicity. The results show that the liver chip has 80% sensitivity and 100% specificity, and the sensitivity can be improved to 87% by combination with the corrected protein, which indicates that LoC has significant advantages in identifying hepatotoxic drugs. To evaluate nephrotoxicity, Jang et al. (2013) cultured primary renal epithelial cells on a chip for drug transport and nephrotoxicity evaluation. Compared with conventional culture conditions, the toxicity test results of the chip are closer to the *in vivo* situation, and it has potential application in preclinical safety evaluation research.

### 8.2 The application of multi-organ on chip (multi-OoC)

Multi-OoC is a platform that integrates multiple organ models on one chip through microfluidic technology, which can simulate the interaction and physiological functions of multiple organs in the human body. Novak et al. (2020) built an automatic instrument named the “interrogator”, which contains ten kinds of OoCs. The vitality and organ-specific functions of eight different vascularized and dual-channel organ chips (including intestine, liver, kidney, heart, lung, skin, blood‒brain barrier and brain) were successfully maintained for 3 weeks through the Interrogator platform, and the *in vitro* simulation of complex human body systems was realized. On the basis of the technical advantages of this kind of multi-OoC platform, Herland et al. (2020) further carried out drug metabolism research. The metabolic processes of oral nicotine (using intestinal, liver and kidney chips) and intravenous cisplatin (using coupled bone marrow, LoC and koC) were studied via serial chip technology. The results showed that the multi-OoC model showed good predictive ability in the *in vivo* correlation (IVIVT) study of nicotine and cisplatin. In terms of organ PK parameters, the model was the most accurate in predicting liver metabolism; the deviation between the intrahepatic clearance rate of nicotine (CLint = 10.0 mL/min/kg) and the clinical value (12.5 mL/min/kg) was 20%, and the deviation between the CLint prediction of cisplatin in the liver (0.20 mL/min/kg) and the clinical value (0.25 mL/min/kg) was 20%. However, intestinal absorption (nicotine permeability deviation of 21%–98%) and renal excretion (nicotine renal clearance deviation of 75%–98%, cisplatin renal clearance deviation of 99.4%) are still bottlenecks, mainly due to the inability of the IoC to simulate mucus layer dynamics and the lack of active secretion structure of KoC. The prediction of total body exposure was outstanding: the predicted AUC values (48.27 μM h and 252.6 μM h) of cisplatin infusion for 1 h and 3 h were not significantly different from the clinical data (41.94 ± 12.41 μM h and 253.33 ± 100.32 μM h) (p > 0.05), and Bland‒Altman analysis revealed the Cmax. Lin’s Concordance Coefficient and Pearson coefficient show good to very good correlation. Pharmacodynamics (PD) verification further strengthened the model value: after 24 h of cisplatin exposure, the total number of cells in the bone-on-chip model decreased by 78% (p = 0.001), and the number of neutrophils and erythrocytes decreased by 67% (p < 0.04) and 72% (p < 0.002), respectively, which accurately simulated the clinical bone marrow suppression toxicity. Albumin and CYP3A4 in LoC did not change (consistent with human hepatotoxicity loss), but KoC reproduced the early signs of renal injury, with OCT2 upregulation and Pgp downregulation. This organ-specific toxicity response model was closer to that of the human body than was the rat model (long-term experiments in rats revealed the opposite trend). In summary, the model overcomes the limitation of species differences, and the overall prediction data are stronger than those of rodents. To establish clinical reliability in the prediction of core liver metabolism, systemic exposure and organ toxicity (deviation of key parameters ≤20%). These results show that multi-OoC systems have high potential for clinical use.

### 8.3 Application status of OoC technology in ADMET research of TCM

OoC has shown remarkable advantages in the study of ADMET (absorption, distribution, metabolism, excretion and toxicity), especially in simulating complex physiological environments and multiorgan synergistic effects. By constructing OoC and multi-OoC models, researchers can accurately analyse the mechanism of action of traditional Chinese medicine components. For example, Xu et al. (2024) revealed that aconitine caused cardiotoxicity by destroying the structure of the myocardial cell membrane and interfering with the cyclic metabolism of tricarboxylic acid, and Li et al. (2023) demonstrated that ginsenoside Rg3 was metabolized into a more active metabolite in the liver chip, which has greater antitumour potential. The core advantage of multi-OoC lies in its integrity, such as the ability of the intestine‒liver‒kidney combined model to track the absorption, metabolism and excretion path of ginsenoside CK dynamically and to verify that the biological parameters of traditional Chinese medicine components are closer to the *in vivo* data, which overcomes the static limitations of traditional single-cell or animal models (Liu et al., 2020). In addition, the 3D liver microsphere model can maintain the functional expression of hepatocytes through long-term culture and successfully warn of the potential hepatotoxicity of Xiangdan injection (Zhu et al., 2021), highlighting its value in chronic safety assessment. Supplementary Table S5 describes these examples in detail. Chang et al. (2017) studied the biotransformation process and transport mechanism of aristolochic acid I in an organ chip by establishing a model of the KoC–LoC connection and reported that the specific metabolism of aristolochic acid I in human hepatocytes significantly increased its cytotoxicity to human renal proximal tubular epithelial cells. The molecular mechanism of the hepatorenal synergistic toxicity of aristolochic acid I was revealed.

## 9 Conclusion and prospects

*In vitro* models, through controllability, efficiency, and in-depth mechanism analysis, provide an irreplaceable technical platform for the ADMET research of TCM. Its advantage lies not only in overcoming the complexity of the multicomponent system of TCM but also in promoting the transformation of research methods from “overall fuzziness” to “accurate analysis”. However, in the process of analysing and sorting the relevant literature, in the study of ADMET, most studies use more traditional *in vitro* models and lack innovations in models. Moreover, these studies focused mostly on individual biological processes and lacked an examination of the overall mechanism of action of TCM in the body, making it difficult to provide accurate data after the interaction between organs.

This research method has obvious limitations, and comprehensively analysing the metabolic and toxic characteristics of complex systems of TCM is difficult. For example, although monolayer cell culture simplifies the research object, it is difficult to simulate the real physiological environment because of its oversimplification, especially in simulating the interaction between TCM components, the synergistic effect of multiple organs and the influence of microbial communities. The traditional Caco-2 monolayer cell model has difficulty reflecting the influence of intestinal microorganisms on TCM absorption, and a single hepatocyte culture system cannot simulate the complex structure of the liver. In addition, a single model under static culture conditions has difficulty capturing the dynamic process of TCM metabolism, such as the intestinal‒hepatic circulation and secondary transformation of metabolites. With respect to toxicity assessment, a single-cell model often overestimates or underestimates toxicity, and accurately predicting *in vivo* reactions is difficult. These limitations lead to a large gap between the research results and clinical manifestations, which affects the accuracy and predictive ability of ADMET research.

**FIGURE 8:**
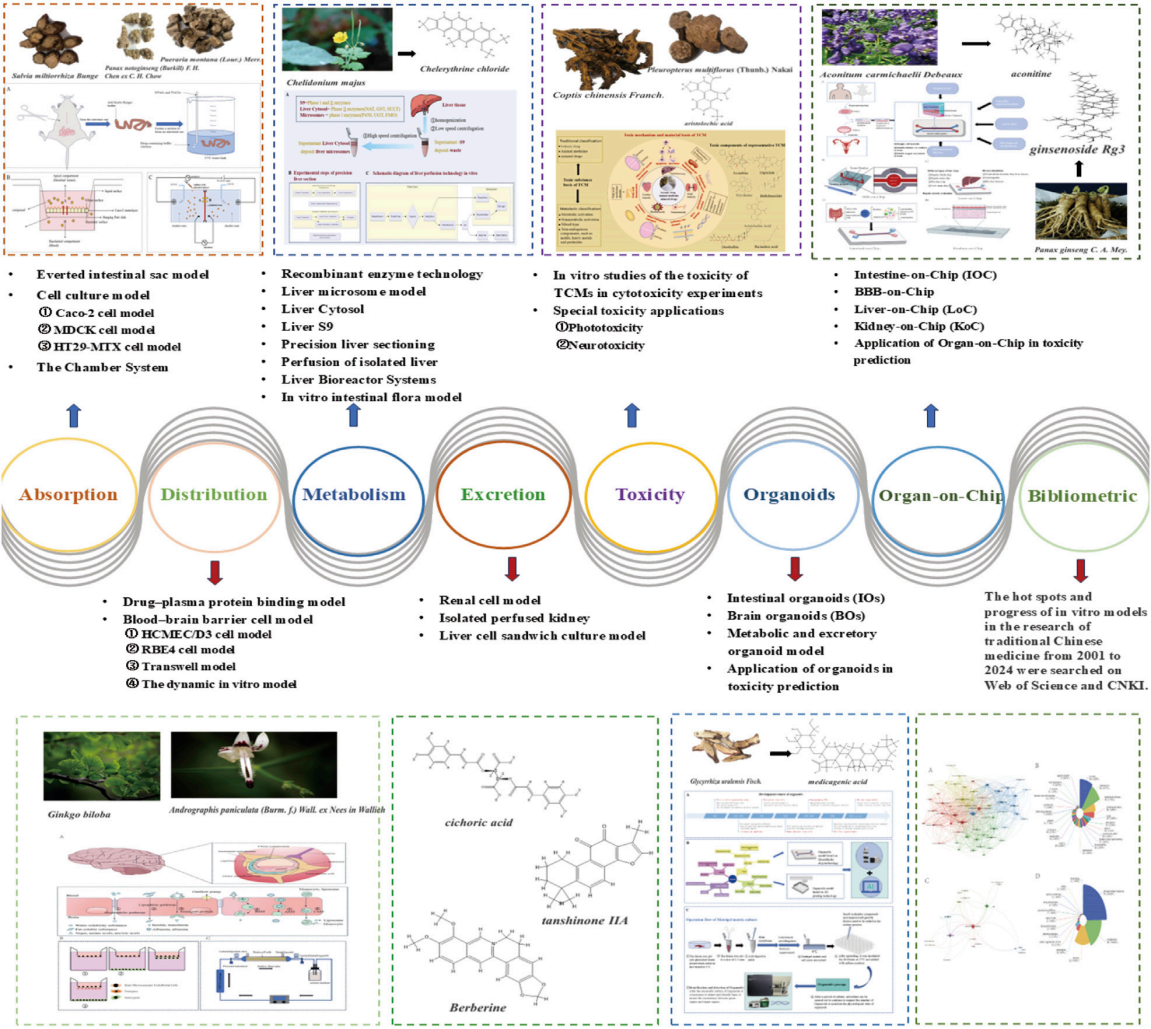
*In vitro* technology and ADMET research in traditional Chinese medicine.

Traditional models and single-model analyses have gradually failed to meet the requirements of modern ADMET research, and the application of *in vitro* models in traditional Chinese medicine (ADMET) needs to overcome the analytical bottleneck of traditional research methods for complex Chinese medicine systems. Through the integration of organoids, OoC, gene editing and multiomics technology, the *in vitro* model has the advantages of high controllability, Qualcomm screening and accurate mechanism analysis. In addition, the combined application of multiple models has also become a trend in drug research. By integrating different levels of *in vitro* models (such as coculture cell models, organoids, and OoC), these technologies can help the complex physiological process of traditional Chinese medicine in the human body. This method is not limited to simulating the biotransformation of drugs in an organ and enables the dynamic and real-time comprehensive monitoring of drugs. The emergence of cutting-edge technology and multimodel joint models provides innovative research tools for intestinal absorption competition, transorgan distribution dynamics, metabolic transformation networks and multichannel excretion interactions of TCMs. This makes the evaluation of TCM no longer specific and provides a more comprehensive and accurate multidimensional analysis. Figure 8 provides a summary and overview of the content of this review.

## Abbreviations

ASCs, Adult stem cells; AP, Apical Compartment; BL, Basolateral Compartment; BBB, Blood‒brain barrier; BMECs, Brain Microvascular Endothelial Cells; BOs, Brain organoids; CD, Circular Dichroism; DDASS, drug dissolution/absorption simulating system; DIV, Dynamic *in vitro*; ESCs, Embryonic stem cells; HPFA, High performance frontal analysis; iPSCs, induced pluripotent stem cells; LOs, liver organoids; IoC, Intestine-on-Chip; KOs, Kidney organoids; LoC, Liver-on-Chip; MDCK, Madin-Darby Canine Kidney; MRP, Multidrug Resistance Protein; NVU, Neurovascular Unit; OoC, Organ-on-Chip; PAMPA, Parallel Artificial Membrane Permeability Assay; P-gp, P-Glycoprotein; PSCs, Pluripotent stem cells; SPR, Surface plasmon resonance; TCM, traditional Chinese medicine; TREE, Trans-Epithelial Electrical Resistance; UGT, Uridine Diphosphate-Glycosyltransferase.

## References

[B1] Adler M. Ramm S. Hafner M. Muhlich J. L. Gottwald E. M. Weber E. (2016). A quantitative approach to screen for nephrotoxic compounds *in vitro* . J. Am. Soc. Nephrol. 27, 1015–1028. 10.1681/ASN.2015010060 26260164 PMC4814182

[B2] Alam M. A. Al-Jenoobi F. I. Al-Mohizea A. M. (2012). Everted gut sac model as a tool in pharmaceutical research: limitations and applications. J. Pharm. Pharmacol. 64, 326–336. 10.1111/j.2042-7158.2011.01391.x 22309264

[B3] Alves A. D. H. Nucci M. P. Ennes do Valle N. M. Missina J. M. Mamani J. B. Rego G. N. A. (2023). Current overview of induced pluripotent stem cell-based blood-brain barrier-on-a-chip. World J. Stem Cells 15, 632–653. 10.4252/wjsc.v15.i6.632 37424947 PMC10324508

[B4] Andjelkovic A. V. Stamatovic S. M. Phillips C. M. Martinez-Revollar G. Keep R. F. (2020).'Modeling blood-brain barrier pathology in cerebrovascular disease *in vitro:* current and future paradigms. Fluids Barriers CNS 17, 44. 10.1186/s12987-020-00202-7 32677965 PMC7367394

[B5] Awortwe C. Fasinu P. S. Rosenkranz B. (2014). Application of Caco-2 cell line in herb-drug interaction studies: current approaches and challenges. J. Pharm. Pharm. Sci. 17, 1–19. 10.18433/j30k63 24735758 PMC4355448

[B6] Bale S. S. Moore L. Yarmush M. Jindal R. (2016). Emerging *in vitro* liver technologies for drug metabolism and inter-organ interactions. Tissue Eng. Part B Rev. 22, 383–394. 10.1089/ten.TEB.2016.0031 27049038 PMC5065030

[B7] Bao H. Hou J. Hu H. Li Y. Zheng L. Huang Y. (2019). Determination of plasma protein binding rates of nine compounds of Inula cappa extraction based on method of equilibrium dialysis. China J. Chin. Mater Med. 44, 1475–1484. 10.19540/j.cnki.cjcmm.20190116.007 31090307

[B8] Bao Y. Shen F. Li Y. Chen D. Lu H. (2020). Toxicity and mechanism of polygoni multiflori radix alcohol extract on L02 cells. Chin. J. Exp. Tradit. Med. Formulae 26, 23–28. 10.13422/j.cnki.syfjx.20201022

[B9] Barthe L. Woodley J. F. Kenworthy S. Houin G. (1998). An improved everted gut sac as a simple and accurate technique to measure paracellular transport across the small intestine. Eur. J. Drug Metab. Pharmacokinet. 23, 313–323. 10.1007/BF03189357 9725499

[B10] Behrens I. Stenberg P. Artursson P. Kissel T. (2001). Transport of lipophilic drug molecules in a new mucus-secreting cell culture model based on HT29-MTX cells. Pharm. Res. 18, 1138–1145. 10.1023/a:1010974909998 11587485

[B11] Bell C. C. Hendriks D. F. Moro S. M. Ellis E. Walsh J. Renblom A. (2016). Characterization of primary human hepatocyte spheroids as a model system for drug-induced liver injury, liver function and disease. Sci. Rep. 6, 25187. 10.1038/srep25187 27143246 PMC4855186

[B12] Birey F. Andersen J. Makinson C. D. Islam S. Wei W. Huber N. (2017). Assembly of functionally integrated human forebrain spheroids. Nature 545, 54–59. 10.1038/nature22330 28445465 PMC5805137

[B13] Braun A. Hämmerle S. Suda K. Rothen-Rutishauser B. Günthert M. Krämer S. D. (2000). Cell cultures as tools in biopharmacy. Eur. J. Pharm. Sci. 11 (Suppl. 2), S51–S60. 10.1016/s0928-0987(00)00164-0 11033427

[B14] Bussolari S. R. Dewey C. F. Jr. Gimbrone M. A. Jr. (1982). Apparatus for subjecting living cells to fluid shear stress. Rev. Sci. Instrum. 53, 1851–1854. 10.1063/1.1136909 7156852

[B15] Cappuccio G. Khalil S. M. Osenberg S. Li F. Maletic-Savatic M. (2023). Mass spectrometry imaging as an emerging tool for studying metabolism in human brain organoids. Front. Mol. Biosci. 10, 1181965. 10.3389/fmolb.2023.1181965 37304070 PMC10251497

[B16] Cecchelli R. Berezowski V. Lundquist S. Culot M. Renftel M. Dehouck M. P. (2007). Modelling of the blood-brain barrier in drug discovery and development. Nat. Rev. Drug Discov. 6, 650–661. 10.1038/nrd2368 17667956

[B17] Chang S. Y. Weber E. J. Sidorenko V. S. Chapron A. Yeung C. K. Gao C. (2017). Human liver-kidney model elucidates the mechanisms of aristolochic acid nephrotoxicity. JCI Insight 2, e95978. 10.1172/jci.insight.95978 29202460 PMC5752374

[B18] Chen G. Bei B. Feng Y. Li X. Jiang Z. Si J. Y. (2019). Glycyrrhetinic acid maintains intestinal homeostasis *via* HuR. Front. Pharmacol. 10, 535. 10.3389/fphar.2019.00535 31156441 PMC6531911

[B19] Chen H. Wang X. Pan G. Li T. (2017a). Detection of the human plasma protein binding rate of periplocin, periplocymarin and periplogenin. J. Liaoning Univ. Tradit. Chin. Med. 19, 32–35. 10.13194/j.issn.1673-842x.2017.07.007

[B20] Chen H. Xie X. Peng C. (2017b). *In vitro* cytotoxicity evaluation indicator and test methods applied in toxicology of traditional Chinese medicine. Chin. J. Exp. Traditional Med. Formulae 23, 202–210. 10.13422/j.cnki.syfjx.2017220202

[B21] Chen W. Y. Evangelista E. A. Yang J. Kelly E. J. Yeung C. K. (2021). Kidney organoid and microphysiological kidney chip models to accelerate drug development and reduce animal testing. Front. Pharmacol. 12, 695920. 10.3389/fphar.2021.695920 34381363 PMC8350564

[B22] Chen X. (2011). Experimental study on metabolism and bio-transformation of ginsenoside Rg1by intestinal enzyme and microflora in rats. Chin. J. Exp. Tradit. Med. Form. 17, 210–212. 10.13422/j.cnki.syfjx.2011.11.063

[B23] Chen X. Lu N. Huang S. Zhang Y. Liu Z. Wang X. (2023). Assessment of doxorubicin toxicity using human cardiac organoids: a novel model for evaluating drug cardiotoxicity. Chem. Biol. Interact. 386, 110777. 10.1016/j.cbi.2023.110777 37879593

[B24] Chen X. M. Elisia I. Kitts D. D. (2010). Defining conditions for the co-culture of Caco-2 and HT29-MTX cells using Taguchi design. J. Pharmacol. Toxicol. Methods 61, 334–342. 10.1016/j.vascn.2010.02.004 20159047

[B25] Chi H. Li Z. Wang Y. Xu F. He Z. Yang X. (2021). Progress in researches on *in vitro* alternative model for nephrotoxicityevaluation. Chin. J. Public Health. 37, 1035–1040. 10.11847/zgggws1126789

[B26] Crespo M. Vilar E. Tsai S. Y. Chang K. Amin S. Srinivasan T. (2018). Corrigendum: colonic organoids derived from human induced pluripotent stem cells for modeling colorectal cancer and drug testing. Nat. Med. 24, 526. 10.1038/nm0418-526a 29634683

[B27] Cucullo L. Couraud P. O. Weksler B. Romero I. A. Hossain M. Rapp E. (2008). Immortalized human brain endothelial cells and flow-based vascular modeling: a marriage of convenience for rational neurovascular studies. J. Cereb. Blood Flow. Metab. 28, 312–328. 10.1038/sj.jcbfm.9600525 17609686

[B28] Dolmetsch R. Geschwind D. H. (2011). The human brain in a dish: the promise of iPSC-derived neurons. Cell 145, 831–834. 10.1016/j.cell.2011.05.034 21663789 PMC3691069

[B29] Du H. Xue Z. Xia Z. He S. Yang J. Zhu Y. (2022). Construction of 3D blood-brain barrier organoid oxygen-glucose deprivation model and exploration of the protective effect of guanxinning injection. Acta Pharm. Sin. 57, 3086–3094. 10.16438/j.0513-4870.2021-1871

[B30] Du Y. Lo E. Ali S. Khademhosseini A. (2008). Directed assembly of cell-laden microgels for fabrication of 3D tissue constructs. Proc. Natl. Acad. Sci. U. S. A. 105, 9522–9527. 10.1073/pnas.0801866105 18599452 PMC2474514

[B31] Eade K. Giles S. Harkins-Perry S. Friedlander M. (2021). Toxicity screens in human retinal organoids for pharmaceutical discovery. J. Vis. Exp. 10.3791/62269 33749682

[B32] Elbadawy M. Hayashi K. Ayame H. Ishihara Y. Abugomaa A. Shibutani M. (2021). Anti-cancer activity of amorphous curcumin preparation in patient-derived colorectal cancer organoids. Biomed. Pharmacother. 142, 112043. 10.1016/j.biopha.2021.112043 34411919

[B33] Elferink M. G. Olinga P. Draaisma A. L. Merema M. T. Bauerschmidt S. Polman J. (2008). Microarray analysis in rat liver slices correctly predicts *in vivo* hepatotoxicity. Toxicol. Appl. Pharmacol. 229, 300–309. 10.1016/j.taap.2008.01.037 18346771

[B34] Ewart L. Apostolou A. Briggs S. A. Carman C. V. Chaff J. T. Heng A. R. (2021). Qualifying a human liver-chip for predictive toxicology: performance assessment and economic implications. bioRxiv. 10.1101/2021.12.14.472674 PMC9727064 36473994

[B35] Fan X. Chai L. Zhang H. Wang Y. Zhang B. Gao X. (2015). Borneol depresses P-Glycoprotein function by a NF-κB signaling mediated mechanism in a blood brain barrier *in vitro* model. Int. J. Mol. Sci. 16, 27576–27588. 10.3390/ijms161126051 26593909 PMC4661909

[B36] Fang L. Tao W. Cheng H. Lii D. Wang Y. Xu A. (2023). Research progress on mechanism of uplink of traditional Chinese medicine and its brain targeting preparations. Chin. J. Herb. Med. 54, 3312–3321. 10.7501/j.issn.0253-2670.2023.10.028

[B37] Farzaneh Z. Abbasalizadeh S. Asghari-Vostikolaee M. H. Alikhani M. Cabral J. M. S. Baharvand H. (2020). Dissolved oxygen concentration regulates human hepatic organoid formation from pluripotent stem cells in a fully controlled bioreactor. Biotechnol. Bioeng. 117, 3739–3756. 10.1002/bit.27521 32725885

[B38] Fogh J. Wright W. C. Loveless J. D. (1977). Absence of HeLa cell contamination in 169 cell lines derived from human tumors. J. Natl. Cancer Inst. 58, 209–214. 10.1093/jnci/58.2.209 833871

[B39] Friedberg T. Pritchard M. P. Bandera M. Hanlon S. P. Yao D. McLaughlin L. A. (1999). Merits and limitations of recombinant models for the study of human P450-mediated drug metabolism and toxicity: an intralaboratory comparison. Drug Metab. Rev. 31, 523–544. 10.1081/dmr-100101934 10335451

[B40] Fu S. Rowe A. Ramzan I. (2012). Kavalactone metabolism in the isolated perfused rat liver. Phytother. Res. 26, 1813–1816. 10.1002/ptr.4656 22407838

[B41] Garreta E. Kamm R. D. Chuva de Sousa Lopes S. M. Lancaster M. A. Weiss R. Trepat X. (2021). Rethinking organoid technology through bioengineering. Nat. Mater 20, 145–155. 10.1038/s41563-020-00804-4 33199860

[B42] Georgiev T. Iliev R. Mihailova S. Hadzhibozheva P. Ilieva G. Kamburova M. A. (2011). The isolated perfused kidney models - certain aspects.

[B43] Gevaert E. Dollé L. Billiet T. Dubruel P. van Grunsven L. van Apeldoorn A. (2014). High throughput micro-well generation of hepatocyte micro-aggregates for tissue engineering. PLoS One 9, e105171. 10.1371/journal.pone.0105171 25133500 PMC4136852

[B44] Gibson G. R. Cummings J. H. Macfarlane G. T. (1988). Use of a three-stage continuous culture system to study the effect of mucin on dissimilatory sulfate reduction and methanogenesis by mixed populations of human gut bacteria. Appl. Environ. Microbiol. 54, 2750–2755. 10.1128/aem.54.11.2750-2755.1988 3214155 PMC204367

[B45] Gijbels E. Vanhaecke T. Vinken M. (2019). Establishment of sandwich cultures of primary human hepatocytes. Methods Mol. Biol. 1981, 325–333. 10.1007/978-1-4939-9420-5_21 31016664

[B46] Gradolatto A. Canivenc-Lavier M. C. Basly J. P. Siess M. H. Teyssier C. (2004). Metabolism of apigenin by rat liver phase I and phase ii enzymes and by isolated perfused rat liver. Drug Metab. Dispos. 32, 58–65. 10.1124/dmd.32.1.58 14709621

[B47] Gu S. Fan Y. Wang L. Feng G. Liu S. Sheng Y. (2015). Analysis on release characteristics of sustained release traditional Chinese medicine preparation of Leigongteng bilayer tablets based on drug simulating system. Chin. J. Exp. Tradit. Med. Formulae 21, 1–6. 10.13422/j.cnki.syfjx.2015150001

[B48] Gu S. Wu G. Lu D. Wang Y. Tang L. Zhang W. (2023). Human kidney organoids model of esculentoside A nephrotoxicity to investigate the role of epithelial-mesenchymal transition *via* STING signaling. Toxicol. Lett. 373, 172–183. 10.1016/j.toxlet.2022.11.019 36460195

[B49] Hartung T. Bremer S. Casati S. Coecke S. Corvi R. Fortaner S. (2003). ECVAM's response to the changing political environment for alternatives: consequences of the european union chemicals and cosmetics policies. Altern. Lab. Anim. 31, 473–481. 10.1177/026119290303100504 15598174

[B50] He S. Lin M. Jiang Y. Wang S. Shi J. (2018). Intestinal absorption model of component of Chinese medicinal:its research progress and reflection. Acta Chin. Med. Pharm. 46, 121–124. 10.19664/j.cnki.1002-2392.180100

[B51] He W. Zhao Y. Liu X. Sun Q. Zhang Z. Wang S. (2022). Effect of curcumin on the activity of cytochrome P450 2C8 in rat and human liver microsomes. Chin. J. Biochem. Pharm. 38, 683–687. 10.13699/j.cnki.1001-6821.2022.07.016

[B52] Herland A. Maoz B. M. Das D. Somayaji M. R. Prantil-Baun R. Novak R. (2020). Quantitative prediction of human pharmacokinetic responses to drugs *via* fluidically coupled vascularized organ chips. Nat. Biomed. Eng. 4, 421–436. 10.1038/s41551-019-0498-9 31988459 PMC8011576

[B53] Hilgendorf C. Spahn-Langguth H. Regårdh C. G. Lipka E. Amidon G. L. Langguth P. (2000). 'Caco-2 *versus* Caco-2/HT29-MTX co-cultured cell lines: permeabilities *via* diffusion, inside- and outside-directed carrier-mediated transport. J. Pharm. Sci. 89, 63–75. 10.1002/(SICI)1520-6017(200001)89:1<63::AID-JPS7>3.0.CO;2-6 10664539

[B54] Hong Y. Chan N. Begum A. N. (2019). Deriving neural cells from pluripotent stem cells for nanotoxicity testing. Methods Mol. Biol. 1894, 57–72. 10.1007/978-1-4939-8916-4_4 30547455

[B55] Huang J. Zhang J. Bai J. Xu W. Wu D. Qiu X. (2016). LC-MS/MS determination and interaction of the main components from the traditional Chinese drug pair Danshen-Sanqi based on rat intestinal absorption. Biomed. Chromatogr. 30, 1928–1934. 10.1002/bmc.3768 27228090

[B56] Irvine J. D. Takahashi L. Lockhart K. Cheong J. Tolan J. W. Selick H. E. (1999). 'MDCK (Madin-Darby canine kidney) cells: a tool for membrane permeability screening. J. Pharm. Sci. 88, 28–33. 10.1021/js9803205 9874698

[B57] Israel M. A. Yuan S. H. Bardy C. Reyna S. M. Mu Y. Herrera C. (2012). Probing sporadic and familial Alzheimer's disease using induced pluripotent stem cells. Nature 482, 216–220. 10.1038/nature10821 22278060 PMC3338985

[B58] Jang K. J. Mehr A. P. Hamilton G. A. McPartlin L. A. Chung S. Suh K. Y. (2013). Human kidney proximal tubule-on-a-chip for drug transport and nephrotoxicity assessment. Integr. Biol. (Camb) 5, 1119–1129. 10.1039/c3ib40049b 23644926

[B59] Jang K. J. Otieno M. A. Ronxhi J. Lim H. K. Ewart L. Kodella K. R. (2019). Reproducing human and cross-species drug toxicities using a Liver-chip. Sci. Transl. Med. 11, eaax5516. 10.1126/scitranslmed.aax5516 31694927

[B60] Jansen J. Fedecostante M. Wilmer M. J. Peters J. G. Kreuser U. M. van den Broek P. H. (2016). Bioengineered kidney tubules efficiently excrete uremic toxins. Sci. Rep. 6, 26715. 10.1038/srep26715 27242131 PMC4886219

[B61] Jenkinson S. E. Chung G. W. van Loon E. Bakar N. S. Dalzell A. M. Brown C. D. (2012). The limitations of renal epithelial cell line HK-2 as a model of drug transporter expression and function in the proximal tubule. Pflugers Arch. 464, 601–611. 10.1007/s00424-012-1163-2 23014881

[B62] Kaisar M. A. Sajja R. K. Prasad S. Abhyankar V. V. Liles T. Cucullo L. (2017). New experimental models of the blood-brain barrier for CNS drug discovery. Expert Opin. Drug Discov. 12, 89–103. 10.1080/17460441.2017.1253676 27782770 PMC5521006

[B63] Khalil S. M. Cappuccio G. Li F. Maletic-Savatic M. (2024). Molecular imaging of human brain organoids using mass spectrometry. J. Vis. Exp. 10.3791/66997 39400180

[B64] Khattak S. U. Ahmad I. Usmanghani K. Qazi M. S. (2012). *In vitro* evaluation of betamethasone esters for phototoxic potential. Drug Chem. Toxicol. 35, 43–47. 10.3109/01480545.2011.588441 21834694

[B65] Kim D. Garrett S. H. Sens M. A. Somji S. Sens D. A. (2002). Metallothionein isoform 3 and proximal tubule vectorial active transport. Kidney Int. 61, 464–472. 10.1046/j.1523-1755.2002.00153.x 11849386

[B66] Kim H. J. Li H. Collins J. J. Ingber D. E. (2016). Contributions of microbiome and mechanical deformation to intestinal bacterial overgrowth and inflammation in a human gut-on-a-chip. Proc. Natl. Acad. Sci. U. S. A. 113, E7–E15. 10.1073/pnas.1522193112 26668389 PMC4711860

[B67] Kim Y. S. Kim J. J. Cho K. H. Jung W. S. Moon S. K. Park E. K. (2008). Biotransformation of ginsenoside Rb1, crocin, amygdalin, geniposide, puerarin, ginsenoside Re, hesperidin, poncirin, glycyrrhizin, and baicalin by human fecal microflora and its relation to cytotoxicity against tumor cells. J. Microbiol. Biotechnol. 18, 1109–1114. 18600055

[B68] Knobeloch D. Ehnert S. Schyschka L. Büchler P. Schoenberg M. Kleeff J. (2012). Human hepatocytes: isolation, culture, and quality procedures. Methods Mol. Biol. 806, 99–120. 10.1007/978-1-61779-367-7_8 22057448

[B69] Lahiani A. Brand-Yavin A. Yavin E. Lazarovici P. (2018). Neuroprotective effects of bioactive compounds and MAPK pathway modulation in “Ischemia”-Stressed PC12 pheochromocytoma cells. Brain Sci. 8, 32. 10.3390/brainsci8020032 29419806 PMC5836051

[B70] Lake B. G. Price R. J. (2013). Evaluation of the metabolism and hepatotoxicity of xenobiotics utilizing precision-cut slices. Xenobiotica 43, 41–53. 10.3109/00498254.2012.734643 23131042

[B71] Lancaster M. A. Renner M. Martin C. A. Wenzel D. Bicknell L. S. Hurles M. E. (2013). Cerebral organoids model human brain development and microcephaly. Nature 501, 373–379. 10.1038/nature12517 23995685 PMC3817409

[B72] Lechner C. Mönning U. Reichel A. Fricker G. (2021). Potential and limits of kidney cells for evaluation of renal excretion. Pharm. (Basel) 14, 908. 10.3390/ph14090908 PMC8464824 34577608

[B73] Lee J. Ung A. Kim H. Lee K. Cho H. J. Bandaru P. (2021). Engineering liver microtissues to study the fusion of HepG2 with mesenchymal stem cells and invasive potential of fused cells. Biofabrication 14, 014104. 10.1088/1758-5090/ac36de 34740205

[B74] Lee Y. S. Yi J. S. Lim H. R. Kim T. S. Ahn I. Y. Ko K. (2017). Phototoxicity evaluation of pharmaceutical substances with a reactive oxygen species assay using ultraviolet A. Toxicol. Res. 33, 43–48. 10.5487/TR.2017.33.1.043 28133512 PMC5266373

[B75] Lesuffleur T. Barbat A. Luccioni C. Beaumatin J. Clair M. Kornowski A. (1991). Dihydrofolate reductase gene amplification-associated shift of differentiation in methotrexate-adapted HT-29 cells. J. Cell Biol. 115, 1409–1418. 10.1083/jcb.115.5.1409 1955481 PMC2289245

[B76] Li B. Wang J. Cai T. Guo J. Teng F. Zhu Y. (2019). Effect of tongfengning serum containing on expression of urate transporter in HK-2 induced by uric acid. Chin. J. Exp. Tradit. Med. Formulae 7. 10.13422/j.cnki.syfjx.20192139

[B77] Li C. Lin Q. Zhuang X. Xie J. Li Y. (2010). *'In vitro* O-demethylation of rotundine by recombinant human CYP isoenzymes. Acta Pharm. Sin. 45, 307–313. 10.16438/j.0513-4870.2010.03.022 21351505

[B78] Li D. Han Y. Yu T. Meng X. Yu Q. Wang J. (2011). Chinese journal of clinical pharmacology and therapeutics. Chin. J. Clin. Pharmacol. Ther. 16, 688–694.

[B79] Li J. Xun L. Li H. Zhao Q. (2015). Evaluation and screening for biological activity of components in Chinese materia medica. Chin. J. Herb. Med. 46, 588–594. 10.7501/j.issn.0253-2670.2015.04.025

[B80] Li X. An R. Liang K. Wang X. You L. (2018). Phototoxicity of traditional chinese medicine (TCM). Toxicol. Res. (Camb) 7, 1012–1019. 10.1039/c8tx00141c 30542599 PMC6240804

[B81] Li X. Zheng M. Xu B. Li D. Shen Y. Nie Y. (2021). Generation of offspring-producing 3D ovarian organoids derived from female germline stem cells and their application in toxicological detection. Biomaterials 279, 121213. 10.1016/j.biomaterials.2021.121213 34715637

[B82] Li Y. Sun X. Liu H. Huang L. Meng G. Ding Y. (2019). Development of human *in vitro* brain-blood barrier model from induced pluripotent stem cell-derived endothelial cells to predict the *in vivo* permeability of drugs. Neurosci. Bull. 35, 996–1010. 10.1007/s12264-019-00384-7 31079318 PMC6864025

[B83] Li Z. Li J. Sun M. Men L. Wang E. Zhao Y. (2023). Analysis of metabolites and metabolism-mediated biological activity assessment of ginsenosides on microfluidic co-culture system. Front. Pharmacol. 14, 1046722. 10.3389/fphar.2023.1046722 36794280 PMC9922736

[B84] Lian W. Yang C. Lin Y. Tan H. Xiao C. Ma Z. (2021). Cytotoxicity of coptisine *via* endoplasmic reticulum stress PERK-ATF4-CHOP pathway and mitochondrial damage pathway in L02 cells. Milit. Med. Sci. 45, 834–842. 10.7644/j.issn.1674-9960.2021.11.008

[B85] Liang R. Song X. Ge W. Zhang F. Dai Z. Li N. (2018). Biliary excretion characteristics of berberine, palmatine and jateorhizine in sandwich-cultured rat hepatocytes. Chin. Pharmacol. Bull. 34, 250–256. 10.3969/j.issn.1001-1978.2018.02.020

[B86] Liang W. (2020). Study on the effect and mechanism of bilobalide on modulating blood-brain barrier permeability. Guangdong Pharmaceutical University. Master's thesis.

[B87] Liao Q. Yao Y. Xie Z. Zhang L. Zeng Y. (2011). Absorption mechanism of andrographolide in human Caco-2 cell monolayer model. Chin. Tradit. Herb. Drugs 42, 1363–1366.

[B88] Liao Z. G. Tang T. Guan X. J. Dong W. Zhang J. Zhao G. W. (2016). Improvement of transmembrane transport mechanism study of imperatorin on P-Glycoprotein-Mediated drug transport. Molecules 21, 1606. 10.3390/molecules21121606 27886150 PMC6274566

[B89] Lippmann E. S. Azarin S. M. Palecek S. P. Shusta E. V. (2020). Commentary on human pluripotent stem cell-based blood-brain barrier models. Fluids Barriers CNS 17, 64. 10.1186/s12987-020-00222-3 33076946 PMC7574179

[B90] Liu C. Wang Y. Ma Z. Liang Q. Xiao C. Tan H. (2014). Cytotoxic effect of veratrine hydrochloride on HepG2 cells and its possible mechanism. Chin. J. Pharmacol. Toxicol. 28, 391–397. 10.3867/j.issn.1000-3002.2014.03.014

[B91] Liu D. Jiao S. Wei J. Zhang X. Pei Y. Pei Z. (2020). Investigation of absorption, metabolism and toxicity of ginsenosides compound K based on human organ chips. Int. J. Pharm. 587, 119669. 10.1016/j.ijpharm.2020.119669 32702454

[B92] Liu J. Du Y. Xiao X. Tan D. He Y. Qin L. (2024). Construction of *in vitro* liver-on-a-chip models and application progress. Biomed. Eng. Online 23, 33. 10.1186/s12938-024-01226-y 38491482 PMC10941602

[B93] Liu J. Feng C. Zhang M. Song F. Liu H. (2022). Design and fabrication of a Liver-on-a-chip reconstructing tissue-tissue interfaces. Front. Oncol. 12, 959299. 10.3389/fonc.2022.959299 35992870 PMC9389071

[B94] Liu L. Yang Y. Wang L. Jiang X. (2019). Study of effect of rifampin and tanshinone ⅡA on pravastatin transport *via* BSEP in sandwich-cultured rat hepatocytes. Chin. Pharm. Bull. 35, 978–985.

[B95] Liu L. Zhang J. (2018). Research progress of drug hepatic metabolism models *in vitro* . Chin. J. Vet. Sci. 38, 2015–2019. 10.16303/j.cnki.1005-4545.2018.10.32

[B96] Liu T. Guo C. Zhao X. (2014). *In vitro* cytotoxicity evaluation in toxicology. Chin. Bull. Life Sci. 26, 319–324. 10.13376/j.cbls/2014047

[B97] Liu X. Chen X. Zhu Y. Wang K. Wang Y. (2017). Effect of magnolol on cerebral injury and blood brain barrier dysfunction induced by ischemia-reperfusion *in vivo* and *in vitro* . Metab. Brain Dis. 32, 1109–1118. 10.1007/s11011-017-0004-6 28378105

[B98] Liu X. Liu Y. Cheng M. Xiao H. (2016). Acute nephrotoxicity of aristolochic acid *in vitro:* metabolomics study for intracellular metabolic time-course changes. Biomarkers 21, 233–242. 10.3109/1354750x.2015.1134660 26846302

[B99] Liu Y. Zhang D. Li C. Zhang Z. Zhang A. Dong S. (2022). Investigation of metabolic stability and metabolic enzyme phaenotypes of chebulinic acid in different species of liver microsomes. Drug Eval. Res. 45, 864–870. 10.7501/j.issn.1674-6376.2022.05.007

[B100] Ma C. Peng Y. Li H. Chen W. (2021). Organ-on-a-Chip: a new paradigm for drug development. Trends Pharmacol. Sci. 42, 119–133. 10.1016/j.tips.2020.11.009 33341248 PMC7990030

[B101] Maier-Salamon A. Hagenauer B. Reznicek G. Szekeres T. Thalhammer T. Jäger W. (2008). Metabolism and disposition of resveratrol in the isolated perfused rat liver: role of Mrp2 in the biliary excretion of glucuronides. J. Pharm. Sci. 97, 1615–1628. 10.1002/jps.21057 17724663

[B102] Mao X. Wang Q. (2023). *In vitro* metabolic activation of chelerythrine chloride mediated by P450 enzymes'. J. Shenyang. Pharm. Univ., 1–9.

[B103] Marrero D. Pujol-Vila F. Vera D. Gabriel G. Illa X. Elizalde-Torrent A. (2021). Gut-on-a-chip: mimicking and monitoring the human intestine. Biosens. Bioelectron. 181, 113156. 10.1016/j.bios.2021.113156 33761417

[B104] Messner S. Agarkova I. Moritz W. Kelm J. M. (2013). Multi-cell type human liver microtissues for hepatotoxicity testing. Arch. Toxicol. 87, 209–213. 10.1007/s00204-012-0968-2 23143619 PMC3535351

[B105] Meyer D. van der Kamp J. Asp N. G. Jones J. M. Gertjan S. (2004). The effect of various inulins and *Clostridium difficile* on the metabolic activity and composition of the human colonic microbiota *in vitro* . 237, 253. 10.3920/9789086866625_020

[B106] Middendorp S. Schneeberger K. Wiegerinck C. L. Mokry M. Akkerman R. D. van Wijngaarden S. (2014). Adult stem cells in the small intestine are intrinsically programmed with their location-specific function. Stem Cells 32, 1083–1091. 10.1002/stem.1655 24496776

[B107] Minekus M. (2015). “The TNO gastro-intestinal model (TIM),” in The impact of food bioactives on health: *in vitro* and *ex vivo* models. Editors Verhoeckx K. Cotter P. López-Expósito I. Kleiveland C. Lea T. Mackie A. (Springer). 29787065

[B108] Mizoi K. Okada R. Mashimo A. Masuda N. Itoh M. Ishida S. (2024). Novel screening system for biliary excretion of drugs using human cholangiocyte organoid monolayers with directional drug transport. Biol. Pharm. Bull. 47, 427–433. 10.1248/bpb.b23-00655 38369341

[B109] Morizane R. Lam A. Q. Freedman B. S. Kishi S. Valerius M. T. Bonventre J. V. (2015). Nephron organoids derived from human pluripotent stem cells model kidney development and injury. Nat. Biotechnol. 33, 1193–1200. 10.1038/nbt.3392 26458176 PMC4747858

[B110] Mun S. J. Ryu J. S. Lee M. O. Son Y. S. Oh S. J. Cho H. S. (2019). Generation of expandable human pluripotent stem cell-derived hepatocyte-like liver organoids. J. Hepatol. 71, 970–985. 10.1016/j.jhep.2019.06.030 31299272

[B111] Musah S. Dimitrakakis N. Camacho D. M. Church G. M. Ingber D. E. (2018). Directed differentiation of human induced pluripotent stem cells into mature kidney podocytes and establishment of a glomerulus chip. Nat. Protoc. 13, 1662–1685. 10.1038/s41596-018-0007-8 29995874 PMC6701189

[B112] Musah S. Mammoto A. Ferrante T. C. Jeanty S. S. F. Hirano-Kobayashi M. Mammoto T. (2017). Mature induced-pluripotent-stem-cell-derived human podocytes reconstitute kidney glomerular-capillary-wall function on a chip. Nat. Biomed. Eng. 1, 0069. 10.1038/s41551-017-0069 29038743 PMC5639718

[B113] Mustafa M. G. Khan Md G. M. Nguyen D. Iqbal S. (2018). “Chapter 13 - techniques in biotechnology: essential for industry,” in Omics technologies and bio-engineering. Editors Barh D. Azevedo V. (Academic Press).

[B114] Nakagawa S. Deli M. A. Kawaguchi H. Shimizudani T. Shimono T. Kittel A. (2009). A new blood-brain barrier model using primary rat brain endothelial cells, pericytes and astrocytes. Neurochem. Int. 54, 253–263. 10.1016/j.neuint.2008.12.002 19111869

[B115] Nishihara H. Gastfriend B. D. Soldati S. Perriot S. Mathias A. Sano Y. (2020). Advancing human induced pluripotent stem cell-derived blood-brain barrier models for studying immune cell interactions. Faseb J. 34, 16693–16715. 10.1096/fj.202001507RR 33124083 PMC7686106

[B116] Nishimura R. Shirasaki T. Tsuchiya K. Miyake Y. Watanabe Y. Hibiya S. (2019). Establishment of a system to evaluate the therapeutic effect and the dynamics of an investigational drug on ulcerative colitis using human colonic organoids. J. Gastroenterol. 54, 608–620. 10.1007/s00535-018-01540-y 30599053

[B117] Nissen L. Casciano F. Gianotti A. (2020). Intestinal fermentation *in vitro* models to study food-induced gut microbiota shift: an updated review. FEMS Microbiol. Lett. 367, fnaa097. 10.1093/femsle/fnaa097 32510557

[B118] Nizet A. (1975). The isolated perfused kidney: possibilities, limitations and results. Kidney Int. 7, 1–11. 10.1038/ki.1975.1 236405

[B119] Novak R. Ingram M. Marquez S. Das D. Delahanty A. Herland A. (2020). Robotic fluidic coupling and interrogation of multiple vascularized organ chips. Nat. Biomed. Eng. 4, 407–420. 10.1038/s41551-019-0497-x 31988458 PMC8057865

[B120] Ohkura T. Ohta K. Nagao T. Kusumoto K. Koeda A. Ueda T. (2014). Evaluation of human hepatocytes cultured by three-dimensional spheroid systems for drug metabolism. Drug Metab. Pharmacokinet. 29, 373–378. 10.2133/dmpk.dmpk-13-rg-105 24695277

[B121] Özkan A. Stolley D. L. Cressman E. N. K. McMillin M. Yankeelov T. E. Rylander M. N. (2023). Vascularized hepatocellular carcinoma on a chip to control chemoresistance through cirrhosis, inflammation and metabolic activity. Small Struct. 4, 2200403. 10.1002/sstr.202200403 38073766 PMC10707486

[B122] Palma E. Doornebal E. J. Chokshi S. (2019). 'Precision-cut liver slices: a versatile tool to advance liver research. Hepatol. Int. 13, 51–57. 10.1007/s12072-018-9913-7 30515676 PMC6513823

[B123] Pan F. Han L. Zhang Y. Yu Y. Liu J. (2015). Optimization of Caco-2 and HT29 co-culture *in vitro* cell models for permeability studies. Int. J. Food Sci. Nutr. 66, 680–685. 10.3109/09637486.2015.1077792 26299896

[B124] Parikh A. Gillam E. M. Guengerich F. P. (1997). Drug metabolism by *Escherichia coli* expressing human cytochromes P450. Nat. Biotechnol. 15, 784–788. 10.1038/nbt0897-784 9255795

[B125] Park E. Kim H. K. Jee J. Hahn S. Jeong S. Yoo J. (2019). Development of organoid-based drug metabolism model. Toxicol. Appl. Pharmacol. 385, 114790. 10.1016/j.taap.2019.114790 31678242

[B126] Park J. C. Jang S. Y. Lee D. Lee J. Kang U. Chang H. (2021). A logical network-based drug-screening platform for Alzheimer's disease representing pathological features of human brain organoids. Nat. Commun. 12, 280. 10.1038/s41467-020-20440-5 33436582 PMC7804132

[B127] Park T. E. Mustafaoglu N. Herland A. Hasselkus R. Mannix R. FitzGerald E. A. (2019). Hypoxia-enhanced blood-brain barrier chip recapitulates human barrier function and shuttling of drugs and antibodies. Nat. Commun. 10, 2621. 10.1038/s41467-019-10588-0 31197168 PMC6565686

[B128] Pastan I. Gottesman M. M. Ueda K. Lovelace E. Rutherford A. V. Willingham M. C. (1988). A retrovirus carrying an MDR1 cDNA confers multidrug resistance and polarized expression of P-glycoprotein in MDCK cells. Proc. Natl. Acad. Sci. U. S. A. 85, 4486–4490. 10.1073/pnas.85.12.4486 2898143 PMC280455

[B129] Peeters L. Vervliet P. Foubert K. Hermans N. Pieters L. Covaci A. (2020). A comparative study on the *in vitro* biotransformation of medicagenic acid using human liver microsomes and S9 fractions. Chem. Biol. Interact. 328, 109192. 10.1016/j.cbi.2020.109192 32712081

[B130] Peng C. Wang C. Lin N. Grill W. M. (2008). Improved bladder emptying in urinary retention by electrical stimulation of pudendal afferents. J. Neural Eng., 2008 Acad. Seminar Clin. Chin. Med., 5. 144, 154. 10.1088/1741-2560/5/2/005 PMC3656418 18430976

[B131] Petrosyan A. Cravedi P. Villani V. Angeletti A. Manrique J. Renieri A. (2019). A glomerulus-on-a-chip to recapitulate the human glomerular filtration barrier. Nat. Commun. 10, 3656. 10.1038/s41467-019-11577-z 31409793 PMC6692336

[B132] Polli J. W. Wring S. A. Humphreys J. E. Huang L. Morgan J. B. Webster L. O. (2001). Rational use of *in vitro* P-glycoprotein assays in drug discovery. J. Pharmacol. Exp. Ther. 299, 620–628. 10.1016/s0022-3565(24)29270-3 11602674

[B133] Qi D. Lin H. Hu B. Wei Y. (2023). A review on *in vitro* model of the blood-brain barrier (BBB) based on hCMEC/D3 cells. J. Control Release 358, 78–97. 10.1016/j.jconrel.2023.04.020 37076016

[B134] Qian W. Gong G. Su H. Zhao Y. Fu W. Wang Y. (2023). Hepar-on-a-sensor-platform with hybridization chain reaction amplification strategy to intuitively monitor the hepatoxicity of natural compounds. Acta Biomater. 160, 73–86. 10.1016/j.actbio.2023.02.021 36804823

[B135] Qian Z. M. Wen X. D. Li H. J. Liu Y. Qin S. J. Li P. (2008). Analysis of interaction property of bioactive components in flos Lonicerae japonicae with protein by microdialysis coupled with HPLC-DAD-MS. Biol. Pharm. Bull. 31, 126–130. 10.1248/bpb.31.126 18175954

[B136] Qiang Li (2011). “Study on the interaction between flavonoids and serum albumin,” in Master's thesis. Nanchang University.

[B137] Rumsey J. W. Lorance C. Jackson M. Sasserath T. McAleer C. W. Long C. J. (2022). Classical Complement Pathway Inhibition in a “Human-On-A-Chip” model of autoimmune demyelinating Nnuropathies. Adv. Ther. (Weinh) 5, 2200030. 10.1002/adtp.202200030 36211621 PMC9540753

[B138] Sabbagh M. F. Nathans J. (2020). A genome-wide view of the de-differentiation of central nervous system endothelial cells in culture. Elife 9, e51276. 10.7554/eLife.51276 31913116 PMC6948952

[B139] Sarvestani S. K. Signs S. Hu B. Yeu Y. Feng H. Ni Y. (2021). Induced organoids derived from patients with ulcerative colitis recapitulate colitic reactivity. Nat. Commun. 12, 262. 10.1038/s41467-020-20351-5 33431859 PMC7801686

[B140] Sateesh J. Guha K. Dutta A. Sengupta P. Yalamanchili D. Donepudi N. S. (2022). A comprehensive review on advancements in tissue engineering and microfluidics toward kidney-on-chip. Biomicrofluidics 16, 041501. 10.1063/5.0087852 35992641 PMC9385224

[B141] Scherbl D. Muentnich S. Richling E. (2014). *In vitro* absorption studies of chlorogenic acids from coffee using the ussing chamber model. Food Res. Int. 63, 456–463. 10.1016/j.foodres.2014.03.031

[B142] Scholz S. Lewis K. Saulich F. Endres M. Boehmerle W. Huehnchen P. (2022). Induced pluripotent stem cell-derived brain organoids as potential human model system for chemotherapy induced CNS toxicity. Front. Mol. Biosci. 9, 1006497. 10.3389/fmolb.2022.1006497 36188215 PMC9520921

[B143] Schutgens F. Clevers H. (2020). Human organoids: tools for understanding biology and treating diseases. Annu. Rev. Pathol. 15, 211–234. 10.1146/annurev-pathmechdis-012419-032611 31550983

[B144] Schyschka L. Sánchez J. J. Wang Z. Burkhardt B. Müller-Vieira U. Zeilinger K. (2013). Hepatic 3D cultures but not 2D cultures preserve specific transporter activity for acetaminophen-induced hepatotoxicity. Arch. Toxicol. 87, 1581–1593. 10.1007/s00204-013-1080-y 23728527

[B145] Sciancalepore A. G. Sallustio F. Girardo S. Gioia Passione L. Camposeo A. Mele E. (2014). 'A bioartificial renal tubule device embedding human renal stem/progenitor cells. PLoS One 9, e87496. 10.1371/journal.pone.0087496 24498117 PMC3907467

[B146] Seto Y. Inoue R. Ochi M. Gandy G. Yamada S. Onoue S. (2011). Combined use of *in vitro* phototoxic assessments and cassette dosing pharmacokinetic study for phototoxicity characterization of fluoroquinolones. Aaps J. 13, 482–492. 10.1208/s12248-011-9292-7 21739333 PMC3160149

[B147] Seto Y. Ohtake H. Kato M. Onoue S. (2015). Phototoxic risk assessments on benzophenone derivatives: photobiochemical assessments and dermal cassette-dosing pharmacokinetic study. J. Pharmacol. Exp. Ther. 354, 195–202. 10.1124/jpet.115.223644 26016852

[B148] Shah P. Fritz J. V. Glaab E. Desai M. S. Greenhalgh K. Frachet A. (2016). A microfluidics-based *in vitro* model of the gastrointestinal human-microbe interface. Nat. Commun. 7, 11535. 10.1038/ncomms11535 27168102 PMC4865890

[B149] Shi Y. He X. Wang H. Dai J. Fang J. He Y. (2023). Construction of a novel blood brain barrier-glioma microfluidic chip model: applications in the evaluation of permeability and anti-glioma activity of traditional Chinese medicine components. Talanta 253, 123971. 10.1016/j.talanta.2022.123971 36201955

[B150] Shin W. Kim H. J. (2018). Intestinal barrier dysfunction orchestrates the onset of inflammatory host-microbiome cross-talk in a human gut inflammation-on-a-chip. Proc. Natl. Acad. Sci. U. S. A. 115, E10539–E10547. 10.1073/pnas.1810819115 30348765 PMC6233106

[B151] Shinozawa T. Kimura M. Cai Y. Saiki N. Yoneyama Y. Ouchi R. (2021). High-fidelity drug-induced liver injury screen using human pluripotent stem cell-derived organoids. Gastroenterology 160, 831–46.e10. 10.1053/j.gastro.2020.10.002 33039464 PMC7878295

[B152] Skardal A. Devarasetty M. Kang H. W. Seol Y. J. Forsythe S. D. Bishop C. (2016). Bioprinting cellularized constructs using a tissue-specific hydrogel bioink. J. Vis. Exp., e53606. 10.3791/53606 27166839 PMC4941985

[B153] Smyth L. C. D. Rustenhoven J. Scotter E. L. Schweder P. Faull R. L. M. Park T. I. H. (2018). Markers for human brain pericytes and smooth muscle cells. J. Chem. Neuroanat. 92, 48–60. 10.1016/j.jchemneu.2018.06.001 29885791

[B154] Song C. Han X. Guo D. (2017). Application of liver microsomes *in vitro* metabolism in bio-transformation of traditional Chinese medicine. J. Chengdu Univ. Tradit. Chin. Med. 40, 115–118. 10.13593/j.cnki.51-1501/r.2017.02.115

[B155] Song S. Bai Y. Liang J. (2023). Application progress of parallel artificial membrane permeation assay model in drug permeability screening of transdermal drug delivery system. Chin. Pharm. 34, 502–507. 10.6039/j.issn.1001-0408.2023.04.23

[B156] Sun L. Li Y. Liu X. Jin M. Zhang L. Du Z. (2011). Cytotoxicity and mitochondrial damage caused by silica nanoparticles. Toxicol Vitro 25, 1619–1629. 10.1016/j.tiv.2011.06.012 21723938

[B157] Taft D. R. (2004). The isolated perfused rat kidney model: a useful tool for drug discovery and development. Curr. Drug Discov. Technol. 1, 97–111. 10.2174/1570163043484824 16472223

[B250] Tang L. Ye L. Lv C. Zheng Z. Gong Y. Liu Z. (2011). ‘Involvement of CYP3A4/5 and CYP2D6 in the metabolism of aconitine using human liver microsomes and recombinant CYP450 enzymes’. Toxicol Lett. 202, 47–54. 10.1016/j.toxlet.2011.01.019 21277363

[B158] Tian K. Huang Z. Wang X. Geng X. Li B. Lu J. (2020). Research progress on *in vitro* models for evaluating drug-induced neurotoxicity. Drug Eval. Res. 43, 1433–1438. 10.7501/j.issn.1674-6376.2020.07.043

[B159] Tontsch U. Bauer H. C. (1991). Glial cells and neurons induce blood-brain barrier related enzymes in cultured cerebral endothelial cells. Brain Res. 539, 247–253. 10.1016/0006-8993(91)91628-e 1675906

[B160] Tostões R. M. Leite S. B. Serra M. Jensen J. Björquist P. Carrondo M. J. (2012). Human liver cell spheroids in extended perfusion bioreactor culture for repeated-dose drug testing. Hepatology 55, 1227–1236. 10.1002/hep.24760 22031499

[B161] Ugolev A. M. Bagiian A. A. Ekkert L. G. (1980). New method of studying membrane hydrolysis and transport as well as metabolic processes in the small intestine *in vitro* (everted small intestinal sac with bilateral oxygenation). Fiziol. Zh SSSR Im. I M. Sechenova 66, 1674–1677. 7439457

[B162] Vatine G. D. Al-Ahmad A. Barriga B. K. Svendsen S. Salim A. Garcia L. (2017). Modeling psychomotor retardation using iPSCs from MCT8-Deficient patients indicates a prominent role for the blood-brain barrier. Cell Stem Cell 20, 831–43.e5. 10.1016/j.stem.2017.04.002 28526555 PMC6659720

[B163] Veronesi B. (1996). Characterization of the MDCK cell line for screening neurotoxicants. Neurotoxicology 17, 433–443. 8856739

[B164] Wadman M. (2023). FDA no longer has to require animal testing for new drugs. SCIENCE 379, 127–128. 10.1126/science.adg6276 36634170

[B165] Wang D. Liu R. Zeng J. Li C. Xiang W. Zhong G. (2022). Preliminary screening of the potential active ingredients in traditional Chinese medicines using the ussing chamber model combined with HPLC-PDA-MS. J. Chromatogr. B Anal. Technol. Biomed. Life Sci. 1189, 123090. 10.1016/j.jchromb.2021.123090 34959037

[B166] Wang G. Y. Wang N. Liao H. N. (2015). Effects of muscone on the expression of P-gp, MMP-9 on blood-brain barrier model *in vitro* . Cell Mol. Neurobiol. 35, 1105–1115. 10.1007/s10571-015-0204-8 25976179 PMC11488062

[B167] Wang H. Ning X. Zhao F. Zhao H. Li D. (2024a). Human organoids-on-chips for biomedical research and applications. Theranostics 14, 788–818. 10.7150/thno.90492 38169573 PMC10758054

[B168] Wang H. Zhang S. Jin Y. Cao T. Qin Q. Liu W. (2024b). Study on the mechanism of hepatotoxicity induced by rhubarb based on network pharmacology and experimental verification. WST-Mod Tradit. Chin. Med. Mater Med. 26, 167–178. 10.7150/thno.90492

[B169] Wang Q. Kuang Y. Song W. Qian Y. Qiao X. Guo D. A. (2017a). Permeability through the Caco-2 cell monolayer of 42 bioactive compounds in the TCM formula gegen-qinlian decoction by liquid chromatography tandem mass spectrometry analysis. J. Pharm. Biomed. Anal. 146, 206–213. 10.1016/j.jpba.2017.08.042 28886521

[B170] Wang R. Ding R. Liu Z. (2017b). “A study on the absorption and transport mechanism of aconite alkaloids in gancao fuzi decoction based on triple Quadrupole tandem mass spectrometry technology,” in The 3rd national mass spectrometry analysis academic conference, 1. Xiamen, Fujian, China.

[B171] Wang X. He B. Shi J. Li Q. Zhu H. J. (2020). Comparative proteomics analysis of human liver microsomes and S9 fractions. Drug Metab. Dispos. 48, 31–40. 10.1124/dmd.119.089235 31699809 PMC6918043

[B172] Wang J J. Feng X. Dang W. Li Z Liu Z. Pi J. (2023). A study on the absorption characteristics of baicalin-berberine complex and nanocrystals in a Caco-2 cells monolayer model. J. Tianjin Univ. Tradit. Chin. Med. 42, 87–94. 10.11656/j.issn.1673-9043.2023.01.17

[B173] Wang X X. Quan J. Xiu C. Wang J. Zhang J. (2023). Gegen Qinlian decoction (GQD) inhibits ulcerative colitis by modulating ferroptosis-dependent pathway in mice and organoids. Chin. Med. 18, 110. 10.1186/s13020-023-00819-4 37649073 PMC10466729

[B174] Weber E. J. Lidberg K. A. Wang L. Bammler T. K. MacDonald J. W. Li M. J. (2018). Human kidney on a chip assessment of polymyxin antibiotic nephrotoxicity. JCI Insight 3, e123673. 10.1172/jci.insight.123673 30568031 PMC6338315

[B175] Wen X. D. Qi L. W. Chen J. Song Y. Yi L. Yang X. W. (2007). Analysis of interaction property of bioactive components in danggui buxue decoction with protein by microdialysis coupled with HPLC-DAD-MS. J. Chromatogr. B Anal. Technol. Biomed. Life Sci. 852, 598–604. 10.1016/j.jchromb.2007.02.041 17383242

[B176] Westerhout J. Wortelboer H. Verhoeckx K. (2015). “Ussing chamber,” in The impact of food bioactives on health: *in vitro* and *ex vivo* models. Editors Verhoeckx K. Cotter P. López-Expósito I. Kleiveland C. Lea T. Mackie A. (Springer). 29787039

[B177] Wieser M. Stadler G. Jennings P. Streubel B. Pfaller W. Ambros P. (2008). hTERT alone immortalizes epithelial cells of renal proximal tubules without changing their functional characteristics. Am. J. Physiol. Ren. Physiol. 295, F1365–F1375. 10.1152/ajprenal.90405.2008 18715936

[B178] Wilmer M. J. Ng C. P. Lanz H. L. Vulto P. Suter-Dick L. Masereeuw R. (2016). Kidney-on-a-Chip technology for drug-induced nephrotoxicity screening. Trends Biotechnol. 34, 156–170. 10.1016/j.tibtech.2015.11.001 26708346

[B179] Wilson T. H. Wiseman G. (1954). The use of sacs of everted small intestine for the study of the transference of substances from the mucosal to the serosal surface. J. Physiol. 123, 116–125. 10.1113/jphysiol.1954.sp005036 13131249 PMC1366157

[B180] Wu H. Wang H. Wang L. Luo X. Zou D. (2024). Application progress and challenges of artificial intelligence in organoid research. CHINA Oncol. 34, 210–219. 10.19401/j.cnki.1007-3639.2024.02.009

[B181] Xi J. Wang S. Zhao X. Zhang F. Zhai D. Zhao Y. (2008). Protection of glycyrrhetinic acid and matrine against experimental acute vanishing bile duct syndrome. Chin. J. Pharmacol. Toxicol., 49–54.

[B182] Xiang Y. Tanaka Y. Patterson B. Kang Y. J. Govindaiah G. Roselaar N. (2017). Fusion of regionally specified hPSC-Derived organoids models human brain development and interneuron migration. Cell Stem Cell 21, 383–98.e7. 10.1016/j.stem.2017.07.007 28757360 PMC5720381

[B183] Xicoy H. Wieringa B. Martens G. J. (2017). The SH-SY5Y cell line in parkinson's disease research: a systematic review. Mol. Neurodegener. 12, 10. 10.1186/s13024-017-0149-0 28118852 PMC5259880

[B184] Xu T. Wu Z. Yao H. Zhang Y. Chen S. Li Y. (2024). Evaluation of aconitine cardiotoxicity with a heart-on-a-particle prepared by a microfluidic device. Chem. Commun. (Camb) 60, 4898–4901. 10.1039/d4cc00396a 38629248

[B185] Yeggoni D. Kuehne C. Rachamallu A. Subramanyam R. (2017). Elucidating the binding interaction of andrographolide with the plasma proteins: biophysical and computational approach. RSC Adv. 7, 5002–5012. 10.1039/c6ra25671f

[B186] Yin P. Wang X. (2024). Progresses in the establishment, evaluation, and application of *in vitro* blood-brain barrier models. J. Neurosci. Res. 102, e25359. 10.1002/jnr.25359 38859680

[B187] Yu L. Bi H. Wu B. Ge G. Zheng J. Qiao H. (2019). 2018 DMPK research progress in China, 54. Elsevier B.V., 963–970.

[B188] Yuzhuo He (2006). “A survey of research on “serum pharmacology” and “serum pharmacochemistry” of Japanese traditional Chinese medicines,” in A compilation of references for the 11th academic seminar on the regulating mechanism of acupuncture and moxibustion on body functions and the unique clinical experience of acupuncture and moxibustion (Shanghai, China), 5.

[B189] Zhang D. Luo G. Ding X. Lu C. (2012). Preclinical experimental models of drug metabolism and disposition in drug discovery and development. Acta Pharm. Sin. B 2, 549–561. 10.1016/j.apsb.2012.10.004

[B190] Zhang D. Raghavan N. Chen S. Y. Zhang H. Quan M. Lecureux L. (2008). Reductive isoxazole ring opening of the anticoagulant razaxaban is the major metabolic clearance pathway in rats and dogs. Drug Metab. Dispos. 36, 303–315. 10.1124/dmd.107.018416 17984286

[B191] Zhang M. Zhang Y. Q. Wei X. Z. Lee C. Huo D. S. Wang H. (2019). Differentially expressed long-chain noncoding RNAs in human neuroblastoma cell line (SH-SY5Y): alzheimer's disease cell model. J. Toxicol. Environ. Health A 82, 1052–1060. 10.1080/15287394.2019.1687183 31722651

[B192] Zhang Q. Zheng H. Zhao C. Song Y. Yang Q. Sun J. (2022). Research progress of interaction between the metabolism of active ingredients of traditional Chinese medicine and intestinal flora. NW Pharm. J. 37, 186–188.

[B193] Zhang W. Wang X. Huang A. Xie D. Xin M. Liu W. (2023). Research status and application prospect of organ-on-chip technology in the field of drug toxicology. World Clin. Drug 44, 83–89. 10.13683/j.wph.2023.01.014

[B194] Zhao X. Bai Z. Zhan X. Wang J. Cheng Y. Xiao X. (2024). Safety evaluation of traditional Chinese medicine: new era, new strategy. AHM 4, 171–175. 10.1097/hm9.0000000000000119

[B195] Zhao Y. Zhang Y. Xu L. Chen X. Yu Q. (2007). Protective effect of glycyrrhizin acid,glycyrrhetinic acid and matrine on acute cholestasis induced by alpha-naphthylisothiocyanate in rats. J. Chin. Pharm. Univ., 256–260. 10.1055/s-2006-957067

[B196] Zhao Z. Nelson A. R. Betsholtz C. Zlokovic B. V. (2015). Establishment and dysfunction of the blood-brain barrier. Cell 163, 1064–1078. 10.1016/j.cell.2015.10.067 26590417 PMC4655822

[B197] Zheng L. Wan H. Zhou H. Zhang Y. Yang J. Wan H. (2015). Transport characteristics of flavonoids in buyang huanwu decoction across Caco-2 cell monolayer model and their effect on cytochrome P450 activity. J. Anhui Univ. Chin. Med. 34, 68–74.

[B198] Zhu L. Du H. He Y. Yu M. Zhang D. Yang J. (2021). Reevaluation of liver safety of commonly used Chinese medicine injection based on bio-printing 3D cell microfluidic chip. Chung-nan Yao Hsueh 19, 2304–2310.

[B199] Zhu Y. Zhang S. Hu Z. Liu H. Sun J. Hao K. (2024). Retrospect and prospect of research methodology for pharmacokinetic interactions between traditional Chinese and Western medicine. Chin. Tradit. Herb. Drugs 55, 1788–1798. 10.7501/j.issn.0253-2670.2024.06.002

[B200] Zietek T. Giesbertz P. Ewers M. Reichart F. Weinmüller M. Urbauer E. (2020). Organoids to study intestinal nutrient transport, drug uptake and metabolism - update to the human model and expansion of applications. Front. Bioeng. Biotechnol. 8, 577656. 10.3389/fbioe.2020.577656 33015026 PMC7516017

[B201] Zou L. Wang Y. Jiang Z. Ju S. Zhang B. Lin Z. (2021). Screening and evaluation of active ingredients of traditional Chinese medicine for lowering uric acid based on renal uric acid transport:a case study of cichoric acid. World Chin. Med. 16, 28–34. 10.3969/j.issn.1673-7202.2021.01.005

