# Supplementary material for: In vitro technology and ADMET research in traditional Chinese medicine
Source: Front Pharmacol. 2025 Jul 9;16:1605330. doi: 10.3389/fphar.2025.1605330 (PMC12283783; doi:10.3389/fphar.2025.1605330)
Supplement: Supplementary file 1 [file DataSheet1.docx]

Supplementary Material

*for*

*In vitro technology and ADMET research in traditional Chinese medicine*

**Supplementary Text1. Bibliometric analysis**

**1.1 Mothed**

First, we searched the hot spots and progress of *in vitro* models in the research of traditional Chinese medicine on the Web of Science and CNKI. A total of 3776 articles were retrieved from the core database of Web of Science, and 65 articles were retrieved from CNKI. The criteria for inclusion in the study were as follows: (1) Inclusion of Web of Science Core Collection and CNKI (core journals; CSCD; WJCI;) research, including reviews and original articles. (3) Main objects: Study on the effective components of traditional Chinese medicine and its compound prescription. (4) Research methods: These methods focus mainly on *in vitro* models, which are widely used in the study of ADMET (absorption, distribution, metabolism, excretion and toxicity). (5) The term is from 2001--2024. After manual screening, 60 Chinese documents and 405 English documents were obtained. The bibliometric analysis and visualization were subsequently carried out via Vosviewer 1.6.20 software (van ECK et and Waltman, 2010) . After the keywords are exported, they are replaced with replacement files and reimported into VOSviewer for analysis.

**1.2 Discussion of the results of the literature analysis**

In the bibliometric data (Supplementary Figure 1), high-frequency keywords show that "metabolism", "toxicity" and "absorption" occupy a dominant position, reflecting that the current TCM research focuses on the metabolic transformation mechanism and safety evaluation of components. However, the literature on "distribution" and "excretion" is insufficient, which highlights the shortcomings of systematic analysis of the dynamic process of traditional Chinese medicine. In the traditional model, the Caco-2 cell model reveals the permeation law of components by simulating the intestinal epithelial barrier, and the liver microsomal metabolism model dominated by the cytochrome P450 enzyme system explains the first-pass effect and metabolic activation toxicity. Notably, the P-gp glycoprotein is the core transporter. The high frequency of P-gp shows that TCM focuses on research on intestinal absorption limitations, the regulation of hepatobiliary excretion and multidrug resistance mechanisms, and the risk of clinical combinations caused by the competitive transport of chemical drugs. In addition, the keywords "drug interaction" and "pharmacokinetics" further reveal the possible clinical risks when TCM is used in combination with chemical drugs and the need to simulate the process of TCM *in vivo*. However, existing technology systems are limited mostly to the linear research mode of a single link and a single target. It is difficult to adapt to the complex characteristics of multicomponent synergy and dynamic distribution across organs of TCMs, especially in the study of the overall effect of TCM compounds and the dynamic balance between organs. Therefore, research on ADMET should focus on the development and application of new *in vitro* models, such as organ-like chips and 3D cocultures. These new models combine advanced means of multiomics technology and artificial intelligence to achieve the systematic evaluation of the synergistic effect and overall pharmacokinetic behavior of TCM compounds.


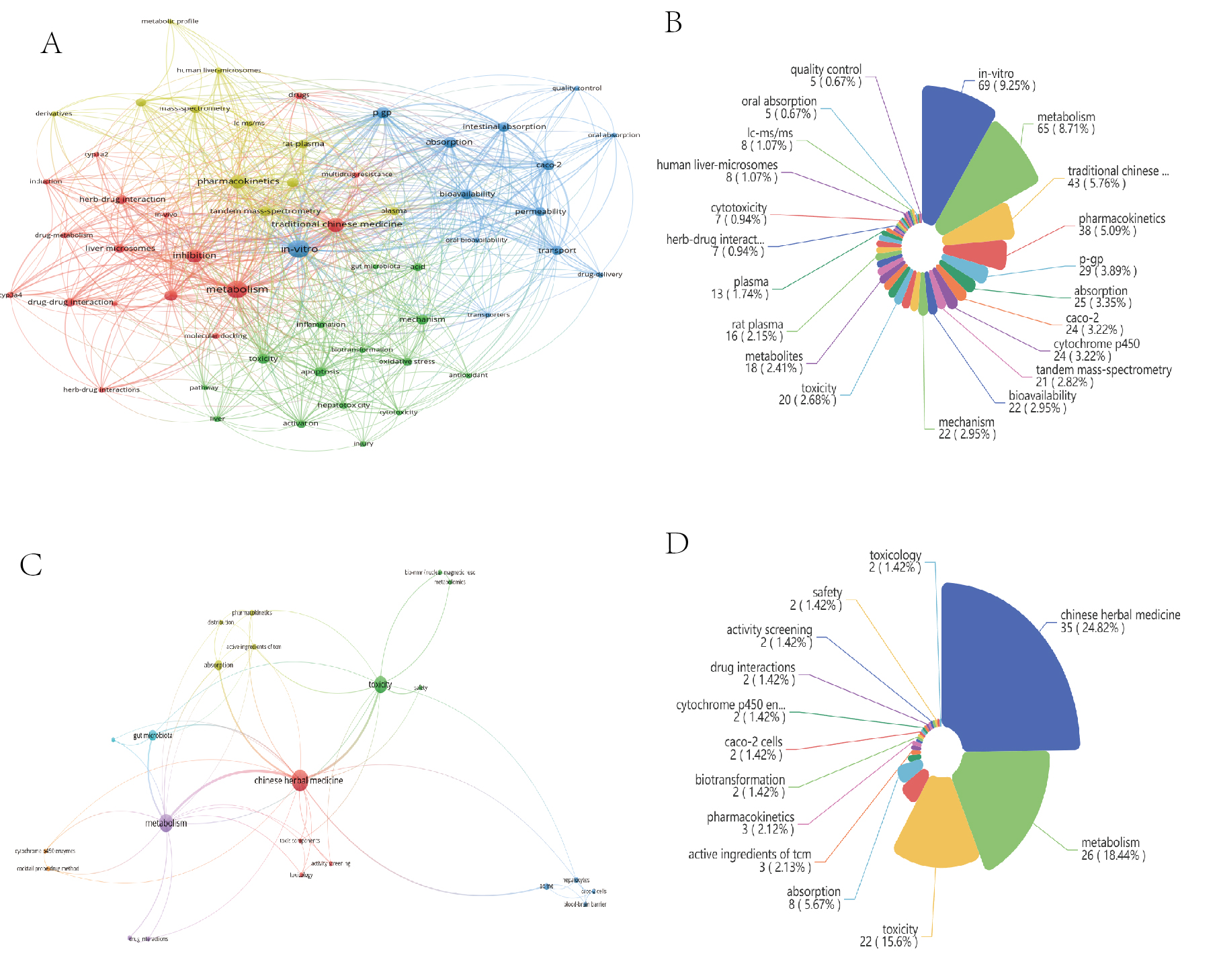


**Supplementary Figure 1.** Annual number of articles about cuttlebone published in the WOS database (Web of Science Core Collection) and the CNKI database (core journals, WJCI, CSCD). A keyword co-occurrence network of Chinese journal articles (those with >5 keywords). B Nightingale rose charts of keyword frequency distributions in English journal articles (appearing >5). C Keyword co-occurrence network of Chinese journal articles (appearing >2). D Nightingale rose charts of keyword frequency distributions in Chinese journal articles (appearing >2).

**Supplementary Text2. Bibliometric analysis**

**2.1 Combination and Optimization of *In Vitro* Models**

In constructing an *in vitro* ADMET evaluation system for drug development, the scientific and effective combination of multiple models is crucial, requiring systematic consideration of the entire process—including absorption, distribution, metabolism, excretion, and toxicity. For drug absorption studies, the most physiologically relevant intestinal *in vitro* models should incorporate key components such as epithelial cells, a mucus layer, co-culture systems, transport proteins, buffers, and scaffolding/support systems. The traditional Caco-2 cell monolayer model, while widely used, lacks a genuine mucus layer, limiting its ability to simulate the actual intestinal absorption environment. To address this deficiency, the co-culture system of HT29-MTX and Caco-2 cells is widely applied, effectively reconstructing the cooperative barrier of intestinal epithelium and mucus, thus enhancing the physiological relevance of drug-microenvironment interactions. Furthermore, the everted gut sac model can differentiate and simulate absorption differences across various intestinal segments (such as the duodenum, jejunum, and ileum), thereby supplementing and refining the overall assessment of drug absorption throughout the gut. It is important to note that macromolecular drugs often possess large molecular weight, hydrophilicity, variable solubility, limited chemical stability, and are susceptible to gastrointestinal degradation; consequently, the current *in vitro* absorption models based on intestinal structure are generally inadequate for evaluating the absorption of traditional Chinese medicines and other macromolecular drugs.

In the evaluation of drug distribution, the combined use of plasma protein binding models and blood-brain barrier models can comprehensively reveal the free and bound states of drugs in the bloodstream, as well as predict their ability to penetrate into the central nervous system. Additionally, combining these with *in vivo* tissue distribution studies provides systematic analysis of the final targeting and exposure of drugs across various organs and tissues. Plasma protein binding determines the effective concentration of free drug, while the blood-brain barrier limits central exposure; tissue distribution analysis provides crucial supplementary information for evaluating drug targeting.

For metabolism studies, a multi-tiered combination of hepatic models has become mainstream. Hepatic microsomes, S9 fractions, and cytosolic fractions are used for high-throughput enzyme kinetics and metabolite screening; primary hepatocytes and differentiated stem cells are suitable for studying individual differences and complex metabolic products; tumor-derived hepatic cell lines are appropriate for mechanistic and high-throughput screening studies. Recombinant enzyme systems (such as CYP, UGT, etc.) can precisely identify specific metabolizing enzymes, while cellular or primary hepatocyte models can reveal coordinated enzyme actions. In recent years, the combined use of hepatic models and gut microbiota models has become increasingly common, as the gut microbiota plays a critical role in the first-pass metabolism and bioactivation of certain drugs. The integration of these models not only more closely mirrors *in vivo* metabolic pathways, but also elucidates the cooperative mechanisms of the gut-liver axis in drug metabolism and efficacy/toxicity, which is especially significant for complex natural products and traditional Chinese medicines.

Regarding toxicity assessment, traditional approaches often depend on a single hepatic cell line (such as HepG2 or L02) or renal, cardiac, and other cell types, yet these models rarely reflect the multi-organ and multi-level nature of toxicity responses in humans. Modern evaluation systems advocate the combined application of normal human-derived cells (L02, IMR-90, PHHs, etc.), tumor cells (HepG2, MCF-7, Huh7, etc.), and organ-specific cell types (kidney, heart, nervous system, etc.), together with multi-parameter assays such as cell viability, oxidative stress (ROS), mitochondrial membrane potential, and cellular morphology. This multi-faceted approach enables comprehensive profiling of organ-specific toxicity and sensitivities.

With the development of 3D organoids and organ-on-a-chip technologies, researchers can more accurately reconstruct the intestinal epithelium, mucus layer, and co-culture environments. Through the use of microfluidic scaffolds and multi-organ chips (such as gut-liver-kidney-brain multi-organ chips), the physiological relevance and dynamic simulation capabilities of these models are greatly enhanced. Such organ-on-chip systems not only permit long-term culture and dynamic observation but also efficiently simulate complex interactions among physiological barriers, transporters, and enzyme systems, greatly expanding the applicability of drug evaluation models. Altogether, these advances markedly enhance the predictive power, relevance, and mechanistic interpretability of *in vitro* ADMET evaluation, providing a solid and forward-looking scientific foundation for the research and development of complex drugs, natural products, and macromolecular drugs.

**Supplementary 1. Principles, Advantages, and Disadvantages of Absorption, Distribution, and Metabolism Models**

|  | Principles | Advantages | Disadvantages | | references |  |
| --- | --- | --- | --- | --- | --- | --- |
| absorption models *in vitro* | | | | | |  |
| Parallel artificial membrane Penetration Test (PAMPA) | Mainly evaluate the passive diffusion ability of drugs, by measuring the penetration of drugs on both sides of artificial membrane. | It can be used for the determination of Qualcomm, with low cost, good flexibility and convenient and rapid detection. | Transporter-mediated transport cannot be studied because the PAMPA model does not include cells and transporters. | | (Song et al. 2023b; Wu et al. 2011; Song Sankong et al. 2023; Shen et al. 2018) |  |
| MDCK cell model | The absorption efficiency of drugs was evaluated by measuring the concentration changes of drugs on both sides of cell monolayer. | High expression of human P-gp, existence of multivitamin transport vector, short culture time and higher expression of P-gp than Caco-2. Passive diffusion and active transport can be distinguished, and different absorption mechanisms can be analysed by adding specific inhibitors. | Only intestinal epithelial cell transport can be studied. Multicellular layer phenomenon may appear after transfection. | | (Liu et al. 2008; Shen et al. 2018) |  |
| Ussing Chamber | Ussing Chamber model simulates the absorption environment *in vivo* by fixing intestinal tissue (usually small intestine or colon) in perfusion chambers on both sides. During the experiment, the concentration changes of drugs in intestinal lumen side (lumen) and vascular side (external lumen) can be measured, respectively. | By changing the composition of perfusion solution (such as using different ions and pH values) or adding specific absorption inhibitors, we can study whether drugs are absorbed through passive diffusion, active transport or other mechanisms. The Ussing Chamber model can study the interaction between drugs and specific transporters by adding specific transporter inhibitors or gene knock-down techniques. | Only water molecules and ions are allowed to penetrate, but macromolecules are difficult to penetrate. | | (Shen et al. 2018; Chen et al. 2016) |  |
| Intestinal organ | Intestinal absorption was simulated by organ-like cavity structure, and barrier permeability was evaluated by fluorescent probe labelling (such as FITC-dextran) | Highly preserve the three-dimensional structure and cell diversity of the intestine (stem cells, goblet cells, endocrine cells, etc.); Support long-term cultivation (> 1 month); It can simulate the polarity differentiation of intestinal crypt-villus; Patient-specific modelling (such as disease organs) | Long construction period (2-4 weeks); High heterogeneity between batches; Lack of dynamic mechanical stimulation (such as peristalsis); Qualcomm screening is difficult. | | (Pleguezuelos-Manzano et al. 2020; Xiang et al. 2024) |  |
| *In vitro* model of the plasma protein binding | | | |  |  |  |
| Equilibrium Dialysis | Equilibrium dialysis uses a semipermeable membrane to separate protein/drug chambers, measuring equilibrium concentrations of both free and bound drug without separation. | There is no need to separate free drugs from bound drugs, so it will not affect the balance of binding. | Dilution effects may lower apparent binding; time-consuming (risk for hydrolyzable drugs); inaccurate for supersaturated drugs at therapeutic concentrations. | | (Barré et al. 1985; Liu et al. 2007) |  |
| Utrafiltration Method | Ultrafiltration centrifugation separates free drug: Protein-bound complexes are retained in the upper chamber while free drug passes into the | It is close to balanced dialysis in clinical application. The measurement time is short, which is suitable for rapid evaluation of drug binding characteristics. It can be widely used to analyse the concentration of free drugs in large-scale biological samples. | It may be affected by nonspecific adsorption, especially at high concentration. High-quality filter membranes are needed to avoid interfering with the results. | | (Sophianopoulos et al. 1978; Banker et al. 2003; Fung et al. 2003; Liu et al. 2007) |  |
| Microdialysis | Microdialysis is based on semipermeable membrane sampling technology, which diffuses drugs from plasma to dialysate through microdialysis probe. After the drug concentration in the dialysate and the free drug concentration in the plasma reached a dynamic balance, the drug concentration in the dialysate was determined by high-performance liquid chromatography (HPLC) or other analytical methods. | It can be carried out under physiological conditions, and the change of free concentration of drugs can be dynamically monitored in real time, especially suitable for studying the distribution and behavior of drugs in different tissues. | The operation process is complex, which requires the support of professional equipment and personnel, and the experimental period is long, and the cost of equipment and consumables is high. In addition, the semipermeable membrane may have some influence on the diffusion of some drugs, especially for drugs with high protein binding rate, and the equilibrium time may be longer, thus affecting the experimental efficiency. | | (Liu et al. 2007; Wang et al. 2018) |  |
| High efficiency frontier analysis (HPFA) | It adopts a size exclusion chromatographic column with reversed-phase silica gel (ISRP) on the inner surface, which can directly inject samples to realize elution, separation and simultaneous analysis of total drugs and free drugs in biological matrix such as plasma. | Small sample volume and high detection ability | The disadvantage of HPFA is to ensure the reasonable selection of column and packing, and it may not be able to effectively measure the plasma protein binding rate of some compounds with special physical and chemical properties (such as highly hydrophobic or strong acid and alkaline drugs) | | (Chen et al. 2004; Ostergaard et al. 2003; Martínez-Pla et al. 2004; Liu et al. 2007) |  |
| Surface plasmon resonance (SPR) | Drug binding to chip-immobilized proteins alters surface refractive index, shifting the resonance angle. Real-time monitoring of angular/intensity changes enables tracking of binding dynamics and calculation of kinetic parameters. | No fluorescent/radioactive labelling is needed, and the natural activity is maintained; high sensitivity; Real-time monitoring can directly obtain binding kinetic data (such as binding rate and dissociation rate); Low sample consumption; Qualcomm screening | High equipment costs; potential protein conformation/activity alterations during chip immobilization; nonspecific adsorption of plasma components (e.g., lipids) requiring optimized blocking; susceptibility to solution refractive index fluctuations; reduced accuracy for ultra-high/low affinity interactions. | | (Zheng et al. 2021; Lu et al. 2007; Butt 2025) |  |
| Circular Dichroism（CD） | Circular dichroism (CD) detects chiral protein secondary structures (α-helix/β-sheet) via differential absorption of polarized light. Drug-induced conformational changes shift spectral bands, enabling qualitative analysis of binding mode and strength. | Detects minute protein conformational changes for drug-protein interaction studies; preserves sample integrity for reuse; enables rapid high-throughput screening; operates in solution state under physiological-like conditions. | Primarily characterizes protein secondary structures; lacks direct quantification of drug-binding constants; limited in multicomponent/complex conformational analysis; requires expensive instrumentation and demanding experimental conditions. | |  |  |
| Blood–brain barrier cell model *in vitro* | | | | | |  |
| HCMEC/D3 cell model | The hCMEC/D3 cell model is an *in vitro* monolayer of human brain microvascular endothelial cells that expresses various enzymes and transporters, making it useful for studying blood-brain barrier transport and function. | HCMEC/D3 can express many enzymes or transporters, such as cytochrome P450, P-gp, breast cancer resistance protein, etc. | The connection between hCMEC/D3 cells is not tight enough. | | (Weksler et al. 2013; w) |  |
| RBE4 cell model | The RBE4 cell model is a rat brain microvascular endothelial cell line. | It can express almost all the characteristic markers of brain microvascular endothelial cells and be applied to the study of neuroendocrine factors and drug transport mechanism. | *In vivo* experiments or analysis and verification of mRNA/protein expression of freshly isolated RBE4 cells are needed. | | (Veszelka et al. 2018; Faria et al. 2010) |  |
| FungTranswell model of single culture | The single culture Transwell model grows only brain microvascular endothelial cells (BMECs) on a membrane to form a basic selective barrier. | The model is stable, low cost and allows cell migration, and can be used for Qualcomm screening of compounds, combining affinity and transporter dynamics. | Because it only includes one kind of cells, namely, BMEC, and it cannot fully replicate the brain structure and ionic environment. | | (Jagtiani et al. 2022) |  |
| Transwell model of double coculture | The double coculture model adds astrocytes to the lower chamber, which secrete factors promoting endothelial cell maturation and tighter junctions. | Compared with the single cell culture model, the coculture model cocultures astrocytes and cerebral vascular endothelial cells, forming a closer barrier; Compared with the three-culture model, it is easier to establish and use, with good reproducibility and lower cost. | Endothelial cells grow slowly to the phenotype of blood‒brain barrier, and it is difficult to maintain the characteristics of blood‒brain barrier in fully differentiated cells | |  |  |
| Transwell model of triple coculture | The triple coculture model further includes neurons or pericytes. | The three-culture model is to coculture neurons, astrocytes and peripheral cells. Transwell model of three cultures significantly increased the resistance across endothelial cells and the expression of tight junction protein, which was more similar to the *in vivo* environment. | Lack of direct intercellular communication between pericytes, astrocytes and brain microvascular endothelial cells. | |  |  |
| The dynamic *in vitro* model | The dynamic *in vitro* model generates shear stress through continuous perfusion, simulating the hemodynamic environment experienced by vascular endothelial cells *in vivo*. | The model shows high transendothelial resistance (TEER) and low protein leakage, and has good barrier function. The shear stress in blood vessel can be simulated. | It takes a long time to reach steady-state TEER, so it is difficult to study the phenotype and morphology of endothelial cells, and the design and operation of the model are relatively complicated, so it is not suitable for Qualcomm screening. | | (Bussolari et al. 1982; Siddharthan et al. 2007; ) |  |
| cells from different sources in liver research | | | | | |  |
| Primary human liver cells | Isolated from surgically excised or donated human liver tissue by collagenase perfusion method. Drug metabolism research, toxicity evaluation, viral hepatitis model | Retains the natural metabolic enzyme activity and transporter function, which is closest to the physiological state *in vivo* | The donor is scarce, the individual difference is large, the survival time *in vitro* is short (< 7 days), and the cost is high. | | (Bale et al. 2014; Wilkening et al. 2003; Guo et al. 2011; Choucha-Snouber et al. 2013) |  |
| Hepatoma cell lines |  |  |  | |  |  |
| HepG2 cell line | Human hepatocellular carcinoma cell line was passaged by conventional cell culture. Primary screening of drug hepatotoxicity, study on lipid metabolism | Easy to culture, low cost, Qualcomm screening and stable gene expression profile. | CYP450 has low enzyme activity (especially CYP3A4/2D6) and lacks polar structure and functional transporters | | (Guo et al. 2011; Anthérieu et al. 2010) |  |
| JaHepaRG cell line | Dimethyl sulfoxide (DMSO) was needed to induce differentiation (2 weeks) to hepatocyte-like state.Drug metabolism and toxicity research, nonalcoholic fatty liver model | After differentiation, the expression of high level CYP450 enzyme (close to primary hepatocytes) can be used to study bile acid metabolism. | The differentiation process is time-consuming and the metabolic activity is low in undifferentiated state | | (Choucha-Snouber et al. 2013; Anthérieu et al. 2010) |  |
| Adult stem cells |  |  |  | |  |  |
| Liver progenitor cells | Isolation of progenitor cells with bidirectional differentiation potential from fetal or adult liver  Liver regeneration research, chronic liver disease model construction | Can directionally differentiate into hepatocytes or bile duct cells, and has strong proliferation ability. | It is difficult to separate and may lose its differentiation ability after amplification *in vitro* | | (Schwartz et al. 2002; Campard et al. 2008; Snykers et al. 2011) |  |
| Mesenchymal stem cells | Good availability (e.g. from  bone marrow, placenta,  umbilical cord). Research on treatment of liver fibrosis and development of cell transplantation therapy | Wide sources, low immunogenicity and anti-inflammatory properties | Low differentiation efficiency (< 30%) and insufficient functional maturity | | (Afshari et al. 2025; Lu et al. 2025; Zeilinger et al. 2016) |  |
| Pluripotent stem cells |  |  |  | |  |  |
| hESC | Separated from the cell mass in the embryo, differentiated into hepatic endoderm by Activin A, Wnt3a and other factors, and then matured into hepatocytes. Study on liver development mechanism, hereditary liver disease model | Unlimited proliferation ability, gene editing to build disease model | Ethical disputes, complicated differentiation process (> 20 days) and high cost. | | (Zeilinger et al. 2016; Vazin et al. 2010) |  |
| hiPSC | Somatic cells (such as skin fibroblasts) are reprogrammed into pluripotent stem cells and then differentiated into hepatocytes. Personalized drug toxicity test and patient-specific liver disease modelling | Avoid ethical issues and reflect the genetic background of patients (such as CYP2C19 polymorphism) | The differentiation efficiency fluctuates greatly and the maturity difference is significant (functional verification is required) | | (Rashid et al. 2010; E; Zeilinger et al. 2016) |  |
| liver-based *in vitro* drug metabolism model | | | | | |  |
| Recombinant enzyme | Expression of a single metabolic enzyme (such as CYP3A4, UGT1A1) | Clarify the specificity of metabolic enzymes; Used in the study of enzyme inhibition/induction mechanism. | Can not reflect the synergistic effect of multiple enzymes; Lack of physiological correlation; High specificity | | (Li et al. 2011; Friedberg et al. 1999) |  |
| Primary  hepatocyte | The natural hepatocytes isolated from the liver | Metabolic enzyme activity is close to the *in vivo* level; Preserve transporter function | Individual differences are large; Short survival time (< 7 days); High separation cost | | (Li et al. 2011; Zeilinger et al. 2016) |  |
| Immortalized hepatocytes | Such as HepG2 and Huh7 | Easy to obtain; Can be passed down for a long time; Suitable for long-term toxicity study | The expression level of metabolic enzymes is low (especially CYP 450); Lack of polarization characteristics of normal hepatocytes | | 2011; Aninat et al. 2006; Anthérieu et al. 2010; Zeilinger et al. 2016) |  |
| Stem cells induce hepatocytes | Multifunctional stem cells (such as iPSC) differentiate into hepatocyte-like cells. | Personalized source (patient specificity); Avoid ethical disputes; High metabolic enzyme activity potential | The differentiation efficiency is unstable; Maturity may be lower than that of primary hepatocytes; High cost and long cycle | | (Li et al. 2011; Zeilinger et al. 2016) |  |
| Liver microsome | Subcellular components isolated from hepatocytes are rich in metabolic enzymes such as CYP450 and UGT. | Low cost and simple operation; Suitable for metabolic stability test and enzyme phenotype analysis; Qualcomm screening potential | Lack of cell integrity (no transporter, cell membrane barrier); It is impossible to study the II binding reaction (coenzyme needs to be supplemented) | | (Li et al. 2011; Song et al. 2017) |  |
| Liver Cytosol | The supernatant of hepatocyte homogenate after removing microsomes by high-speed centrifugation (100,000×g) is rich in phase II metabolic enzymes (such as UGT, SULT, GST, etc.). | Dedicated to the study of phase II metabolism (such as glucuronidation and sulfation); Preserve the activity of soluble metabolic enzymes; Low cost and simple operation. | Lack of phase I metabolic enzymes (CYP450, etc.); Unable to study oxidation/reduction reaction; Possible residual cell debris interferes with detection. | | (Li et al. 2011; Liu et al. 2018) |  |
| S9 | The supernatant obtained by centrifugation after homogenization of liver tissue contains cytoplasm and microsomal enzymes. | Can study phase I and phase II metabolism at the same time; The experimental period is short | The enzyme activity decreased rapidly with time; It is impossible to simulate the process of cell uptake and excretion. | | (Li et al. 2011; Wang et al. 2020; Liu et al. 2018) |  |
| Precision liver section | Thin slice of liver tissue to keep the structure of liver intact | Preserve the interaction between cells and three-dimensional structure; Can study region-specific metabolism | High technical difficulty (need special slicing equipment); Activity maintenance time is limited (< 24 hours) | | (Li et al. 2011; Elferink et al. 2008) |  |
| Perfusion of isolated liver | The liver is placed in an external environment, and the perfusion fluid is used to simulate the physiological conditions *in vivo* to maintain the metabolic activity and function of the liver. | Simulate that situation of drugs *in vivo*; It avoids the risk of *in vivo* experiments on patients and conforms to ethical principles. | High technical requirements; Perfusion time is limited, usually only lasts for several hours to several days. | | (Li et al. 2011; Liu et al. 2018) |  |
| Liver-on-a-Chip | Organ system constructed in microfluidic device | Combined with other chips, it can simulate the interaction between organs (such as enterohepatic circulation); Dynamic microenvironment control | High cost; Standardization is difficult; Cell survival rate is affected by fluid shear stress. | | (Ghafoory et al. 2022; Liu et al. 2024; Liu et al. 2022) |  |
| Liver Bioreactor Systems | Extracorporeal perfusion device maintains the functional activity of liver tissue or cells (such as bioartificial liver system) | Support long-term metabolic research (several weeks); Close to physiological microenvironment (oxygen/nutrient gradient) | The equipment is complex and the maintenance cost is extremely high; High technical requirements for operation; It is difficult to achieve Qualcomm screening. | | (Tostões et al. 2012; Mustafa et al. 2018; Farzaneh et al. 2020) |  |
| *In vitro* intestinal flora model | | | | | |  |
| Batch fermentation model | The batch fermentation model simulates the intestinal microbiota in the short term by adding inoculum and substrate at once in a static anaerobic environment. | This model is simple to operate, low-cost, and highly flexible. | It cannot reflect dynamic changes *in vivo* or interactions with the host. | | (Nissen et al. 2020) |  |
| Dynamic fermentation model | The model simulates different segments of the intestine by using multistage series reactors, with continuous nutrient supply and precise environmental control to replicate intestinal dynamics in time and space. | This model is highly biomimetic, enables customization of complex intestinal ecology, is relatively easy to operate, and cost-controllable. | The equipment is complex, requires high maintenance, and demands a certain level of technical expertise. | | (Nissen et al. 2020; Gibson et al. 1988) |  |
| TNO *In Vitro* Model of the Colon | The model uses a four-chamber reactor with multiparameter computer control to simulate the physiological environment of the proximal colon, including temperature, anaerobiosis, mixing, pH, and metabolite removal. | This model closely replicates *in vivo* conditions, enables precise quantitative analysis, and offers high compatibility and reproducibility, making it suitable for nutrition and drug research. | The equipment is expensive, operation is complex, and experiments are time-consuming. | | (Meyer et al. 2004; Minekus 2015) |  |

**Supplementary 2. Cell models commonly used in excretion research**

| **Human origin** | **introduce** | **references** | **Animal origin** | **introduce** | **references** |
| --- | --- | --- | --- | --- | --- |
| IHKE-1 | Human renal proximal convoluted tubule epithelial cell line is often used to study the nephrotoxicity of drugs. The cell line showed a high level of permeability in mannitol permeation experiment, which indicated that it was not suitable for two-way transport research. | (Lechner et al. 2021) | LLC-PK1 | Epithelial cells of proximal convoluted tubule of pig kidney are often used to study the mechanism of drug action. This cell line expresses cytochrome P450 3A and 2B enzymes, and can metabolize drugs such as ifosfamide. | (Schinkel et al. 1997; Alvarez-Barrientos et al. 2001) |
| Caki-1 | Human renal cell line is often used to study the nephrotoxicity of drugs. The cell line showed a high level of permeability in mannitol permeation experiment, which indicated that it was not suitable for two-way transport research. | (Lechner et al. 2021) | OK | Epithelial cells of proximal convoluted tubule of American opossum kidney are often used to study the nephrotoxicity of drugs. The cell line showed early changes of mitochondrial membrane potential and cytoplasmic Ca2+ level after cadmium chloride treatment. | (Schinkel et al. 1997) |
| HK-2 | Human renal proximal convoluted tubule cells were isolated from adult male kidneys and immortalized with HPV16 E6/E7 virus. Its phenotype and functional markers, enzyme activity, membrane transport function and injury mechanism are similar to those of proximal tubular epithelial cells freshly isolated from rats. | (Jenkinson et al. 2012; Kim et al. 2002) | MDCKII | Canine renal epithelial cell line is often used to study the renal transport and toxicity of drugs. The cell line showed a high level of permeability in mannitol permeation experiment, but its expression patterns of markers and transporters were different from those of freshly isolated primary cells. | (Lechner et al. 2021) |
| RPTEC/TERT1 | Human renal proximal convoluted tubule epithelial cells, obtained by telomerase gene transfection, have good stability and function. | (Wieser et al. 2008) | NRK-52E | Rat renal proximal convoluted tubule epithelial cell line is often used to study the nephrotoxicity of drugs. The cell line showed a high level of permeability in mannitol permeation experiment, which indicated that it was not suitable for two-way transport research. | (Lechner et al. 2021) |
| ciPTEC | Conditionally immortalized human renal proximal convoluted tubule epithelial cells have good cell function and stability. | (Schinkel et al. 1997) |  |  |  |

**Supplementary 3. Cell lines for toxicity studies**

|  | **Cell strain/line** | **morphology** | **source** | **Species** | **references** |
| --- | --- | --- | --- | --- | --- |
| normal tissues | PHHs | liver cell | liver | Human |  |
|  | KCs | macrophage | liver | Human | (Elvevold et al. 2022) |
|  | hRPTEC | Proximal renal tubular cell | kidney | Human | (Jing et al. 2022) |
|  | MDCK | epithelioid cells | kidney | Dog | (Liu et al. 2014) |
|  | COS | fibroblast | kidney | African green monkey | (Liu et al. 2014) |
|  | Vero | epithelioid cells | kidney | African green monkey | (Liu et al. 2014) |
|  | IMR-90 | fibroblast | lung | Human | (Liu et al. 2014) |
|  | MRC-9 | fibroblast | lung | Human | (Liu et al. 2014) |
|  | WI-38 | fibroblast | lung | Human | (Liu et al. 2014) |
|  | MRC-5 | fibroblast | lung | Human | (Liu et al. 2014) |
|  | H9c2 | sarcoblast | heart | rat | (Liu et al. 2014) |
| tumor tissues | Huh7 | epithelioid cells | Hepatocellular tumor | Human | (Schicht et al. 2022) |
|  | HepG2 | epithelioid cells | Hepatocellular tumor | Human | (Liu et al. 2014) |
|  | HepaRG | epithelioid cells | Hepatocellular tumor | Human | (Liu et al. 2014) |
|  | HLE | epithelioid cells | Hepatocellular tumor | Human |  |
|  | C3A | epithelioid cells | Hepatocellular tumor | Human | (Li et al. 2020) |
|  | HL-60 | epithelioid cells | Myeloid leukemia | Human | (Liu et al. 2014) |
|  | MCF-7 | epithelioid cells | Breast cancer pleura | Human | (Liu et al. 2014) |

**Supplementary 4.** **Application of organoid in toxicity study of TCM (Supplementary)**

| **references** | **source** | **compound** | **Chip model** | **application** | **result** |
| --- | --- | --- | --- | --- | --- |
| (Li et al. 2017) | *Pleuropterus multiflorus (Thunb.) Nakai* | cis-stilbene glycosides | Liver organoids model constructed by HepG2 cells | To evaluate the liver injury effect of cis-stilbene glycosides (cis-SG), a susceptible substance of Polygonum multiflorum Thunb. | The hepatotoxicity of cis-stilbene glycosides was detected (IC50 of single administration was 1.9 times that of cyclosporine), and its toxicity was significantly higher than that of trans isomer (IC50 of trans-SG was 4.1 times higher), and the IC50 was further reduced by repeated exposure. |
| (Lee et al. 2025) | sea urchin | Echinochrome A | Heart organoids derived from human induced pluripotent stem cells | To evaluate the cardiotoxicity of Echinochrome A. | Echinochrome A did not cause electrophysiological abnormalities (pulsatile phase, amplitude of field potential, etc.), cytotoxicity or contraction dysfunction (no significant change in contraction/relaxation speed) in the concentration range of 0.1-30 μM, and the functions of key ion channels (Cav1.2, Nav1.5, hERG) were stable, which confirmed its cardiac safety. |
| (Wu et al. 2023) | *Citri Reticulatae Pericarpium* | nobiletin | Intestinal organoids constructed by small intestinal crypt in mice | Inhibitory effect of effective dose of nobiletin on intestinal stem cells | *In vitro* model (50-200 μM), nobiletin significantly inhibited the germination and growth of intestinal organs (dose-dependent, P<0.001), and its inhibitory effect could be partially reversed by activating Wnt pathway. |

**Supplementary 5. Application of OoC technology in TCM ADMET research**

| **references** | **source** | **compound** | **Chip model** | **application** | **result** |
| --- | --- | --- | --- | --- | --- |
| (Xu et al. 2024) | *Aconitum carmichaelii Debeaux* | Aconitine | Heart-on-chip models of cardiomyocytes (HL-1) and human umbilical vein endothelial cells (HUVEC) related to cardiac function. | To study the mechanism of cardiotoxicity of Aconitine | Aconitine induces membrane structure changes and releases lactate dehydrogenase (LDH) to cause calcium overload. Through LC‒MS detection, it was found that Aconitine affected the level of TCA cycle metabolites. |
| (Shi et al. 2023) | *Sophora flavescens Aiton*， etc. | Matrine；Baicalein；Osthole；Paeoniflorin；Resveratrol； Quercetin | Blood‒brain barrier (BBB) composed of primary human brain microvascular endothelial cells, pericytes, astrocytes and glioma cells and microfluidic chip (BBB-U251 chip) for glioma microenvironment. | Study on TCM components crossing blood‒brain barrier and activity evaluation. | The results of permeability coefficient of compounds in BBB-on-Chip are closer to the *in vivo* data than Transwell model. Because of the existence of BBB, the effect of drugs on U251 cells in BBB-U251 chip was significantly reduced. |
| (Li et al. 2023) | *Panax ginseng C. A. Mey.* | CK, Rh2 (S), Rg3 (S), PPD (S)Ginsenosides and Their Metabolites | Microfluidic coculture system: upper layer cultured hepatocytes (HepG2 cells); Three cell lines, human non-small cell lung cancer cells (A549), human breast cancer cells (MCF-7) and human normal hepatocytes (HL7702), were cultured in the lower layer. | To evaluate the inhibitory effect of ginsenoside and its metabolites on different tumor cell lines and the effect of liver metabolism on drug activity. | The metabolites of Rg3 (S), Rh2 (S) and CK ginsenoside in the chip were detected by HPLC/TOF-MS analysis. It is analysed that the liver undergoes orderly deglycosylation and oxidation during metabolism. The metabolites Rh2 (S) and PPD (S) of Rg3 (S) showed stronger antitumour. |
| (Cai 2019) | Sophora flavescens Aiton， etc. | Oxymatrine；Evodiamine；Oleic Acid；Nitidine Chloride | Microfluidic chip model for simulating normal hepatic sinus structure | To compare the effects of different concentrations of TCM components on cell activity in order to evaluate the hepatotoxicity of drugs. | The IC50 value obtained by microfluidic cultured hepatic lobular chip was significantly higher than that measured by orifice plate experiment. The upper structure of the chip and the lower Hep G2 cell culture cavity which may resist hepatotoxic substances from entering to some extent. |
| (Zhu et al. 2021) | Guanxinning injection (GXNI), Shenfu injection (SFI) and Xiangdan injection (XDI) | Salvianolic acid B；Ginsenosides；Pterostilbene，ect | Liver microspheres constructed from human umbilical vein endothelial cells (EA.hy926) and Hep G2 cells | Re-evaluation of liver safety of TCM injection | After 14 days of culture, the liver microspheres printed in 3D can still maintain normal expression of ALB and CYP3A4. Xiangdan injection (XDI) may have some effects on the liver after long-term and high-dose use. |
| (Liu et al. 2020) | *Panax ginseng C. A. Mey.* | Ginsenoside Compound k(CK) | Multiple single organ chips (Caco-2, HUVEC, HepG2 and HK-2 cells) including intestinal tract, blood vessel, liver and kidney chips are connected to simulate the physiological environment of human body. | To evaluate the absorption, metabolism and toxicity of CK | Under dynamic conditions, CK absorption efficiency is high, and Caco-2 and HepG2 cells have strong uptake ability; The dynamic metabolism of HepG2 cells increased, and the tolerance of cells to CK increased and the toxicity decreased. In the multiorgan chip, CK has little toxicity to liver and kidney cells and mainly remains in the intestine. |

Afshari, A., N. Azarpira, and S. Pakbaz. 2025. 'Differentiation of Wharton's jelly-derived mesenchymal stromal cells into hepatocyte-like cells using a refined method', BMC Mol Cell Biol, 26: 9.

Alvarez-Barrientos, A., J. E. O'Connor, R. Nieto Castillo, A. B. Moreno Moreno, and P. Prieto. 2001. 'Use of flow cytometry and confocal microscopy techniques to investigate early CdCl(2)-induced nephrotoxicity in vitro', Toxicol In Vitro, 15: 407-12.

Aninat, C., A. Piton, D. Glaise, T. Le Charpentier, S. Langouët, F. Morel, et al. 2006. 'Expression of cytochromes P450, conjugating enzymes and nuclear receptors in human hepatoma HepaRG cells', Drug Metab Dispos, 34: 75-83.

Anthérieu, S., C. Chesné, R. Li, S. Camus, A. Lahoz, L. Picazo, et al. 2010. 'Stable expression, activity, and inducibility of cytochromes P450 in differentiated HepaRG cells', Drug Metab Dispos, 38: 516-25.

Bale, S. S., L. Vernetti, N. Senutovitch, R. Jindal, M. Hegde, A. Gough, et al. 2014. 'In vitro platforms for evaluating liver toxicity', Exp Biol Med (Maywood), 239: 1180-91.

Banker, M. J., T. H. Clark, and J. A. Williams. 2003. 'Development and validation of a 96-well equilibrium dialysis apparatus for measuring plasma protein binding', J Pharm Sci, 92: 967-74.

Barré, J., J. M. Chamouard, G. Houin, and J. P. Tillement. 1985. 'Equilibrium dialysis, ultrafiltration, and ultracentrifugation compared for determining the plasma-protein-binding characteristics of valproic acid', Clin Chem, 31: 60-4.

Bussolari, S. R., C. F. Dewey, Jr., and M. A. Gimbrone, Jr. 1982. 'Apparatus for subjecting living cells to fluid shear stress', Rev Sci Instrum, 53: 1851-4.

Butt, M. A. 2025. 'Surface Plasmon Resonance-Based Biodetection Systems: Principles, Progress and Applications-A Comprehensive Review', Biosensors (Basel), 15.

Cai, Le. 2019. 'Hepatotoxicity evaluation of traditional Chinese herb componentsbased on the liver-on-a-chip', Master's thesis, Dalian University of Technology.

Campard, D., P. A. Lysy, M. Najimi, and E. M. Sokal. 2008. 'Native umbilical cord matrix stem cells express hepatic markers and differentiate into hepatocyte-like cells', Gastroenterology, 134: 833-48.

Chen, J., C. Ohnmacht, and D. S. Hage. 2004. 'Studies of phenytoin binding to human serum albumin by high-performance affinity chromatography', J Chromatogr B Analyt Technol Biomed Life Sci, 809: 137-45.

Chen, L. , H. Dong, J. Zheng, T. Zheng, and W. Song. 2016. 'Progress in Research on the Application of Ussing Chamber', CMD, 31: 73-77.

Choucha-Snouber, L., C. Aninat, L. Grsicom, G. Madalinski, C. Brochot, P. E. Poleni, et al. 2013. 'Investigation of ifosfamide nephrotoxicity induced in a liver-kidney co-culture biochip', Biotechnol Bioeng, 110: 597-608.

Dianat, N., C. Steichen, L. Vallier, A. Weber, and A. Dubart-Kupperschmitt. 2013. 'Human pluripotent stem cells for modelling human liver diseases and cell therapy', Curr Gene Ther, 13: 120-32.

Elferink, M. G., P. Olinga, A. L. Draaisma, M. T. Merema, S. Bauerschmidt, J. Polman, et al. 2008. 'Microarray analysis in rat liver slices correctly predicts in vivo hepatotoxicity', Toxicol Appl Pharmacol, 229: 300-9.

Elvevold, K., I. Kyrrestad, and B. Smedsrød. 2022. 'Protocol for Isolation and Culture of Mouse Hepatocytes (HCs), Kupffer Cells (KCs), and Liver Sinusoidal Endothelial Cells (LSECs) in Analyses of Hepatic Drug Distribution', Methods Mol Biol, 2434: 385-402.

Faria, A., D. Pestana, D. Teixeira, J. Azevedo, V. De Freitas, N. Mateus, et al. 2010. 'Flavonoid transport across RBE4 cells: A blood-brain barrier model', Cell Mol Biol Lett, 15: 234-41.

Farzaneh, Z., S. Abbasalizadeh, M. H. Asghari-Vostikolaee, M. Alikhani, J. M. S. Cabral, and H. Baharvand. 2020. 'Dissolved oxygen concentration regulates human hepatic organoid formation from pluripotent stem cells in a fully controlled bioreactor', Biotechnol Bioeng, 117: 3739-56.

Fung, E. N., Y. H. Chen, and Y. Y. Lau. 2003. 'Semi-automatic high-throughput determination of plasma protein binding using a 96-well plate filtrate assembly and fast liquid chromatography-tandem mass spectrometry', J Chromatogr B Analyt Technol Biomed Life Sci, 795: 187-94.

Ghafoory, S., C. Stengl, S. Kopany, M. Mayadag, N. Mechtel, B. Murphy, et al. 2022. 'Oxygen Gradient Induced in Microfluidic Chips Can Be Used as a Model for Liver Zonation', Cells, 11.

Guo, L., S. Dial, L. Shi, W. Branham, J. Liu, J. L. Fang, et al. 2011. 'Similarities and differences in the expression of drug-metabolizing enzymes between human hepatic cell lines and primary human hepatocytes', Drug Metab Dispos, 39: 528-38.

Jagtiani, E., M. Yeolekar, S. Naik, and V. Patravale. 2022. 'In vitro blood brain barrier models: An overview', J Control Release, 343: 13-30.

Jenkinson, S. E., G. W. Chung, E. van Loon, N. S. Bakar, A. M. Dalzell, and C. D. Brown. 2012. 'The limitations of renal epithelial cell line HK-2 as a model of drug transporter expression and function in the proximal tubule', Pflugers Arch, 464: 601-11.

Jing, B., L. Yan, J. Li, P. Luo, X. Ai, and P. Tu. 2022. 'Functional Evaluation and Nephrotoxicity Assessment of Human Renal Proximal Tubule Cells on a Chip', Biosensors (Basel), 12.

Kim, D., S. H. Garrett, M. A. Sens, S. Somji, and D. A. Sens. 2002. 'Metallothionein isoform 3 and proximal tubule vectorial active transport', Kidney Int, 61: 464-72.

Lechner, C., U. Mönning, A. Reichel, and G. Fricker. 2021. 'Potential and Limits of Kidney Cells for Evaluation of Renal Excretion', Pharmaceuticals (Basel), 14.

Li, D., Y. Han, T. Yu , X. Meng, Q. Yu, J. Wang, et al. 2011. 'Chinese Journal of Clinical Pharmacology and Therapeutics', Chin. J. Clin. Pharmacol. Ther., 16: 688-94.

Li, Z. H., Z. Y. Xie, X. X. Ouyang, K. Z. Huang, X. P. Yu, Y. L. Zhao, et al. 2020. 'Assessment of biological functions for C3A cells interacting with adverse environments of liver failure plasma', Hepatobiliary Pancreat Dis Int, 19: 129-37.

Li, Z., J. Li, M. Sun, L. Men, E. Wang, Y. Zhao, et al. 2023. 'Analysis of metabolites and metabolism-mediated biological activity assessment of ginsenosides on microfluidic co-culture system', Front Pharmacol, 14: 1046722.

Liu, D., S. Jiao, J. Wei, X. Zhang, Y. Pei, Z. Pei, et al. 2020. 'Investigation of absorption, metabolism and toxicity of ginsenosides compound K based on human organ chips', Int J Pharm, 587: 119669.

Liu, J., Y. Du, X. Xiao, D. Tan, Y. He, and L. Qin. 2024. 'Construction of in vitro liver-on-a-chip models and application progress', Biomed Eng Online, 23: 33.

Liu, J., C. Feng, M. Zhang, F. Song, and H. Liu. 2022. 'Design and Fabrication of a Liver-on-a-chip Reconstructing Tissue-tissue Interfaces', Front Oncol, 12: 959299.

Liu, L., and J. Zhang. 2018. 'Research progress of drug hepatic metabolism models in vitro', Chin. J. Vet. Sci., 38: 2015-19.

Liu, R., Y. Xie , G. Pan, Z. Liu, and B. Zhang. 2007. 'Research progress on determination methods of drug plasma protein binding rate', Tianjin J. Tradit. Chin. Med: 526-28.

Liu , T., C. Guo, and X. ZHao 2014. 'In vitro cytotoxicity evaluation in toxicology', CBLS, 26: 319-24.

Liu, Y., and S. Zeng. 2008. 'Advances in the MDCK-MDR1 cell model and its applications to screen drug permeability', Acta Pharmaceutica Sinica: 559-64.

Lu, P., Z. Meng, W. Wang, T. Liu, X. Wang, X. Bi, et al. 2007. 'The Surface plasmon resonance technique measures drug-Serum albumin interactions', TAAP: 147-51.

Lu, X., H. Hu, Y. Zhou, H. Zhang, C. Xie, Y. Sun, et al. 2025. 'One-step engineered mesenchymal stem cell-derived exosomes against hepatic ischemia-reperfusion injury', Int J Pharm, 672: 125292.

Martínez-Pla, J. J., M. A. Martínez-Gómez, Y. Martín-Biosca, S. Sagrado, R. M. Villanueva-Camañas, and M. J. Medina-Hernández. 2004. 'High-throughput capillary electrophoresis frontal analysis method for the study of drug interactions with human serum albumin at near-physiological conditions', Electrophoresis, 25: 3176-85.

Mustafa, Malik G., Md Gulam M. Khan, Duy Nguyen, and Shahid Iqbal. 2018. 'Chapter 13 - Techniques in Biotechnology: Essential for Industry.' in Debmalya Barh and Vasco Azevedo (eds.), Omics Technologies and Bio-Engineering (Academic Press).

Ostergaard, J., C. Schou, C. Larsen, and N. H. Heegaard. 2003. 'Effect of dextran as a run buffer additive in drug-protein binding studies using capillary electrophoresis frontal analysis', Anal Chem, 75: 207-14.

Pleguezuelos-Manzano, C., J. Puschhof, S. van den Brink, V. Geurts, J. Beumer, and H. Clevers. 2020. 'Establishment and Culture of Human Intestinal Organoids Derived from Adult Stem Cells', Curr Protoc Immunol, 130: e106.

Rashid, S. T., S. Corbineau, N. Hannan, S. J. Marciniak, E. Miranda, G. Alexander, et al. 2010. 'Modeling inherited metabolic disorders of the liver using human induced pluripotent stem cells', J Clin Invest, 120: 3127-36.

Schicht, G., L. Seidemann, R. Haensel, D. Seehofer, and G. Damm. 2022. 'Critical Investigation of the Usability of Hepatoma Cell Lines HepG2 and Huh7 as Models for the Metabolic Representation of Resectable Hepatocellular Carcinoma', Cancers (Basel), 14.

Schinkel, A. H., U. Mayer, E. Wagenaar, C. A. Mol, L. van Deemter, J. J. Smit, et al. 1997. 'Normal viability and altered pharmacokinetics in mice lacking mdr1-type (drug-transporting) P-glycoproteins', Proc Natl Acad Sci U S A, 94: 4028-33.

Schwartz, R. E., M. Reyes, L. Koodie, Y. Jiang, M. Blackstad, T. Lund, et al. 2002. 'Multipotent adult progenitor cells from bone marrow differentiate into functional hepatocyte-like cells', J Clin Invest, 109: 1291-302.

Shen, Q., Z. Jiang, L. ZHang, and X. Huang. 2018. 'Advances in models for predicting drug intestinal permeability', Acta Pharm. Sin., 53: 727-34.

Shi, Y., X. He, H. Wang, J. Dai, J. Fang, Y. He, et al. 2023. 'Construction of a novel blood brain barrier-glioma microfluidic chip model: Applications in the evaluation of permeability and anti-glioma activity of traditional Chinese medicine components', Talanta, 253: 123971.

Siddharthan, V., Y. V. Kim, S. Liu, and K. S. Kim. 2007. 'Human astrocytes/astrocyte-conditioned medium and shear stress enhance the barrier properties of human brain microvascular endothelial cells', Brain Res, 1147: 39-50.

Snykers, S., J. De Kock, V. Tamara, and V. Rogiers. 2011. 'Hepatic differentiation of mesenchymal stem cells: in vitro strategies', Methods Mol Biol, 698: 305-14.

Song, S., Y. Bai, and J. Liang. 2023. 'Application progress of parallel artificial membrane permeation assay model in drug permeability screening of transdermal drug delivery system', Chin. Pharm. , 34: 502-07.

Song Sankong , Bai Yinliang , and Liang Jiandi. 2023. 'Application progress of parallel artificial membrane permeation assay model in drug permeability screening of transdermal drug delivery system', China Pharmacy, 34: 502-07.

Sophianopoulos, J. A., S. J. Durham, A. J. Sophianopoulos, H. L. Ragsdale, and W. P. Cropper, Jr. 1978. 'Ultrafiltration is theoretically equivalent to equilibrium dialysis but much simpler to carry out', Arch Biochem Biophys, 187: 132-7.

Tarbell, J. M. 2010. 'Shear stress and the endothelial transport barrier', Cardiovasc Res, 87: 320-30.

Tostões, R. M., S. B. Leite, M. Serra, J. Jensen, P. Björquist, M. J. Carrondo, et al. 2012. 'Human liver cell spheroids in extended perfusion bioreactor culture for repeated-dose drug testing', Hepatology, 55: 1227-36.

van Eck, N. J., and L. Waltman. 2010. 'Software survey: VOSviewer, a computer program for bibliometric mapping', Scientometrics, 84: 523-38.

Vazin, T., and W. J. Freed. 2010. 'Human embryonic stem cells: derivation, culture, and differentiation: a review', Restor Neurol Neurosci, 28: 589-603.

Veszelka, S., A. Tóth, F. R. Walter, A. E. Tóth, I. Gróf, M. Mészáros, et al. 2018. 'Comparison of a Rat Primary Cell-Based Blood-Brain Barrier Model With Epithelial and Brain Endothelial Cell Lines: Gene Expression and Drug Transport', Front Mol Neurosci, 11: 166.

Wang, H., S. Xu, W. CHu, Z. Gui , and G. Huang. 2018. 'Application of Microdialysis Technology in Pharmaceutical Research', Strait Pharm. J., 30: 30-33.

Weksler, B., I. A. Romero, and P. O. Couraud. 2013. 'The hCMEC/D3 cell line as a model of the human blood brain barrier', Fluids Barriers CNS, 10: 16.

Wieser, M., G. Stadler, P. Jennings, B. Streubel, W. Pfaller, P. Ambros, et al. 2008. 'hTERT alone immortalizes epithelial cells of renal proximal tubules without changing their functional characteristics', Am J Physiol Renal Physiol, 295: F1365-75.

Wilkening, S., F. Stahl, and A. Bader. 2003. 'Comparison of primary human hepatocytes and hepatoma cell line Hepg2 with regard to their biotransformation properties', Drug Metab Dispos, 31: 1035-42.

Wu, Y., H. Liu, and J. Ni. 2011. 'Advances in parallel artificial membrane permeability assay and its applications', Acta Pharm. Sin., 46: 890-95.

Xiang, T., J. Wang, and H. Li. 2024. 'Current applications of intestinal organoids: a review', Stem Cell Res Ther, 15: 155.

Xu, T., Z. Wu, H. Yao, Y. Zhang, S. Chen, Y. Li, et al. 2024. 'Evaluation of aconitine cardiotoxicity with a heart-on-a-particle prepared by a microfluidic device', Chem Commun (Camb), 60: 4898-901.

Zeilinger, K., N. Freyer, G. Damm, D. Seehofer, and F. Knöspel. 2016. 'Cell sources for in vitro human liver cell culture models', Exp Biol Med (Maywood), 241: 1684-98.

Zheng, X. , Y. Liu, X. Jia, M. Dong, and X. Li 2021. 'The application of SPR in drug research', J. Pharm. Res., 40: 196-98+205.

Zhu, L., H. Du, Y. He, M. Yu, D. Zhang, J. Yang, et al. 2021. 'Reevaluation of liver safety of commonly used Chinese medicine injection based on bio-printing 3D cell microfluidic chip', Chung-nan Yao Hsueh, 19: 2304-10.
